# Synthesis and investigation of donor–porphyrin–acceptor triads with long-lived photo-induced charge-separate states†
†Electronic supplementary information (ESI) available. See DOI: 10.1039/c5sc01830g


**DOI:** 10.1039/c5sc01830g

**Published:** 2015-07-31

**Authors:** Julien B. Kelber, Naitik A. Panjwani, Di Wu, Rafael Gómez-Bombarelli, Brendon W. Lovett, John J. L. Morton, Harry L. Anderson

**Affiliations:** a Oxford University , Chemistry Research Laboratory , 12 Mansfield Road , OX1 3TA , Oxford , UK . Email: harry.anderson@chem.ox.ac.uk; b University College London , London Centre for Nanotechnology , Gower Place , WC1E 6BT , London , UK . Email: jjl.morton@ucl.ac.uk; c Harvard University , Department of Chemistry and Chemical Biology , 12 Oxford St. 02138 , Cambridge , MA , USA; d University of St Andrews , SUPA , School of Physics and Astronomy , KY16 9SS , St Andrews , UK . Email: bwl4@st-andrews.ac.uk

## Abstract

Two donor–porphyrin–acceptor triads have been synthesized using a versatile Suzuki-coupling route. This synthetic strategy allows the powerful donor tetraalkylphenylenediamine (**TAPD**) to be introduced into tetraarylporphyrin-based triads without protection. The thermodynamics and kinetics of electron transfer in the new triads are compared with a previously reported octaalkyldiphenyl-porphyrin triad exhibiting a long-lived spin-polarized charge separate state (CSS), from theoretical and experimental perspectives, in both fluid solution and in a frozen solvent glass. We show that the less favorable oxidation potential of the tetraaryl-porphyrin core can be offset by using **C_60_**, as a better electron-acceptor than triptycenenaphthoquinone (**TNQ**). The **C_60_**–porphyrin–**TAPD** triad gives a spin-polarized charge-separated state that can be observed by EPR-spectroscopy, with a mean lifetime of 16 ms at 10 K, which is longer than in the previously reported **TNQ**–porphyrin–**TAPD** triad, following the predicted trend from calculated charge-recombination rates.

## Abstract 2

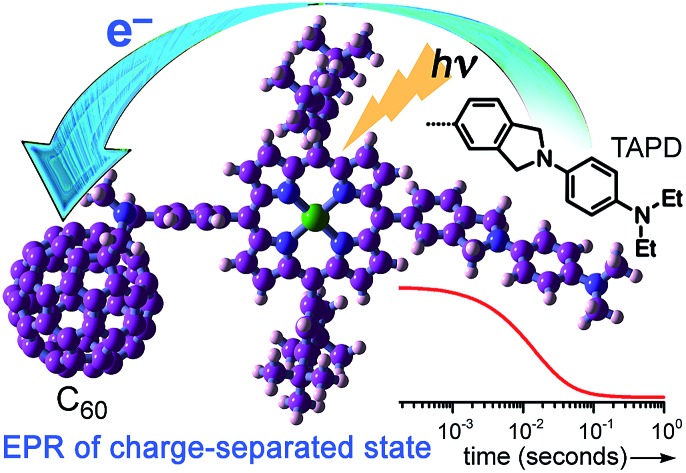
The powerful electron donor tetraalkylphenylenediamine (**TAPD**) facilitates photo-induced electron transfer, even in a frozen solvent at 10 K, generating a long-lived spin-polarized charge separate state which can be observed by EPR.

## Introduction

Photo-induced intramolecular electron transfer can generate a charge-separated state (CSS) consisting of a hole and an electron with a spatial separation of 1–3 nm.1*a* In most cases, the hole and electron recombine rapidly (from ps to ns) to regenerate the ground-state. However, in some cases, the CSS can have a longer lifetime^1^ (from μs up to possibly hours1h,i), allowing chemical, physical or biological processes to exploit its high energy and unusual electronic structure.

Long-lived photo-excited CSSs are important for a variety of applications. They are studied to understand and mimic electron transfer in natural photosynthesis, in which energy from sunlight is converted into chemical potential.^2^ In the area of quantum information processing, control of the spin dynamics of a CSS may allow the manipulation of a nuclear or electronic spin, to encode or transfer information.^3^ It is also thought that some birds, such as the European robin, use the magnetic field-dependence of the recombination rate of a CCS to orient themselves in the earth's magnetic field. Mimicking this avian compass may enable small magnetic fields to be detected.^4^

In the high-temperature limit, where solvent dynamics and nuclear motions can be treated as classical harmonic oscillators, recombination rates of CSSs are given by the Marcus equation^5^ (eqn (1)),1
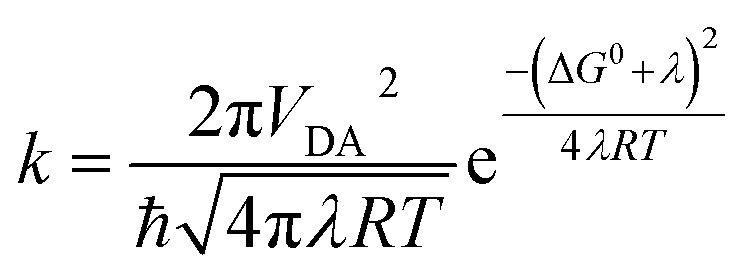
where *V*_DA_ is the donor/acceptor coupling matrix element, Δ*G*^0^ is the free energy change, *λ* is the global reorganization energy, *T* is the temperature, *ℏ* is the reduced Planck constant and *R* is the ideal gas constant. In order to reduce the rate of back-electron transfers and create long-lived CSSs, chemists have attempted to reduce the *V*_DA_ coupling term by increasing the distance between the photo-generated charges, by designing molecular dyads, triads and pentads.^6^

**Fig. 1:**
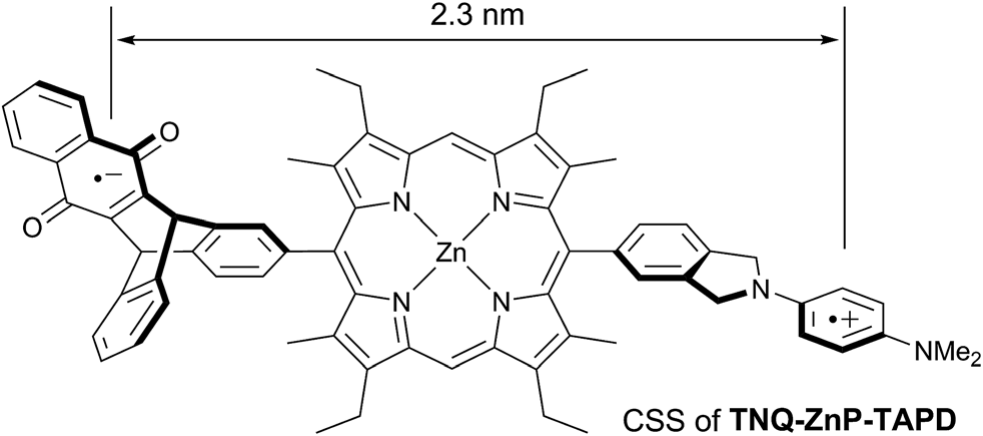
Wasielewski's design of a long-lived photo-generated radical pair.^7^

Wasielewski *et al.*^7^ designed a donor–porphyrin–acceptor triad **TNQ–ZnP–TAPD** (Fig. 1) with three desirable features. First, the strong electron-donating and -accepting behavior of the tetraalkylphenylenediamine (**TAPD**) and triptycenenaphthoquinone (**TNQ**) moieties make charge separation favorable, even in a frozen solvent where the solvent cannot reorganize to stabilize the photo-generated zwitterion. Secondly, the produced electron/hole pair has no through π-bond electronic coupling (because the donor and the acceptor moieties are separated by isolating methylene bridges), and weak through-σ-bond coupling, due to the near-orthogonal porphyrin core. Finally, the charges are rigidly separated by a distance of 2.3 nm, limiting through-space coupling. These last two features yield a very small *V*_DA_ and consequently extend the lifetime of the CSS. **TNQ–ZnP–TAPD** showed a CSS with a lifetime of 4 ms, together with a spin-polarized radical-pair that closely mimics the bacteriochlorophyll cation–quinone anion pair found in photosynthetic reaction centers.^8^

The initial objective of the work presented here was to synthesize porphyrin-based triads exhibiting long-lived CSSs, such as **TNQ–ZnP–TAPD**, so that they could be used for quantum information storage experiments. Since we also wanted to modulate the properties of our triads, we sought a versatile synthetic route with few steps from accessible precursors, which would tolerate a wide range of functional groups.

**Scheme 1:**
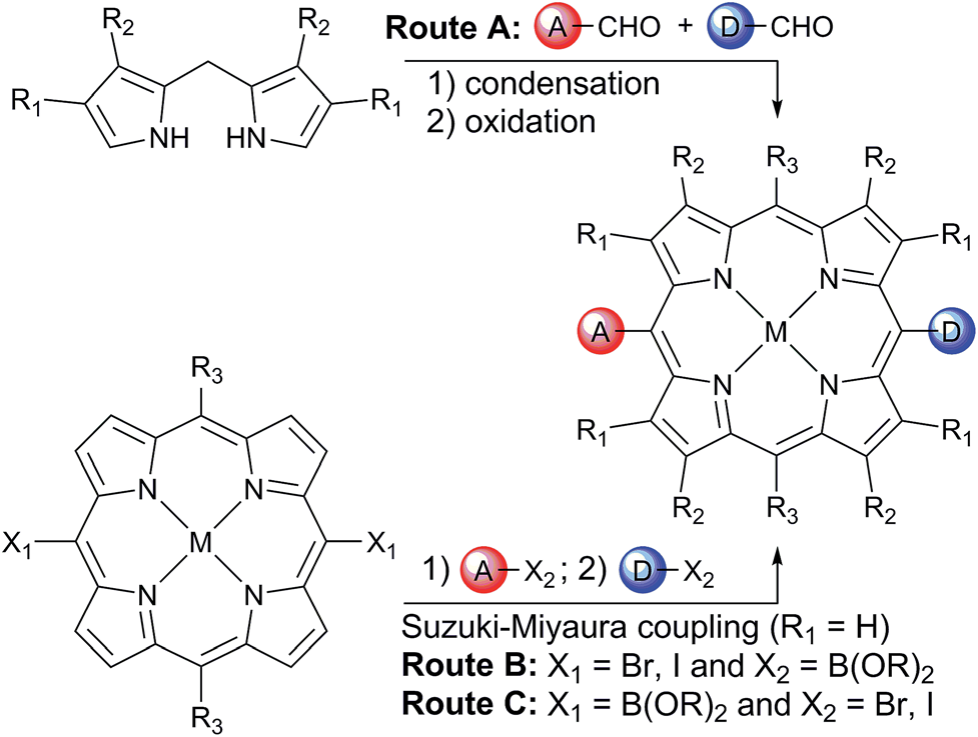
Two synthetic routes towards 5-acceptor–15-donorporphyrins.

In order to avoid aggregation and enhance the solubility of 5,15-diarylporphyrins, two positions are available to introduce solubilizing groups (Scheme 1). Porphyrins can be substituted on the β-pyrrole positions (R_1_ and R_2_ on Scheme 1), generally with aliphatic chains, providing a locked 80–90° dihedral angle between the porphyrin and the aryls groups. However, β-substituted pyrroles are less readily available than pyrrole, and the steric hindrance at the 5 and 15 *meso*-positions prevents cross-coupling strategies from being used to introduce the donor and acceptor moieties.

Another well-established strategy to avoid aggregation and enhance solubility in 5,15-diarylporphyrins is to introduce bulky aryl groups in the two remaining 10 and 20 *meso*-positions (R_3_ = Ar; R_1_ = R_2_ = H on Scheme 1). This substitution pattern avoids steric hindrance around the 5 and 15 positions.

**TAPD** derivatives, also known as Würster blue,^9^ are highly electron-rich (oxidation potential: –0.24 V *vs.* Fc/Fc^+^, see later) and are therefore promising electron donors. On the other hand, their low oxidation potentials makes them reactive towards oxygen and other oxidants, such as those used in porphyrin synthesis.

**Scheme 2:**
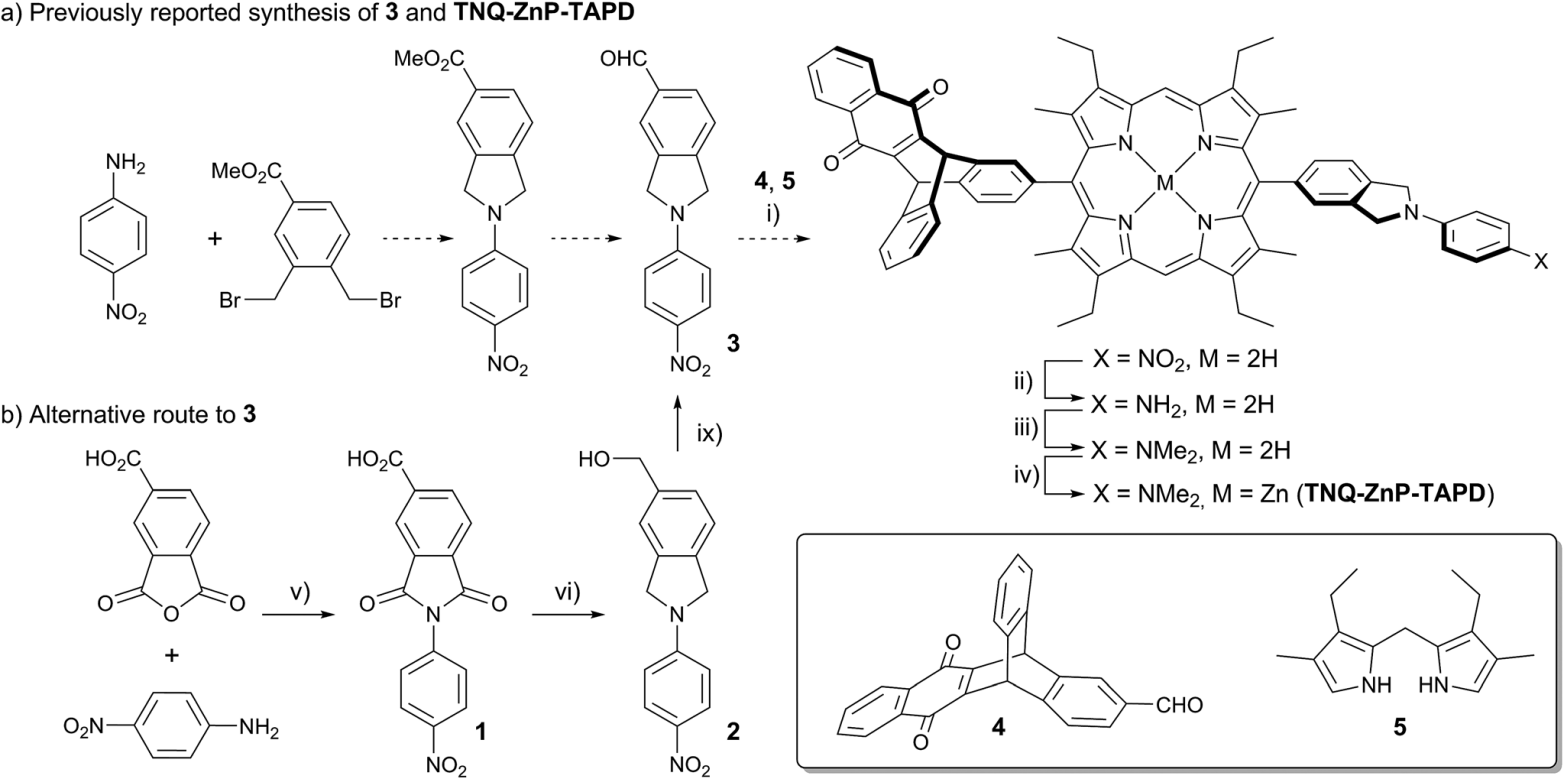
(a) Reported synthetic route of **TNQ–ZnP–TAPD** using the protected **TAPD** precursor **3**.7a,b (i) BF_3_·OEt_2_, DCM then DDQ; (ii) SnCl_2_, HCl; (iii) NaBH_3_CN, CH_2_O; (iv) Zn(OAc)_2_, CHCl_3_/MeOH; (b) new synthesis of **3**. (v) AcOH, 12 h, 118 °C, 95%; (vi) BH_3_·THF, THF, 12 h, 66 °C, 75%; (vii) activated MnO_2_, CHCl_3_, 15 min, 25 °C, 70%.

One retro-synthetic route to 5-acceptor–15-donor disubstituted porphyrin triads is the statistical condensation of a dipyrromethane with two aldehydes substituted with the donor and acceptor moieties (Scheme 1, top, Route A), and subsequent oxidation of the porphyrinogen, typically with DDQ or chloranil. This route is incompatible with the use of oxidation-sensitive donors such as **TAPD**. Indeed, isoindolines are known to be oxidized to isoindoles, which then react further *via* dimerization or Diels–Alder reactions.^10^ Therefore the synthesis of **TNQ–ZnP–TAPD** required masking of the terminal dimethylamine as a nitro-group, resulting in a less convergent route (Scheme 2a).7*a*

Alternatively, a symmetrical 5,15-diaryl-10,20-dibromoporphyrin^11^ can be synthesized, and functionalized with two different moieties *via* successive cross-coupling reactions (Scheme 1, bottom). Suzuki–Miyaura coupling is widely used as a mild, non-toxic, and efficient approach for the convergent synthesis of aromatic molecular materials,^12^ including porphyrin derivatives.^13^ In this case, Suzuki–Miyaura coupling allows not only an efficient synthesis of asymmetrical donor–porphyrin–acceptor triads, but also the introduction of the sensitive donor moiety in the very last step, as the mild conditions do not require the donor to be protected from oxidation.

**Scheme 3:**
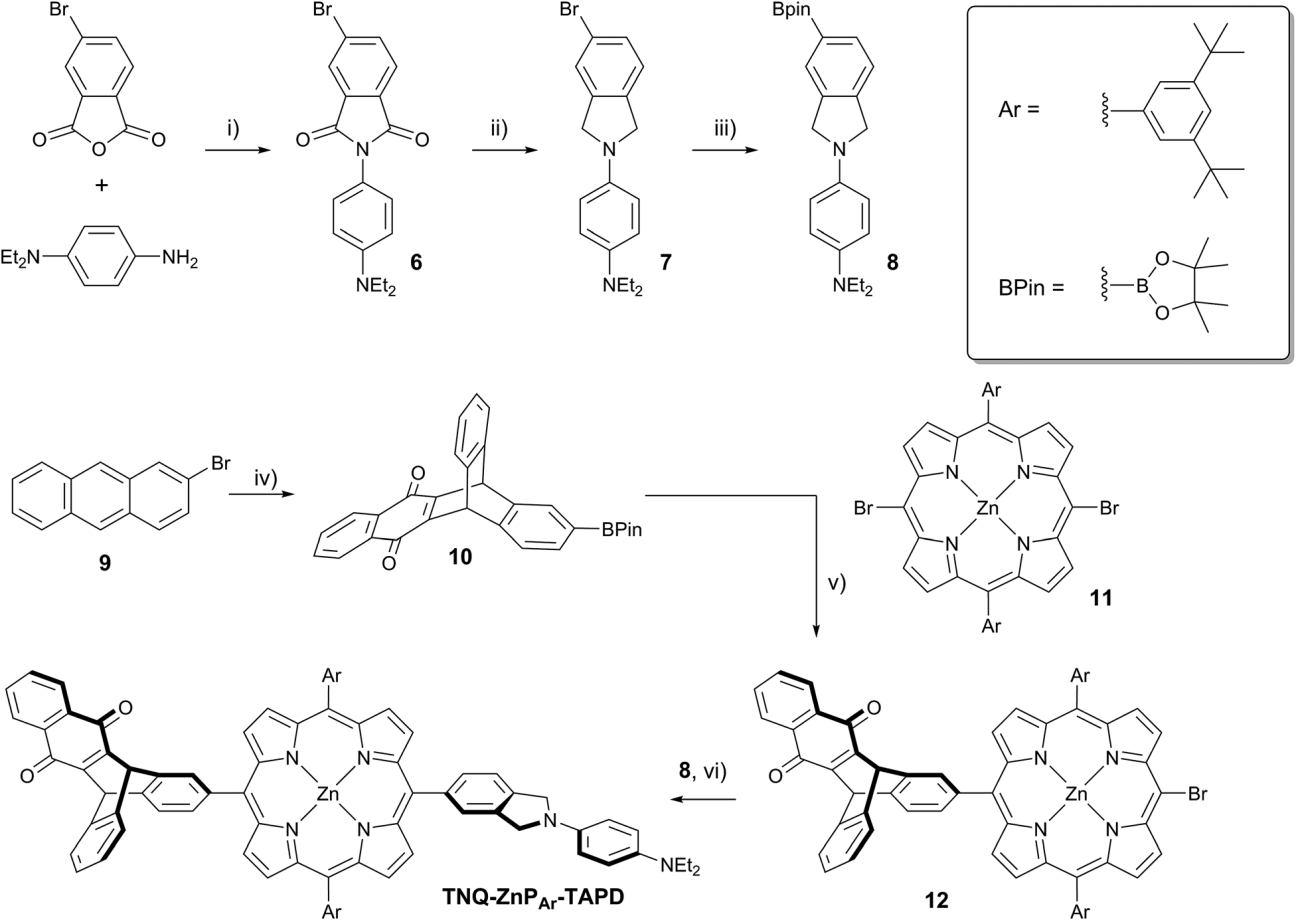
(i) AcOH, 12 h, 118 °C, 95%; (ii) BH_3_·THF, THF, 12 h, 66 °C, 60%; (iii) (BPin)_2_, PdCl_2_(dppf)·DCM, AcOK, DMF, 7 h, 90 °C, 79%; (iv) 1,4-naphthoquinone, PhNO_2_, 3 d, 140 °C, then (iii) 55% over 2 steps; (v) 3 eq. of **11**, Cs_2_CO_3_, Pd(PPh_3_)_4_, PhMe/pyridine, 2 d, 110 °C, 69%; (vi) Cs_2_CO_3_, Pd(PPh_3_)_4_, PhMe/pyridine, 4 h, 110 °C, 72%.

Here we present a short and efficient synthesis of useful 2-(4-dialkylaminophenyl)isoindoline electron-donating moieties (Scheme 2b), and their use in the Suzuki pathway as a convenient route to oxidation-sensitive acceptor–porphyrin–**TAPD** triads (Scheme 3). We also used this versatile approach to synthesize a triad with a **C_60_** acceptor moiety, which was predicted, and found, to have a longer-lived CSS than **TNQ–ZnP–TAPD**.

## Results and discussion

### Synthesis

**Scheme 4:**
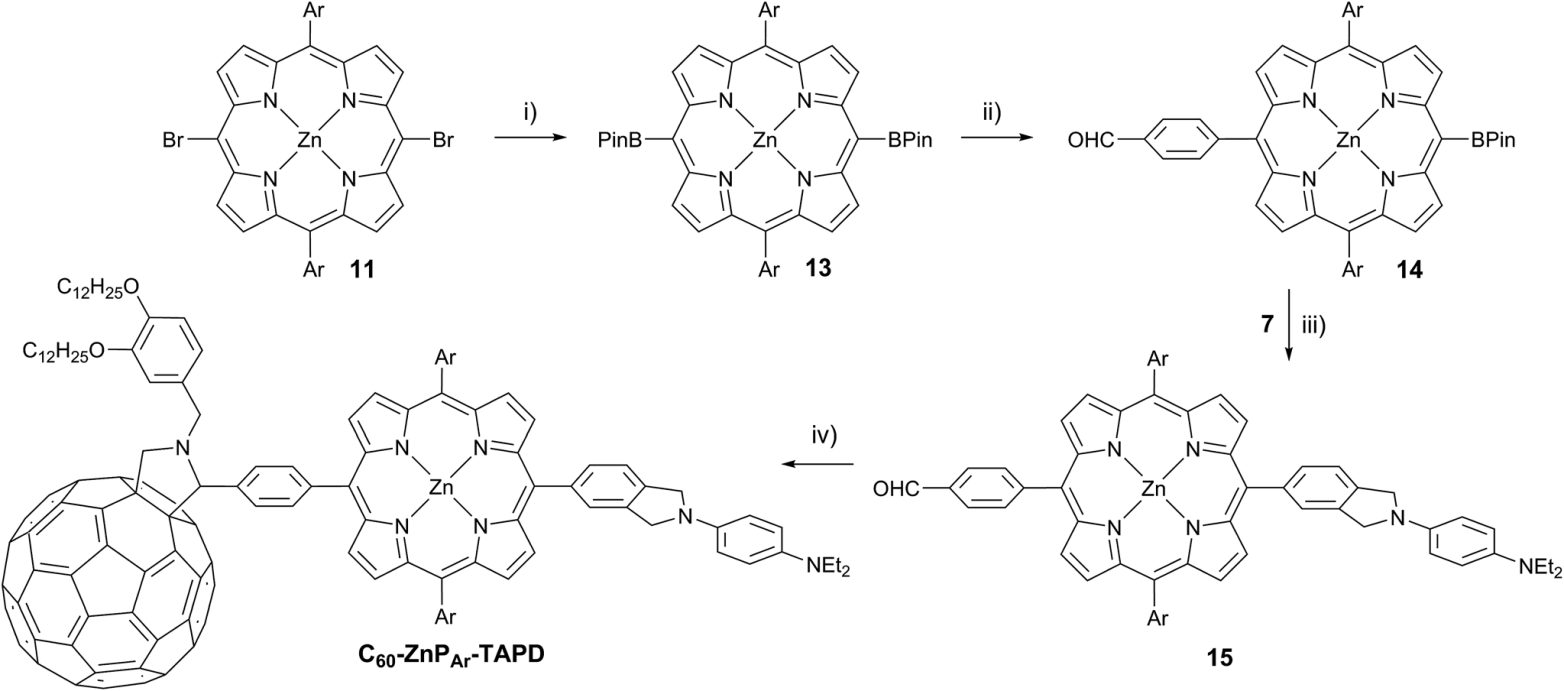
(i) HBPin, PdCl_2_(PPh_3_)_2_, NEt_3_, PhMe/THF, 24 h, 68 °C, 74%; (ii) 4-iodobenzaldehyde, K_2_CO_3_, Pd(PPh_3_)_4_, THF/H_2_O, 80 min, 66 °C, 35%; (iii) **7**, K_2_CO_3_, Pd(PPh_3_)_4_, THF/H_2_O, 4.5 h, 66 °C, 88%; (iv) 2-((3,4-bis(dodecyloxy)benzyl)amino)acetic acid, **C_60_**, PhMe, 2 h, 110 °C, 22%.

At the start of this project, we attempted to synthesize **TNQ–ZnP–TAPD** as reported by Wasielewski *et al.* (Scheme 1, Route A and Scheme 2a).^7^ We developed an efficient three-step synthesis of aldehyde **3** (Scheme 2b): first, 4-nitroaniline was condensed with 1,2,4-benzenetricarboxylic anhydride to yield **1**. The imide and carboxylic acid functions were then simultaneously reduced with borane to give **2** and this benzyl alcohol was re-oxidized using activated manganese dioxide to yield aldehyde **3**, in 50% over 3 steps. However, in our hands, the condensation of aldehydes **3** and **4** with tetraalkyl-dipyrromethane **5** did not give the desired porphyrin. Therefore, we decided to explore Suzuki coupling routes to the closely related triad **TNQ–ZnP_Ar_–TAPD** (Scheme 1, Route B, and Schemes 3 and 4).

The boronic ester substituted donor moiety was synthesized in good yield using the phthalimide route developed for the synthesis of aldehyde **3**: 4-(*N*,*N*-diethylamino)aniline was condensed with 4-bromophthalic anhydride in refluxing acetic acid to yield **6**, which was then reduced to the isoindoline using borane in refluxing tetrahydrofuran to obtain **7**. Its borylated equivalent **8** was subsequently obtained *via* palladium-catalyzed borylation in an overall 45% yield. The acceptor boronic ester **10** was synthesized (Scheme 3) from 2-bromoanthracene **9** (ref. 14) through Diels–Alder reaction with 1,4-naphthoquinone,^15^ and subsequent palladium-catalyzed borylation^16^ of the bromo-triptycenequinone.

The donor and acceptor moieties were linked to the central porphyrin core *via* a two-step Suzuki cross-coupling. First, the reaction of 3 equivalents of dibromo-porphyrin^11^**11** with 1 equivalent of the acceptor boronic ester **10** gave **12** in 69% yield, which was reacted in a second step with 1.1 equivalents of the donor boronic ester **8** to yield **TNQ–ZnP_Ar_–TAPD** in 72% yield. Performing the Suzuki couplings in two successive steps gave better yields and made purification easier than the simultaneous statistical one-pot coupling of both the donor and the acceptor to the porphyrin. To test the versatility of the Suzuki-based route to porphyrin triads, we synthesized another triad, **C_60_–ZnP_Ar_–TAPD** (Scheme 4), using the second Suzuki route (Scheme 1, Route C). When we performed the Suzuki coupling of **13** (ref. 17) with 4-iodobenzaldehyde, substantial deborylation of the porphyrin was observed. Therefore, the reaction was carried out with an excess of 4-iodobenzaldehyde and stopped as soon as formation of the bis(*p*-benzaldehyde)porphyrin adduct was detected by thin layer chromatography (TLC), to obtain **14** in 35% yield. Using an excess of **8**, in the second Suzuki coupling yielded **15** in 88%, which was then transformed into **C_60_–ZnP_Ar_–TAPD** using 2-((3,4-bis(dodecyloxy)benzyl)amino)acetic acid and **C_60_** in a fast Prato reaction.^18^

### Thermodynamics of electron transfer

**Fig. 2:**
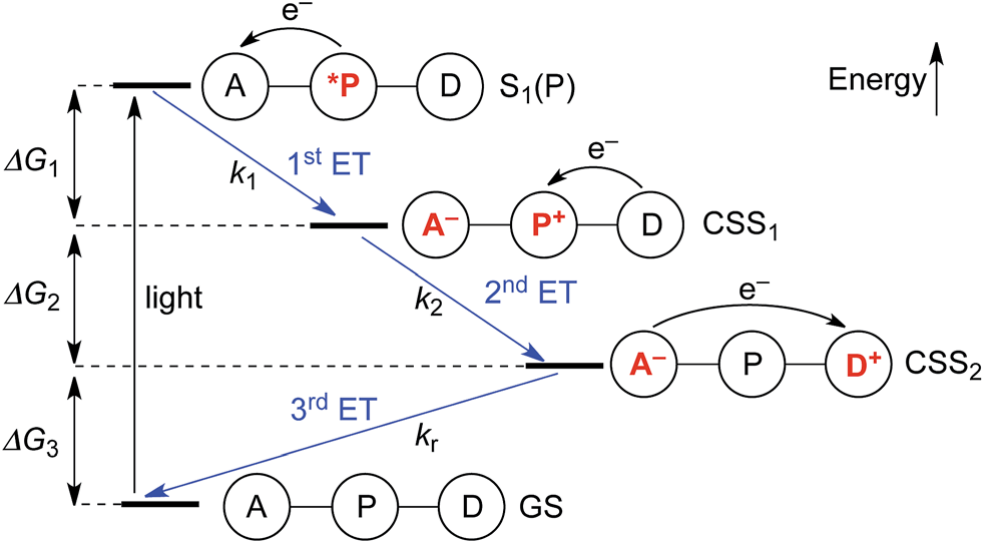
Electron transfer processes occurring upon photo-excitation of a triad. A = acceptor, P = zinc porphyrin, D = donor.

Upon photo-excitation, the triads can undergo three consecutive electron transfer (ET) processes: (1) ET from the excited porphyrin to the acceptor, (2) ET from the donor to the oxidized porphyrin and finally, (3) back-ET from the reduced acceptor to the oxidized donor (Fig. 2). Triad **TNQ–ZnP_Ar_–TAPD** is, by design, similar to **TNQ–ZnP–TAPD**, since it possesses the same donor and acceptor moieties, and only differs by the substitution on the porphyrin core. In order to evaluate the consequences of this different substitution pattern, and the effect of switching from a quinone to a fullerene acceptor in **C_60_–ZnP_Ar_–TAPD**, we estimated the energies of the first and second charge-separated states, both experimentally and computationally.

#### Electrochemical analysis

The change in free energy associated with the first electron transfer step, Δ*G*_1_, can be predicted, under solution-phase conditions, from the energy of the first singlet excited state S_1_ of the porphyrin, the oxidation potential of the porphyrin, *E*_ox_(P), and the reduction potential of the acceptor, *E*_red_(A), using the Rehm–Weller equation (eqn (2)),^19^2
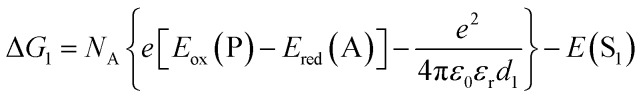
where *N*_A_ is the Avogadro constant, *e* is the elementary charge, *ε*_0_ is the vacuum permittivity, *ε*_r_ is the dielectric constant of the solvent (8.9 for dichloromethane) and *d*_1_ is the distance of charge separation in CSS_1_ (1.0 nm for all three triads). Similarly, the energy changes for the second and third electron transfer processes can be calculated from eqn (3) and (4):3
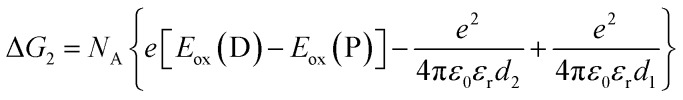

4
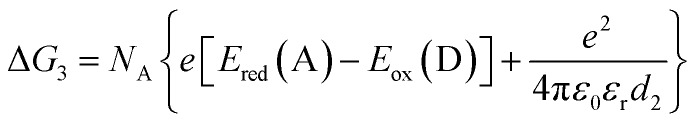
where *d*_2_ is the distance of charge separation in CSS_2_ (2.3 nm in **TNQ–X–TAPD** and 2.4 nm in **C_60_–ZnP–TAPD**, from molecular mechanics calculations).

**Fig. 3:**
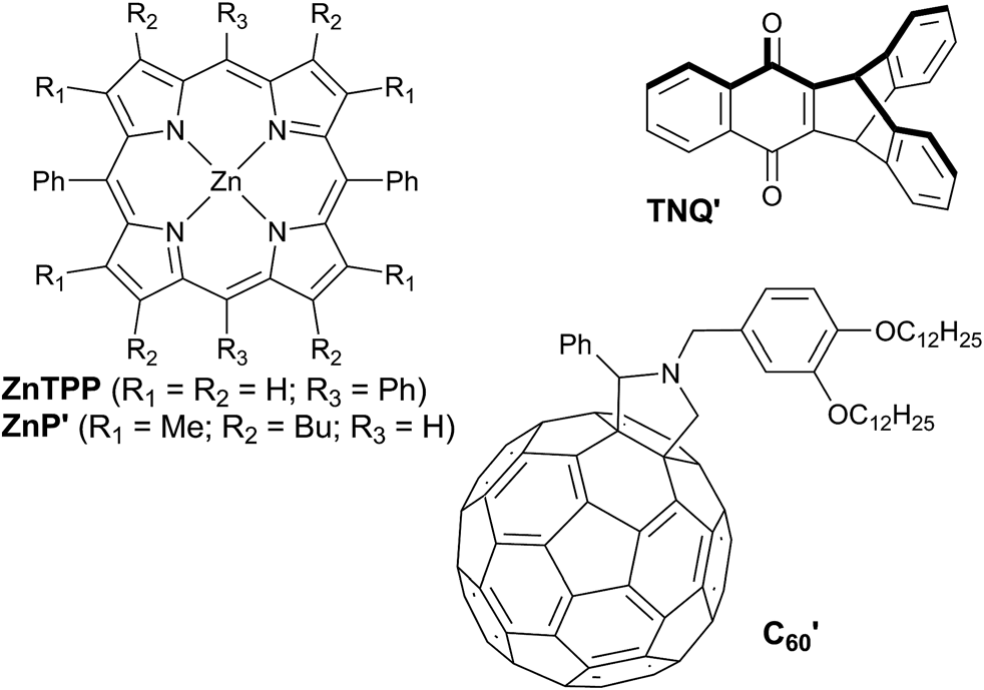
Reference compounds used to measure redox potentials.

The redox potentials *E*_ox_(D) and *E*_red_(A) were measured using squarewave voltammetry, and *E*(S_1_) values were estimated from absorption spectra (Table 1), using reference compounds **7** and those in Fig. 3 as models for the isolated donor, porphyrins and acceptor units. Electrochemical measurements on the complete triads gave very similar redox potentials to their isolated components, for example **C_60_–ZnP_Ar_–TAPD** shows oxidation waves at –0.29 V (**TAPD**) and 0.32 V (**ZnP_Ar_**) and reduction waves at –0.95 V (**C_60_**) and –1.84 V (**ZnP_Ar_**). The values of Δ*G* for electron transfer for steps 1–3 (Fig. 2) derived from the electrochemical potentials in Table 1 according to eqn (2)–(4) are listed in Table 2.

**Table 1:** Electrochemical and optical measurements

	**ZnP′**	**ZnP′_Ar_** (=**ZnTPP**)	**TAPD** (=**7**)	**TNQ′**	**C′_60_**
*E*_ox_ ^*a*^ (V)	0.17	0.32	–0.24	—	—
*E*_red_ ^*a*^ (V)	–2.13	–1.85	—	–1.15	–0.99
*λ*_max_ ^*b*^ (nm)	572	585	—	—	—
*E*(S_1_)^*c*^ (eV)	2.17	2.12	—	—	—

^*a*^Measured by squarewave voltammetry *vs*. Fc/Fc^+^ in dichloromethane with 0.1 M NBu_4_PF_6_ as electrolyte.

^*b*^ *λ*_max_ of the longest absorption band in dichloromethane.

^*c*^ *E*(S_1_) = *hc*/*λ*_max_. Structures of reference compounds **ZnTPP**, **ZnP′**, **C′_60_** and **TNQ′** are displayed in Fig. 3.

**Table 2:** Experimentally and theoretically derived free energies for each electron-transfer processes for the three triads (eV)

	**TNQ–ZnP–TAPD**	**TNQ–ZnP_Ar_–TAPD**	**C_60_–ZnP_Ar_–TAPD**
**Experimental, fluid solution** ^*a*^			
Δ*G*_1_	–1.01 (–0.86^*b*^)	–0.81^*a*^	–0.97^*a*^
Δ*G*_2_	–0.32^*a*^ (–0.45^*b*^)	–0.47^*a*^	–0.47^*a*^
Δ*G*_1_ + Δ*G*_2_	–1.33^*a*^ (–1.31^*b*^)	–1.28^*a*^	–1.44^*a*^
Δ*G*_3_	–0.84^*a*^ (–0.84^*b*^)	–0.84^*a*^	–0.68^*a*^

**Calculated, fluid solution** ^*c*^ ^,^ ^*d*^			
Δ*G*_1_	–1.08/–0.94/–1.07	–0.85/–0.78/–0.97	—^*e*^
Δ*G*_2_	–0.51/–0.63/–0.63	–0.68/–0.73/–0.68	—^*e*^
Δ*G*_1_ + Δ*G*_2_	–1.59/–1.57/–1.70	–1.53/–1.51/–1.65	–1.31/–1.44/–1.69
Δ*G*_3_	–0.79/–0.85/–0.72	–0.79/–0.85/–0.72	–1.01/–0.92/–0.75

**Calculated, frozen glass** ^*c*^			
Δ*G*_1_	–0.32/–0.18/–0.42	–0.04/+0.03/–0.32	—^*e*^
Δ*G*_2_	+0.11/–0.07/–0.01	–0.06/–0.17/–0.06	—^*e*^
Δ*G*_1_ + Δ*G*_2_	–0.21/–0.25/–0.43	–0.10/–0.15/–0.38	–0.06/–0.23/–0.46
Δ*G*_3_	–2.17/–2.17/–1.99	–2.17/–2.17/–1.99	–2.26/–2.13/–1.98

^*a*^Experimental values where calculated using eqn (2)–(4), from optical and electrochemical data determined in dichloromethane reported in Table 1.

^*b*^Values measured for the formerly reported triad in butyronitrile.7*b*

^*c*^Theoretical values are shown in the order CAM-B3LYP/M062X/B3LYP; these values were calculated using the 6-31G(d) basis set.

^*d*^PCM solvation model in butyronitrile, for the complete triads.

^*e*^Data not available due to problems with calculating the energy of CSS_1_.

For the first electron transfer in solution, Δ*G*_1_ is negative for all triads (–1.01 eV for **TNQ–ZnP–TAPD**, –0.81 eV for **TNQ–ZnP_Ar_–TAPD** and –0.97 eV for **C_60_–ZnP_Ar_–TAPD**). The second electron transfer step is also exergonic for all the triads because *E*_ox_(**TAPD**) ≪ *E*_ox_(**ZnP_Ar_**) < *E*_ox_(**ZnP**). The strong favorability of electron transfer is clear from the total free energies of electron transfer (Δ*G*_1_ + Δ*G*_2_). The aim of this project was to create triads that would give long-lived CSSs at low temperatures, in a frozen solvent glass, for EPR quantum information experiments. This makes the huge driving force for charge separation important, because it enables electron transfer to be favorable even at low temperatures, under the conditions of a frozen solvent glass, when solvent dipoles cannot reorient in response to the new charge distribution in CSS_1_ and CSS_2_, as discussed below. The third electron transfer, corresponding to the charge recombination, is also exergonic for all three triads because **TAPD** is not a strong enough electron donor to reduce the ground-state acceptors **TNQ** and **C_60_**.

#### Molecular geometries

The β-alkyl substituents in **TNQ–ZnP–TAPD** enforce a strictly orthogonal conformation between the porphyrin and the aryl substituents linking the donor/acceptor moieties, while this torsion is less constrained in the **X–ZnP_Ar_–TAPD** compounds.^20^ To estimate the resulting changes in dihedral angles between the porphyrin plane and the *meso*-linked benzenes plane, we performed a statistical analysis of *meso*-aryl zinc porphyrin crystal structures using the Cambridge Structural Database (CSD, ESI†). We analyzed 343 structures with β-alkyl substituents and 1032 structures without β-substituents. For β-unsubstituted porphyrins, the distribution of dihedral angles is quite broad; the population density peaks at 68° and is greater than 50% of this peak value in the range 90 ± 28°. On the other hand, when there is a CH_2_ at the β-position next to the *meso*-aryl group, the population density peaks at 90° and is greater than 50% of this peak value in the range 90 ± 4°.

**Fig. 4:**
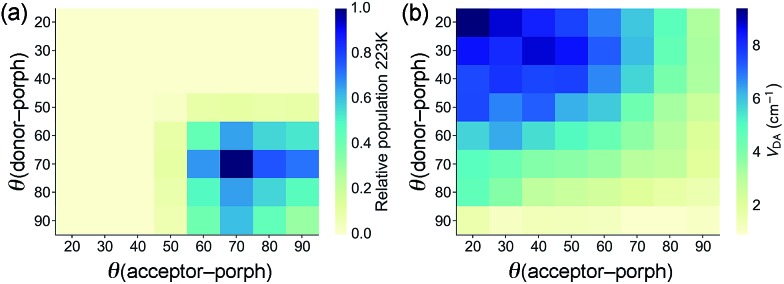
(a) Plots of the relative populations of conformations of **TNQ–ZnP_Ar_–TAPD** from the Boltzmann distribution at 223 K (the freezing point of xylene), and (b) the relative magnitude of the donor–acceptor electronic coupling *V*_DA_(GMH) as a function of the dihedral angles.

The geometries of all three triads were calculated at the B3LYP/6-31G(d) level. Calculated dihedral angles between the porphyrin and the donor unit, as well as between the porphyrin and the acceptor moiety (Table 3) agree well with the distributions from our CSD analysis. The less orthogonal geometries in **ZnP_Ar_**, compared to **ZnP**, lead to stronger electronic coupling, as discussed below. The Boltzmann distribution of dihedral angles in **TNQ–ZnP_Ar_–TAPD** at the temperature relevant to our EPR studies (223 K, the freezing point of xylene) is presented in Fig. 4a.

**Table 3:** Angle between the mean plane of the benzene ring in donor and acceptor and the mean plane of the porphyrin^*a*^

	*θ* (donor–porph)	*θ* (acceptor–porph)
**TNQ–ZnP–TAPD**	82°	86°
**TNQ–ZnP_Ar_–TAPD**	70°	68°
**C_60_–ZnP_Ar_–TAPD**	68°	71°

^*a*^B3LYP/6-31G(d) equilibrium geometry.

#### Calculated energy levels

**Fig. 5:**
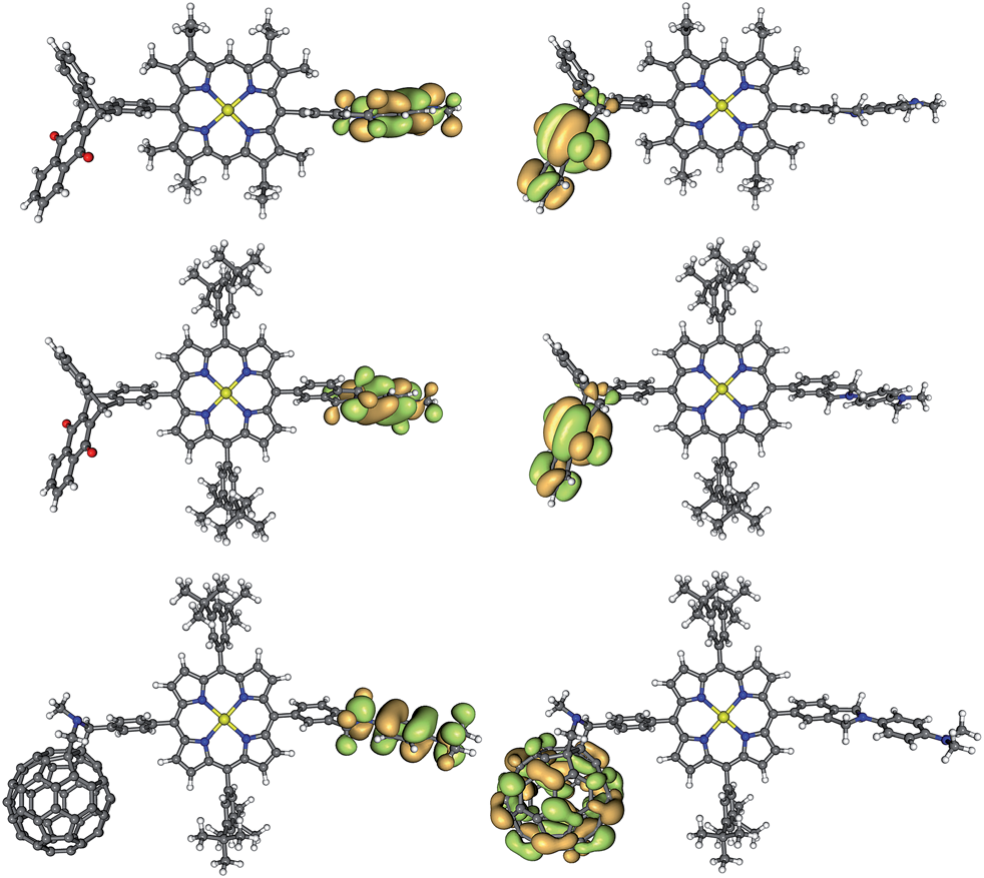
Frontier orbitals shapes (HOMO on the left, LUMO on the right) of **TNQ–ZnP–TAPD** (top), **TNQ–ZnP_Ar_–TAPD** (middle) and **C_60_–ZnP_Ar_–TAPD** (bottom) (B3LYP/6-31G(d)).

Frontier molecular orbital distributions for geometries optimized at the B3LYP/6-31G(d) level are shown in Fig. 5 (for HOMO–2 to LUMO+2, see ESI†). Despite the differences in the porphyrin-aryl dihedral angles, the shapes of the frontier orbitals are not significantly affected and the HOMO and LUMO are strongly localized on the respective donor and acceptor moieties. The sp^3^ carbons present in the porphyrin–donor and porphyrin–acceptor bridges act as insulators, regardless of the conformation, confirming that the design of **TNQ–ZnP_Ar_–TAPD** and **C_60_–ZnP_Ar_–TAPD** remains valid for stabilizing a long-lived CSS.

The calculated donor (**TAPD**) and acceptor (**TNQ** and **C_60_**) orbital energies (HOMO and LUMO) show little variation between the three compounds (Table 4, see ESI† for other levels of theory). However, the tetraaryl porphyrin core **ZnP_Ar_** has a smaller gap (2.84 *vs.* 2.97 eV; HOMO–1 to LUMO+1) and is a somewhat weaker electron donor (lower HOMO–1) than alkyl **ZnP** (–4.92 *vs.* –4.79 eV), due to the lack of electron-donating alkyl groups. TD-DFT calculations confirm a 0.05/0.06 eV lower optical gap, with S_1_ at 2.37/2.32 eV in **ZnP_Ar_** compared to 2.42/2.38 eV for alkyl **ZnP** at B3LYP/6-31G(d) and CAM-B3LYP/6-31G(d), respectively. Ionization potential calculations (IP, see below and ESI†) suggest a 0.05–0.20 eV difference in oxidation potential. This is in good agreement with the 0.15 eV difference in oxidation potential between the two **ZnP** and **ZnP_Ar_** porphyrins as measured by squarewave voltammetry, and the 0.05 eV difference in absorption energy from UV-visible spectroscopy (Table 1).

**Table 4:** B3LYP/6-31G(d) orbital energy levels^*a*^ (eV)

	**TNQ–ZnP–TAPD**	**TNQ–ZnP_Ar_–TAPD**	**C_60_–ZnP_Ar_–TAPD**
LUMO+2 (P)	–1.80	–2.05	–2.09
LUMO+1 (P)	–1.82	–2.08	–2.10
LUMO (A)	–3.02	–3.07	–3.10; –2.99; –2.75^*b*^
HOMO (D)	–4.35	–4.25	–4.27
HOMO–1 (P)	–4.79	–4.92	–4.95
HOMO–2 (P)	–4.90	–5.08	–5.12

^*a*^P, A and D indicate the location of the orbital on the porphyrin, acceptor or donor.

^*b*^The LUMO of **C_60_** is triply degenerate, but the saturation at the pyrrolidine linking breaks the symmetry.

The reaction free energies Δ*G*_1_, Δ*G*_2_ and Δ*G*_3_ were estimated for solution-phase ET using DFT. Since the singlet–triplet gap for long-range charge-transfer systems such as these is negligible, we modeled the CSSs as the lowest unrestricted DFT triplet. The effect of the solvent was included using the Integral Equation Formalism Polarizable Continuum Model (IEFPCM) as implemented in Gaussian 09 with Universal Force Field (UFF) radii and default parameters. Energies calculated using the SMD model^21^ were within 0.05 eV of these values. Calculations in liquid solution were carried out using *ε*_r_, the static (or zero-frequency) dielectric constant of the solvent, which includes the effect of electronic and dipolar polarization. Given their small effect in the donor–acceptor charge transfer energies, the bis-3,5-*tert*-butylphenyl side groups in **ZnP_Ar_** were substituted by hydrogen atoms to reduce the number of nuclear and electronic degrees of freedom when the calculations did not involve states located on the porphyrin.

The results of these calculations, using three different computational methods (CAM-B3LYP/M062X/B3LYP), are compared with Δ*G* values from electrochemical measurements in Table 2. The calculated free energies confirm that the intermediate CSS_1_ is systematically shifted up in energy by the structural modification in **ZnP** → **ZnP_Ar_**, making Δ*G*_1_ less exergonic and Δ*G*_2_ more exergonic.

Our UDFT calculations for the CSS_1_**C_60_^–^–ZnP_Ar_^+^–TAPD** did not converge to the diradical state, but rather to a localized triplet, preventing calculation of Δ*G*_1_ and Δ*G*_2_. However for **C_60_–ZnP_Ar_–TAPD**, we can still calculate the total free energy change for electron transfer (Δ*G*_1_ + Δ*G*_2_), as shown in Table 2. All DFT functions tested incorrectly predicted **TNQ** to be a more powerful acceptor in solution than **C_60_** (between 0.15 to 0.05 eV, with both the 6-31G(d) and 6-311G(d,p)). In vacuum, however, this trend was reversed and the correct behavior was recovered, with a difference of 0.2–0.4 eV, as is the case when the effect of the frozen solvent is taken into account (see below). This discrepancy may be attributed to the failure of continuum solvent models to treat cavitation–dispersion interactions.

#### Electron transfer in frozen solvents

Below the freezing point, the solvent molecules cannot reorient their dipoles in response to local changes in charge of the solute. This lack of dipolar polarization is equivalent to the outer-sphere reorganization energy for electron-transfer reactions in solution, where the reorientation of the solvent molecules is much slower than the response time of their electron clouds. We modeled this lack of dipolar polarization by performing non-equilibrium IEFPCM calculation. When including the frozen solvent effect in butyronitrile (optical dielectric constant: *ε*_∞_ = 1.9) we obtained the free energies for electron transfer in the frozen solvent glass shown in Table 1. In **TNQ–ZnP–TAPD** and **TNQ–ZnP_Ar_–TAPD**, the first and second charge separated states, CSS_1_ and CSS_2_, are shifted up in energy by about 0.75 and 1.32 eV, respectively, which makes charge-separation scarcely favorable. If we compare this increase in the energy of CSS_2_ (1.32 eV) with the experimental values of Δ*G*_1_ + Δ*G*_2_ in **TNQ–ZnP–TAPD** and **TNQ–ZnP_Ar_–TAPD** (–1.33 and –1.28 eV, respectively), it is evident that there is almost no driving force for charge separation in the frozen state. In **C_60_–ZnP_Ar_–TAPD**, the energy of CSS_2_ is increased by about 1.23 eV, which is slightly less than in the other triads, and the experimental value of Δ*G*_1_ + Δ*G*_2_ in is slight more negative (–1.44 eV) so charge-separation is expected to be more exergonic.

These calculations were carried out for butyronitrile as the solvent, for consistency with earlier studies by Wasielewski and co-workers,^7^ however other solvents have quite similar optical dielectric constants (*ε*_∞_ ≈ *n*^2^, where *n* is the refractive index) so that the free energies changes are expected to be similar in other frozen solvents.

### Calculated rates of charge recombination

Rates of electron transfer are governed by a combination of the reaction thermodynamics (Δ*G*), the stiffness of the potential energy surface (*λ*) and the coupling of the initial and final states (*V*_DA_), as discussed above (eqn (1)).

The electronic coupling between the ground state and the CSS_2_ (HOMO → LUMO excitation) was estimated using two-state approximation schemes: the Generalized Mulliken–Hush (GMH)^22^ and the Fragment-Charge Difference^23^ (FCD) methods. Both approaches produced very similar *V*_DA_ values for the three compounds, with **TNQ–ZnP_Ar_–TAPD** showing the largest coupling in the series (Table 5).

**Table 5:** Electronic couplings (*V*_DA_) and inner-sphere reorganization energies (*λ*) for CSS_2_ from TD-DFT B3LYP/6-31G(d) calculations

	**TNQ–ZnP–TAPD**	**TNQ–ZnP_Ar_–TAPD**	**C_60_–ZnP_Ar_–TAPD**
*V*_DA_(FCD) (cm^–1^)	0.23	2.6	0.27
*V*_DA_(GMH) (cm^–1^)	0.30	3.0	0.31
*λ* (eV)	0.44	0.44	0.30

The electronic couplings listed in Table 5 were calculated by considering only the lowest energy conformation of each molecule. In the case of **TNQ–ZnP_Ar_–TAPD**, we also calculated the coupling *V*_DA_(GMH) as a function of the dihedral angles to the donor and acceptor (Fig. 4b). This plot, together with the Boltzmann distribution of dihedral angles (Fig. 4a), shows that the range of conformations populated in frozen xylene (*θ* ≈ 90 ± 30°) show modest variation in coupling (*V*_DA_ ≈ 3 ± 2 cm^–1^). The energy Δ*G*_3_ of the CSS_2_ of **TNQ–ZnP_Ar_–TAPD** is also insensitive to the dihedral angle (*ca.* 1% variation for *θ* = 90 ± 30°, see ESI Fig. S2†) which indicates that it is reasonable to consider only the lowest energy conformation of this molecule.

The treatment of low-frequency, thermally accessible vibrational modes in Marcus theory is fairly straightforward *via* a harmonic potential with a recombination energy. However, in the low-temperature regime where reactions progress almost exclusively through tunneling, electron-vibration coupling must be treated more explicitly. We modeled vibronic coupling under the Franck–Condon approximation by determining the Huang–Rhys factors (*S*) for the electron transfer.^24^ We used several approaches to estimate *S* (see ESI† for details), using both the ground state and CSS_2_ vibrational modes. The results from these two methods were consistent with each other. At each level of theory, there is one *S*_*q*_ for each vibrational mode *q*, of frequency *ω*_*q*_. For ease of comparison, these can be rolled into classical reorganization energies 
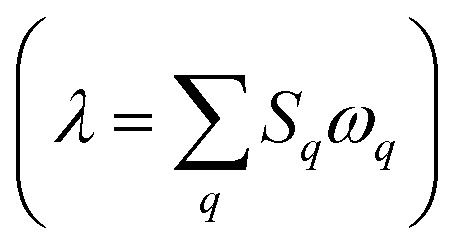
. Table 5 reports vibrational (inner-sphere) reorganization energy for the two donor–acceptor combinations. The system with a **C_60_** acceptor has a lower reorganization energy by around 0.15 eV, in keeping with the known low *λ* of fullerenes in general.^25^

Electron transfer rates (*k*_r_, Fig. 2), in the low-temperature-limit, were estimated using eqn (5)5
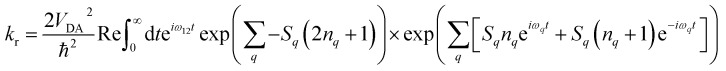
which is derived from the molecular crystal model.^26^ It combines the parameters described above and can be solved approximately using the steepest-descent method, in combination with a saddle-point time chosen for optimally sharing the exoergicity among the vibrational modes; *ω*_12_ is the frequency corresponding to Δ*G*_3_, *S*_*q*_ is the Huang–Rhys factor associated to vibrational mode *q* of frequency *ω*_*q*_, and *n*_*q*_ = (e^*ℏω*_*q*_/*kT*^ – 1)^–1^ is the Bose Einstein occupation factor for mode *q*.

Combining the different levels of theory (CAM-B3LYP/6-31G(d), M062X/6-31G(d) and B3LYP/6-31G(d)) and the various estimates of *S* we obtained a range of values for these rates in frozen butyronitrile at 4 K. Very little temperature dependence of the results was observed in the 0–10 K regime. The predicted recombination lifetimes of **TNQ–ZnP–TAPD** range between 0.2 and 6.2 ms, with a geometric mean of 1.6 ms, in remarkable agreement with the experimentally reported 4 ms. Since the only difference between **TNQ–ZnP–TAPD** and **TNQ–ZnP_Ar_–TAPD** in our models is *V*_DA_ (increased by a factor of 10, averaging between GMH and FCD) we predicted a lifetime 0.02 ms for **TNQ–ZnP_Ar_–TAPD**. Since **C_60_–ZnP_Ar_–TAPD** has *V*_DA_ close to **TNQ–ZnP–TAPD**, but a much lower reorganization energy, the vibronic coupling between initial and final state is lower. Thus, using eqn (5) we estimated a much longer lifetime (geometric mean prediction 260 ms, range between 6 and 2700 ms).

### Experimental characterization of CSS_2_ by EPR

**Fig. 6:**
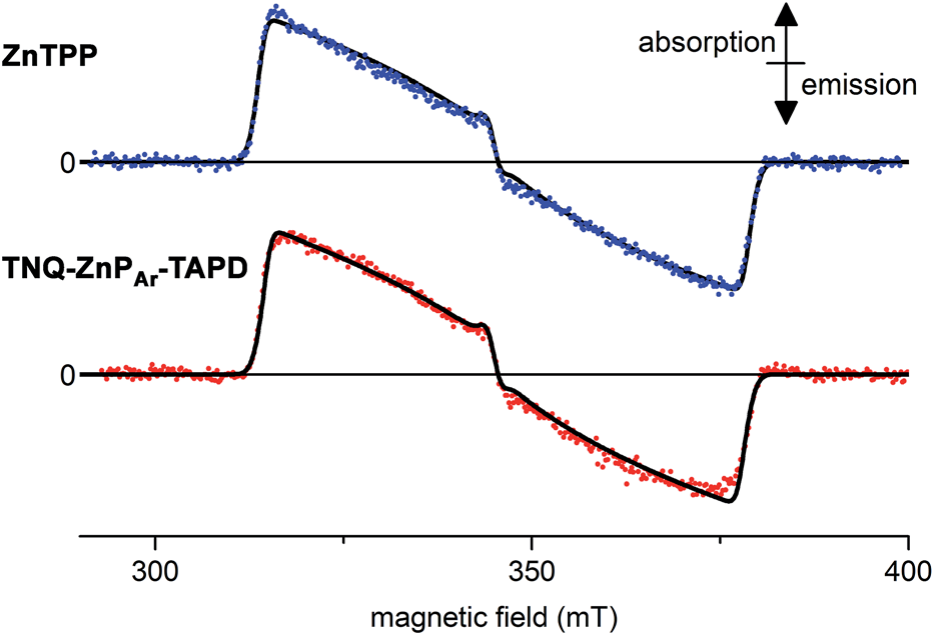
Experimental (dots) and simulated (lines, see ESI† for parameters) X-band time-resolved continuous wave EPR spectra of photo-excited **ZnTPP** (top) and **TNQ–ZnP_Ar_–TAPD** (bottom). Concentration: 100 μM in xylenes; temperature: 10 K; excitation: 10 mJ monochromatic 20 Hz laser (590 nm); spectra recorded 1.0 μs after the laser flash.

We measured the time-resolved EPR spectrum of the photo-excited triad **TNQ–ZnP_Ar_–TAPD** under similar conditions to those reported by Wasielewski and co-workers in three different solvents (butyronitrile, 2-methyltetrahydrofuran and xylenes). Disappointingly, we were not able to detect any trace of a long-lived photo-excited charge-separate state. Instead we only detected the signal of the porphyrin triplet excited state. This was confirmed by comparing with zinc tetraphenylporphyrin in the same solvent and concentration, which gave an identical transient EPR spectrum of the zinc-porphyrin triplet state (Fig. 6). The failure to detect a long-lived CSS for **TNQ–ZnP_Ar_–TAPD**, whereas one was observed for **TNQ–ZnP–TAPD**, can be explained by the greater electronic coupling, *V*_DA_, which arises from the orthogonal dihedral angle between the porphyrin unit and the benzene rings linking the donor and the acceptor (Fig. 4b). The slight differences in the thermodynamics of electron transfer between these molecules, also makes charge separation less favorable in **TNQ–ZnP_Ar_–TAPD** (Table 2). The singlet excited state of the tetraaryl porphyrin (**ZnP_Ar_**) is lower than that of the diaryl porphyrin (**ZnP**) by about 0.05 eV which slightly reduces the total driving force (Δ*G*_1_ + Δ*G*_2_) for formation of CSS_2_ in **TNQ–ZnP_Ar_–TAPD**. However the main difference between these two systems is probably the lower oxidation potential of **ZnP_Ar_** which makes Δ*G*_1_ less favorable for charge separation. This subtle change in thermodynamics appears to be enough to prevent charge separation in a frozen solvent matrix.

**Fig. 7:**
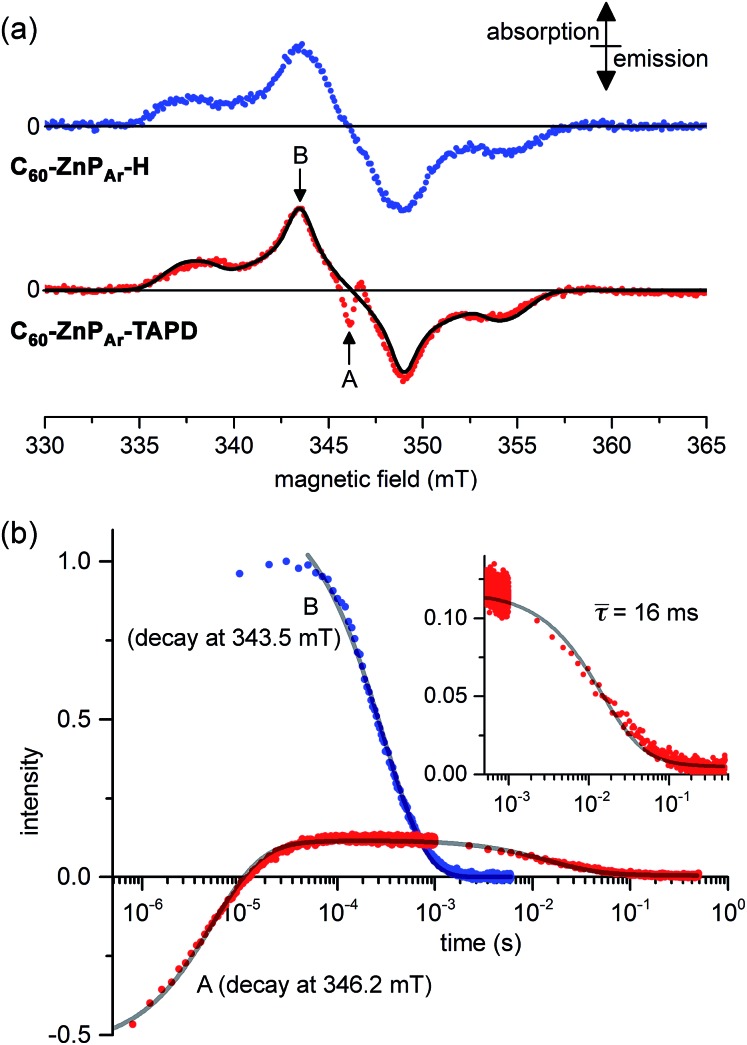
(a) X-band transient continuous-wave EPR spectra of photo-excited **C_60_–ZnP_Ar_–H** (top) and **C_60_–ZnP_Ar_–TAPD** (bottom); experimental (dots) and simulated ^3^**C_60_** triplet spectrum (line). (b) X-band pulsed-EPR flash delay experiment performed on **C_60_–ZnP_Ar_–TAPD**. (A) Field: 346.2 mT (radical pair signal). (B): Field: 343.5 mT (^3^**C_60_** signal); both decays were fitted using a kinetic model described in the ESI.† The insert is the later part of the decay for (A), with the mean decay time, *τ* = (64)^–1^ s. 100 μM in xylenes at 10 K. Excitation: 10 mJ, 1 Hz, 590 nm; spectra recorded 1.2 μs after the laser flash. (A and B) are the fields indicated in part (a).

Field-sweep photo-EPR experiments on **C_60_–ZnP_Ar_–TAPD** showed the expected signal of the spin-polarized long-lived CSS_2_ (Fig. 7a, bottom, central emission/absorption feature (A)) on the top of a polarized ^3^**C_60_** triplet spectrum. The spectrum of the reference compound **C_60_–ZnP_Ar_–H**, recorded under identical conditions (Fig. 7a, top), shows only the ^3^**C_60_** triplet signal (B). The CSS_2_ signal was observed for solutions of **C_60_–ZnP_Ar_–TAPD** in xylene and in 2-methyltetrahydrofuran (but could not be investigated in butyronitrile due to limited solubility).
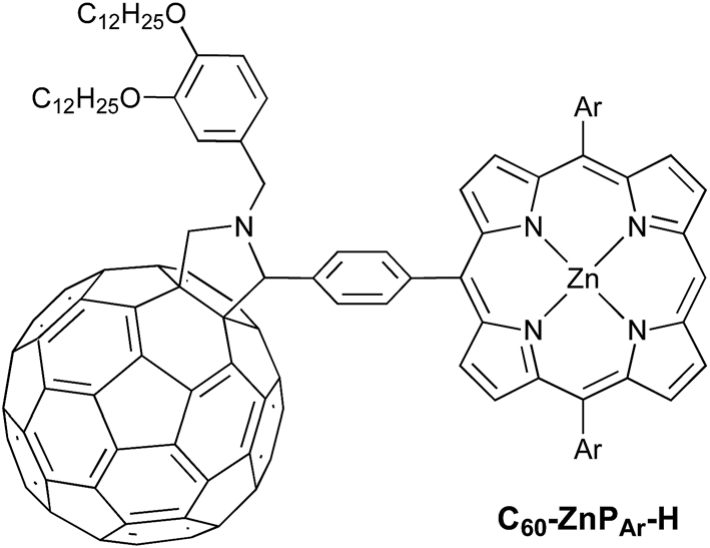


We performed pulsed EPR experiments in which the integrated Hahn-echo intensity at a particular magnetic field position in the spectrum was recorded as a function of the time after laser excitation, to explore the time-evolution of the transient species probed at the chosen field position. These echo-integrated flash delay experiments were carried out at two different field positions corresponding to the fullerene triplet state (Fig. 7b, curve B) and the radical pair state (Fig. 7b, curve A) of **C_60_–ZnP_Ar_–TAPD**. We were able to simulate both decay curves using a kinetic model, considering the ^3^**C_60_** triplet, the singlet CSS_2_ and the three sub-levels, T_0_, T_+_ and T_–_ of the triplet CSS_2_ (see ESI†). The ^3^**C_60_** signal (curve B) follows a mono-exponential decay, with a lifetime of 300 ± 3 μs, which is consistent with previous reports.^27^ On the other hand, the radical pair signal (curve A) displays two different decay processes. In the μs regime, the emission/absorption signal becomes inverted to an absorption/emission pattern. This is attributed to the spin-allowed decay of the S–T_0_ sublevels of the radical pair. We fitted this decay to a Gaussian distribution of rate constants (mean: 1.8 × 10^5^ s^–1^; standard deviation: 0.7 × 10^5^ s^–1^) corresponding to a mean lifetime of 5.6 μs.

In the ms regime, we observe the decay of a positive signal (Fig. 7b, curve A and insert), corresponding to decay of the remaining T_+_–T_–_ sublevel population of the CSS_2_ radical pair. This decay process was modeled with a Gaussian distribution of rate constants (mean: 64 ± 6 s^–1^; standard deviation: 32 s^–1^). This distribution of rate constants for both decay processes, from S–T_0_ and T_+_–T_–_ sublevels, probably reflects the spread of molecular conformation in the frozen solution. The mean lifetime of the charge-separate state of 16 ms is among the longest reported in the literature, and is about four times longer than for **TNQ–ZnP–TAPD**,^1^,^7^ in keeping with the trend predicted by our computational studies.

Comparison of the integrated intensity of the EPR signal from CSS_2_ with that of the **C_60_** triplet, at early times after excitation, indicates that the quantum yield of formation of CSS_2_ in **C_60_–ZnP_Ar_–TAPD** is about 0.1. This is a rough estimate, based on the assumptions that formation of CSS_2_ and the **C_60_** triplet are the dominant decay channels, and that these species have similar polarizations. It is difficult to accurately integrate the CSS_2_ signal as it has overlapping emissive and absorptive bands (see ESI† for details).

## Conclusions

In this study, we have reported a versatile and convergent route to donor–zinc tetraphenylporphyrin–acceptor triads *via* Suzuki cross coupling reactions, which is compatible with oxidation-sensitive moieties. This approach was applied to synthesize two tetralkylphenylenediamine/zinc porphyrin/acceptor triads. We explored the kinetics and thermodynamics of charge separation in both systems. Triads exhibiting long-lived CSSs at low temperatures in frozen solvents are needed for experiments in the area of quantum information processing, yet it is difficult to achieve charge-separation under these conditions, because the frozen solvent molecules cannot reorient to stabilize the new charge distribution. For the first triad, **TNQ–ZnP_Ar_–TAPD**, our calculations indicated that there would be almost no thermodynamic driving force for charge-separation in a frozen solvent. In keeping with this prediction, we were unable to detect a CSS in this system by EPR. For the second triad, **C_60_–ZnP_Ar_–TAPD**, the greater electron-affinity of **C_60_** was expected to make electron transfer favorable, while the weak coupling between the donor and the acceptor, *V*_DA_, and the small reorganization energy, *λ*, were predicted to result in an exceptionally long-lived CSS. These predictions were confirmed by the observation of a long-lived CSS in the solid state by EPR spectroscopy. The characteristic lifetime of the triplet CSS of this system is 16 ms, in xylenes at 10 K, which is among the longest reported.^1^ Changing the acceptor from **TNQ** to **C_60_** has three important consequences: (1) it increases the driving force for electron transfer, making charge-separation favorable, even in a frozen solvent, (2) it reduces the coupling term *V*_DA_, resulting in a slow rate of charge recombination, and (3) it reduces the reorganization energy, *λ*_i_, also contributing towards a slow recombination rate. Further spectroscopic studies on triad **C_60_–ZnP_Ar_–TAPD** are in progress, and will be described in a future report.

This work illustrates the value of quantum mechanical modeling for guiding the synthesis of electron-transfer systems. It also demonstrates the power of Suzuki coupling methodology, using a porphyrin core with either bromine or boronic acid substituents, for building triads with sensitive donor groups. The strategy developed here should provide a route to preparing advanced molecular materials with long photoexcited CSS lifetimes, for applications such as photo-voltaic devices, and optically gated molecular wires.^28^

## Experimental section

### General information

All chemical reagents were used as received. 2-Bromoanthracene^14^ and [5,15-bis-(3,5-bis-*tert*-butylphenyl)-10,20-bisbromoporphinato]zinc(ii)^13^ were synthesized following a literature procedures. Dichloromethane (DCM) and tetrahydrofuran (THF) were dried over activated alumina prior to use. Anhydrous *N*,*N*-dimethylformamide (DMF), acetic acid, nitrobenzene, pyridine, toluene, xylenes and anhydrous 2-methyltetrahydrofuran (MTHF) were supplied by Aldrich and used without further purification. Purge gas was high purity argon. Chromatography was performed on silica (200–400 mesh). ^1^H NMR spectra were acquired on a 400 MHz (Bruker AVII 400), 500 MHz (Bruker AVII 500) or 700 MHz (Bruker AVIII 700) spectrometer. Chemical shifts (in the ppm scale) were determined *versus* TMS using the residual solvent peak as the internal reference (CHCl_3_, *δ* = 7.26 ppm). The ^1^H NMR spectra of the final triads, **TNQ–ZnP_Ar_–TAPD** and **C_60_–ZnP_Ar_–TAPD** were fully assigned by comparison with the spectra of reference compounds, in combination with 2D techniques (COSY and HSQC); see ESI.† Deuterated chloroform was stored over potassium carbonate to avoid any acid trace. UV/Vis absorption spectra were recorded using a Perkin Elmer Lambda 20 UV-Vis Spectrometer. The absorption wavelengths are reported in nm with the extinction coefficient in M^–1^ cm^–1^. Infra-red spectra were recorded in the solid state (neat) using a Bruker Tensor27 FT-IR spectrometer. Mass spectroscopy was performed either on ESI-TOF (Waters LCT Premier) or MALDI-TOF (Waters MALDI Micro MX) spectrometer or using the Bruker Ultraflextreme MALDI-TOF/TOF spectrometer from the EPSRC National Mass Spectrometry Service (Swansea). Preparative scale size exclusion chromatography (SEC) was carried out using BioRad Bio-Beads S-X1 with toluene as eluent. ESR samples were prepared in 3.8 mm quartz tubes, sealed under vacuum and kept at 77 K in the dark.

### Computational details

DFT calculations were performed with Gaussian 09.^29^ The B3LYP, CAM-B3LYP and M062X functionals were used, in conjunction with the 6-31G(d) and 6-311+G(d,p) basis sets. Minima were confirmed by harmonic analysis. Atomic displacements were projected onto vibrational modes using the FCHT keyword. Solvent effects were included *via* the IEFPCM formalism, using default radii (butyronitrile, *ε*_r_ = 24.3, *ε*_∞_ = 1.9). Electronic coupling calculations were carried out using QChem 4.2.^30^

### Synthetic procedures

#### 2-(4-Nitrophenyl)isoindoline-1,3-dione-5-benzoic acid (**1**)

4-Nitroaniline (3.30 g; 23.7 mmol; 1 eq.) and 1,2,4-benzenetricarboxylic anhydride (5.00 g; 26.0 mmol; 1.1 eq.) were added to acetic acid (300 mL). The suspension was heated to reflux for 20 h, and then poured onto of ice (500 g). The solution was warmed to room temperature and the precipitate was filtered off and washed several time with water to yield **1** after drying under vacuum (7.00 g; 22.4 mmol; 95%) as a white powder. Analytical data: ^1^H NMR (400 MHz, DMSO-*d*_6_ + H_2_O): *δ*_H_ = 15.0 to 11.0 (s, 1H), 8.44 (dd, 7.6 Hz, 1.6 Hz, 1H), 8.42 (d, 9.2 Hz, 2H), 8.34 (d, 1.6 Hz, 1H), 8.13 (d, 7.6 Hz, 1H), 7.80 (d, 9.2 Hz, 2H) ppm; ^13^C NMR (100 MHz, CDCl_3_): *δ*_C_ = 165.8, 165.7 (2 carbons), 146.3, 137.6, 136.7, 135.8, 134.8, 132.0, 127.7, 124.3, 124.1, 123.7 ppm; HRMS (ESI): calculated for [M + Na]^+^ C_15_H_8_N_2_NaO_6_^+^: *m*/*z* = 335.0275; found *m*/*z* = 335.0271.

#### (2-(4-Nitrophenyl)isoindolin-5-yl)methanol (**2**)

A suspension of **1** (0.50 g; 1.6 mmol; 1 eq.) in dry THF (30 mL) was stirred under argon. A solution of borane in THF (1.0 M; 20.0 mL; 20.0 mmol; 12.5 eq.) was then added at 0 °C and the mixture was refluxed overnight. The mixture was cooled to 0 °C and water (100 mL), followed by an aqueous hydrochloric acid solution (2 M; 20 mL) were then added and **2** was extracted with DCM (3 × 250 mL). The organic phase was dried over MgSO_4_, filtered and evaporated. The product was then purified by chromatography (SiO_2_; eluent: THF 1/4 DCM) to yield **2** as a brown-yellow powder (0.325 g; 75%). Analytical data: ^1^H NMR (400 MHz, DMSO-*d*_6_ + H_2_O): *δ*_H_ = 8.15 (d, 8.8 Hz, 2H), 7.36 (m, 2H), 7.28 (d, 7.9 Hz, 1H), 6.77 (d, 8.8 Hz, 2H), 5.25 (s br, 1H), 4.78 (s, 4H), 4.53 (s, 2H) ppm; ^13^C NMR (100 MHz, CDCl_3_): *δ*_C_ = 151.6, 142.1, 136.4, 136.2, 134.8, 126.0, 125.8, 122.3, 120.7, 111.1, 62.7, 53.6, 53.5 ppm; HRMS (ESI): calculated for [M + Na]^+^ C_15_H_14_N_2_NaO_3_^+^: *m*/*z* = 293.0897; found *m*/*z* = 293.0897.

#### (2-(4-Nitrophenyl)isoindoline-5-carbaldheyde) (**3**)

To a solution of **2** (100 mg; 0.37 mmol) in chloroform (20 mL) was added manganese(iv) oxide (500 mg, 4.8 mmol). The mixture was stirred for 15 min at 25 °C and filtered. The solvent was evaporated and the product was purified by chromatography (SiO_2_; eluent: chloroform) to yield **3** as a yellow powder (70 mg, 0.26 mmol, 70%). Analytical data: ^1^H NMR (400 MHz, CDCl_3_): *δ*_H_ = 10.05 (s, 1H), 8.22 (d, 8.8 Hz, 2H), 7.88 (m, 2H), 7.54 (d, 7.9 Hz, 1H), 6.65 (d, 8.8 Hz, 2H), 4.85 (s, 4H) ppm; ^13^C NMR (100 MHz, CDCl_3_): *δ*_C_ = 191.5, 143.2, 151.0, 137.9, 137.7, 136.5, 130.3, 126.4, 123.5, 123.4, 110.7, 53.9, 53.6 ppm; HRMS (ESI): calculated for [M + Na]^+^ C_15_H_12_N_2_NaO_3_^+^: *m*/*z* = 291.0740; found *m*/*z* = 291.0731.

#### 5-Bromo-2-(4-(diethylamino)phenyl)isoindoline-1,3-dione (**6**)

4-(*N*,*N*-Diethylamino)aniline (0.720 g; 4.41 mmol; 1.0 eq.) and 4-bromophthalic anhydride (1.00 g; 4.41 mmol; 1 eq.) were added to acetic acid (25 mL). The suspension was heated to reflux for 20 h, and then poured onto ice (150 g). The product was extracted with DCM (3 × 50 mL) and the organic layer was washed several times with water, dried over MgSO_4_, filtered and evaporated to yield after filtration over SiO_2_ (eluent: DCM) **6** (1.56 g; 4.18 mmol; 95%) as an orange powder. Analytical data: ^1^H NMR (400 MHz, CDCl_3_): *δ*_H_ = 8.05 (s, 1H), 7.89 (d, 8.0 Hz, 1H), 7.78 (d, 8.0 Hz, 1H), 7.18 (d, 9.2 Hz, 2H), 6.73 (d, 9.2 Hz, 2H), 3.38 (q, 7.2 Hz, 4H), 1.18 (t, 7.2 Hz, 6H) ppm; ^13^C NMR (100 MHz, CDCl_3_): *δ*_C_ = 167.1, 166.6, 147.6, 137.0, 133.6, 130.5, 128.9, 127.6, 126.8, 124.8, 118.4, 111.5, 44.4, 12.5 ppm; HRMS (ESI): calculated for [M + H]^+^ C_18_H_18_BrN_2_O_2_^+^: *m*/*z* = 373.0546; found *m*/*z* = 373.0541; IR: 695 (w), 717 (s), 788 (w), 810 (m), 880 (w), 1082 (m), 1102 (m), 1189 (m), 1276 (w), 1354 (m), 1382 (m), 1519 (s), 1608 (w), 1702 (s), 2700–2970 (w) cm^–1^.

#### 5-Bromo-2-(4-(diethylamino)phenyl)-4*H*-isoindoline (**7**)

A solution of **6** (1.40 g; 3.75 mmol; 1 eq.) in dry THF (30 mL) under argon was cooled to 0 °C, and a solution of borane in THF (1.0 M; 25 mL; 25 mmol; 7 eq.) was slowly added. The mixture was then refluxed for 12 h, cooled to room temperature, poured onto ice (500 mL) and filtered. The precipitate was further purified by filtration over silica (eluent: DCM 98/2 MeOH) to yield **7** as a white powder (0.75 g; 2.17 mmol; 60%). Analytical data: ^1^H NMR (400 MHz, CDCl_3_): *δ*_H_ = 7.47 (s, 1H), 7.41 (d, 8.0 Hz, 1H), 7.20 (d, 8.0 Hz, 1H), 6.88 (d, 9.2 Hz, 2H), 6.63 (d, 9.2 Hz, 2H), 4.57 (s, 2H), 4.55 (s, 2H), 3.23 (q, 7.2 Hz, 4H), 1.09 (t, 7.2 Hz, 6H) ppm; ^13^C NMR (100 MHz, CDCl_3_): *δ*_C_ = 140.8, 140.3, 137.5, 130.1, 125.8, 124.1, 120.7, 118.0, 112.6, 111.8, 53.9, 53.8, 46.0, 12.5 ppm; mass spectroscopy (MALDI-TOF): calculated for [M]^+^ C_18_H_21_BrN_2_^+^*m*/*z* = 344.1; found: *m*/*z* = 344.1; IR: 659 (w), 801 (s), 876 (w), 975 (w), 1015–1100 (w), 1197 (m), 1260 (m), 1335 (m), 1366 (m), 1466 (m), 1518 (s), 2700–2970 (m) cm^–1^.

#### 5-(4,4,5,5-Tetramethyl-1,3,2-dioxaborolan)-2-(4-(diethylamino)phenyl)-4*H*-isoindoline (**8**)

Diphenylphosphinoferrocene palladium(ii) dichloride 1 : 1 complex with DCM (47 mg; 57 μmol; 0.15 eq.), bis(pinacolato)diboron (146 mg; 573 μmol; 1.5 eq.), potassium acetate (75 mg; 764 μmol; 2 eq.) and **7** (150 mg; 382 μmol; 1 eq.) were charged in a 2-neck flask under argon. A degassed solution of dry DMF (20 mL) was then added and the solution was stirred at 90 °C for 6 h. The mixture was poured into water (30 mL), extracted with DCM (3 × 100 mL), dried over MgSO_4_ and filtered. The solution was purified by filtration over SiO_2_ (eluent: DCM/MeOH/NEt_3_: 98/2/2) to obtain **8** with 10% mol mol^–1^ of bis(pinacolato)diboron as a white mixture of powders (118 mg; 302 μmol; 79%). Further chromatography (SiO_2_; dichloromethane 95/5 MeOH) purification for analytical purposes was achieved but resulted in significant material loss. Analytical data: ^1^H NMR (400 MHz, CDCl_3_): *δ*_H_ = 7.78 (s, 1H), 7.74 (d, 8.0 Hz, 1H), 7.34 (d, 8.0 Hz, 1H), 6.88 (br d, 9.2 Hz, 2H), 6.65 (d, 9.2 Hz, 2H), 4.61 (br, 4H), 3.22 (q, 6.8 Hz, 4H), 1.36 (s, 12H), 1.09 (t, 6.8 Hz, 6H) ppm (bispinacolatodiboron impurity: *δ*_H_ = 1.26 (s, 24H) ppm); ^13^C NMR (100 MHz, CDCl_3_): *δ*_C_ = 141.8, 140.8, 139.9, 137.9, 133.6, 128.8, 128.2, 121.9, 118.5, 112.5, 83.8 (2 carbons), 54.3, 53.9, 46.3 (2 carbons), 24.9 (4 carbons), 12.5 (2 carbons) ppm; mass spectroscopy (MALDI-TOF): calculated for [M]^+^ C_24_H_33_BN_2_O_2_^+^*m*/*z* = 392.3; found: *m*/*z* = 392.3; IR: 665 (m), 722 (m), 798 (m), 826 (w), 857 (w), 966 (w), 1074 (w), 1109 (w), 1142 (s), 1200–1250 (w), 1351 (s), 1523 (m), 1739 (m), 2000–2200 (w), 2700–2970 (w) cm^–1^.

#### 2-(4,4,5,5-Tetramethyl-1,3,2-dioxaborolan-2-yl)-5,12-[1,2]benzenotetracene-6,11(5*H*,12*H*)-dione (**10**)

To a solution of **9** (contaminated with 20% mol mol^–1^ anthracene) (effective weight of 0.81 g; 3.1 mmol; 1 eq.) in nitrobenzene (100 mL), was added 1,4-naphthoquinone (7.50 g; 47 mmol; 15 eq.). The solution was purged with argon and stirred for 3 d at 140 °C. Nitrobenzene was then distilled-off under reduced pressure, and the remaining powder was purified by chromatography (SiO_2_; eluent: DCM 1/1 toluene) to remove the excess naphthoquinone and yield the 7-bromotriptycenenaphthoquinone contaminated with 20% of triptycene naphthoquinone (effective weight of the 7-bromotrypticenenaphthoquinone: 1.06 g; 2.6 mmol; 81% yield) as a light-yellow powder [NMR ^1^H CDCl_3_: bromo-triptycenequinone: *δ* = 8.05 (m, 2H), 7.66 (m, 2H), 7.61 (d, 1.8 Hz, 1H), 7.49 (m, 2H), 7.32 (d, 7.8 Hz, 1H), 7.17 (dd, 1H, 7.8 Hz, 1.8 Hz), 7.06 (m, 2H), 5.98 (s, 1H), 5.97 (s, 1H) ppm. (Triptycenenaphthoquinone impurity: *δ* = 8.05 (m, 1H), 7.66 (m, 1H), 7.49 (m, 2H), 7.06 (m, 2H), 5.99 (s, 1H) ppm.)]. The resulting bromo-triptycenenaphthoquinone (118 mg; 287 μmol; 1 eq.), diphenylphosphinoferrocene palladium(ii) dichloride 1 : 1 complex with DCM (30 mg; 37 μmol; 0.12 eq.), bis(pinacolato)diboron (142 mg; 560 μmol; 1.9 eq.) and potassium acetate (85 mg; 861 μmol; 3 eq.) were then charged in a 2-neck flask under argon and dry degassed DMF (40 mL) was added. The solution was stirred at 90 °C for 6 h, poured into water (30 mL), extracted with DCM (3 × 100 mL), dried over MgSO_4_ and filtered. The obtained solution was further purified by chromatography (SiO_2_; eluent: DCM) to remove the triptycenequinone impurity and obtain 90 mg of **9** as a yellow powder (196 μmol; 68%; overall yield over 2 steps 55%). Analytical data: ^1^H NMR (400 MHz, CDCl_3_): *δ*_H_ = 8.04 (m, 2H), 7.92 (s, 1H), 7.65 (m, 2H), 7.49 (dd, 1.2 Hz, 7.2 Hz, 1H), 7.47 (d, 7.2 Hz, 1H), 7.44 (m, 2H), 7.03 (m, 2H), 6.03 (s, 1H), 6.02 (s, 1H), 1.30 (s, 12H) ppm; ^13^C NMR (100 MHz, CDCl_3_): *δ*_C_ = 181.0, 180.9, 153.8, 153.3, 146.8, 143.5, 143.1, 143.0, 133.45, 133.41, 132.5, 131.68, 131.64, 130.1, 126.1, 125.5, 125.4, 124.4, 124.3, 123.9, 83.7, 47.7, 47.5, 24.7, 24.6 ppm; HRMS (ESI): calculated for [M + Na]^+^ C_30_H_25_BNaO_4_^+^: *m*/*z* = 483.1743; found *m*/*z* = 483.1738. Calculated for [M + H]^+^ C_30_H_26_BO_4_: *m*/*z* = 483.1743; found *m*/*z* = 483.1738; IR: 685 (w), 717 (m), 802 (w), 1000–1142 (w), 1216 (s), 1260 (w), 1354 (s), 1458 (m), 1656 (m), 1739 (s), 2853–2970 (m) cm^–1^.

#### [5,15-Bis-(3,5-bis-*tert*-butylphenyl)-10-[5,12-[1,2]benzenotetracen-2-yl-6,11(5*H*,12*H*)-dione]-20-bromoporphinato]zinc(ii) (**12**)

[5,15-Bis-(3,5-bis-*tert*-butylphenyl)-10,20-dibromoporphinato]zinc(ii) (550 mg; 608 μmol; 4 eq.), **10** (70 mg; 152 μmol; 1 eq.), tetrakis(triphenylphosphine)palladium(0) (35 mg; 30 μmol; 0.2 eq.), cesium carbonate (148 mg; 326 μmol; 3 eq.) were refluxed in a toluene/pyridine mixture (20 mL/300 μL) under inert atmosphere. The reaction was followed by crude NMR analysis and stopped after 40 h when no starting boronic ester was detected. The solvents were then removed under vacuum, and **12** was purified by chromatography (SiO_2_; eluent: petrol ether/ethyl acetate/pyridine 10/1/1). This yielded **12** as a purple powder (121 mg; 104 μmol; 69%). Analytical data: ^1^H NMR (400 MHz, CDCl_3_): *δ*_H_ = 9.68 (d, 4.4 Hz, 2H), 8.93 (d, 4.4 Hz, 2H), 8.76 (d, 4.4 Hz, 2H), 8.68 (d, 4.4 Hz, 1H), 8.65 (d, 4.4 Hz, 1H), 8.24 (s, 1H), 8.16 (d, 7.6 Hz, 1H), 8.10 (d, 7.6 Hz, 1H), 7.99 (s, 2H), 7.97 (s, 2H), 7.85 (d, 7.6 Hz, 1H), 7.77–7.69 (m, 4H), 7.66 (m, 7.2 Hz, 2H), 7.55 (d, 7.2 Hz, 1H), 7.16 (m, 2H), 6.30 (s, 1H), 6.15 (s, 1H), 1.51 (s, 9H), 1.504 (s, 9H), 1.497 (s, 9H), 1.49 (s, 9H) ppm.; ^13^C NMR (100 MHz, CDCl_3_): *δ*_C_ = 181.5, 181.4, 154.5, 154.4, 150.7, 150.5, 150.2, 149.4, 148.26, 148.24, 144.0 (2 carbons), 142.0, 141.7, 140.9, 133.7, 133.1, 132.2, 132.11, 132.09, 131.7 (2 carbons), 131.5, 130.4, 129.9, 129.8, 126.43, 126.42, 125.71, 125.68, 124.6 (2 carbons), 122.6, 122.2, 120.6, 120.3, 103.7, 47.9, 47.8, 34.9, 31.7 ppm; mass spectroscopy (MALDI-TOF positive ionization): calculated for [M]^+^ C_72_H_63_BrO_2_N_4_Zn^+^: *m*/*z* = 1160.3; found: *m*/*z* = 1160.3; IR: 711 (m), 794 (m), 901–928 (w), 997 (m), 1216 (s), 1291 (w), 1364 (s), 1450–1500 (w), 1592 (w), 1661 (m), 1739 (s), 2750–2970 (w) cm^–1^.

#### [5,15-Bis-(3,5-bis-*tert*-butylphenyl)-10-[5,12-[1,2]benzenotetracen-2-yl-6,11(5*H*,12*H*)-dione]-20-(4-(diethylamino)phenyl)-4*H*-(isoindolin-2-yl)porphinato]zinc(ii) (**TNQ–ZnPAr–TAPD**)

Tetrakis(triphenylphosphine)palladium(0) (15 mg; 13 μmol; 0.2 eq.), cesium carbonate (62 mg; 190 μmol; 3 eq.), **12** (73 mg; 63 μmol; 1 eq.) and **8** (42 mg; 94 μmol; 1.5 eq.), were refluxed in a toluene/pyridine (10 mL/150 μL) mixture under inert atmosphere. The reaction was followed by TLC and stopped after 3 h. The mixture was evaporated under reduced pressure, purified by chromatography (SiO_2_; eluent: DCM/MeOH/NEt_3_ 98/2/0.5), to remove the fast running side-products and then by size-exclusion column (eluent: chloroform) to remove the excess of **8**. This yielded **TNQ–ZnP_Ar_–TAPD** as a purple powder (61 mg; 45 μmol; 72%). Analytical data: ^1^H NMR (400 MHz, CDCl_3_): *δ*_H_ = 8.97–8.91 (m, 4H), 8.87 (d, 4.6 Hz, 1H), 8.86 (d, 4.6 Hz, 1H), 8.79 (d, 4.6 Hz, 1H), 8.76 (d, 4.6 Hz, 1H), 8.34 (d, 2 Hz, 1H), 8.21–8.12 (m, 4H), 8.07 (d, 1.6 Hz, 2H), 8.05 (d, 1.6 Hz, 2H), 7.94 (m, 1H), 7.83 (d, 7.2 Hz, 1H), 7.78 (t, 1.6 Hz, 1H), 7.77 (t, 1.6 Hz, 1H), 7.71 (m, 3H), 7.67 (d, 8.0 Hz, 1H), 7.60 (d, 6.8 Hz, 1H), 7.19 (m, 2H), 6.97 (d, 8.4 Hz, 2H), 6.81 (d, 8.4 Hz, 2H), 6.36 (s, 1H), 6.21 (s, 1H), 4.97 (s, 2H), 4.90 (s, 2H), 3.27 (q, 6.8 Hz, 4H), 1.55 (s, 9H), 1.54 (s, 9H), 1.53 (s, 9H), 1.52 (s, 9H), 1.16 (t, 6.8 Hz, 6H) ppm; ^13^C NMR (125 MHz, CDCl_3_): *δ*_C_ = 181.5, 181.4, 154.6, 154.5, 154.47, 154.43, 150.25, 150.21, 148.13, 148.11, 144.06, 144.05, 144.05, 142.65, 142.64, 141.6, 141.2, 137.2 (br), 136.5 (br), 133.7, 133.4, 132.12, 132.11, 131.9, 131.7, 131.6, 131.5, 131.3, 131.1, 130.6, 129.9, 129.8, 129.5, 128.5, 128.4, 126.5, 125.7, 125.67, 124.68, 124.65, 123.8, 122.2, 121.99, 121.97, 120.4, 120.1, 119.4, 118.3 (br), 112.7, 112.0, 54.37 (br), 47.9, 47.8, 46.2 (2 carbons), 44.6, 34.9, 31.72, 31.69, 12.6 (2 carbons) ppm; mass spectroscopy (MALDI-TOF positive ionization): calculated for [M]^+^ C_90_H_84_N_6_O_2_Zn^+^: *m*/*z* = 1345.4; found: *m*/*z* = 1345.6; IR: 694 (m), 713 (s), 795 (s), 1016–1150 (s), 1207 (m), 1260 (s), 1363 (m), 1456 (w), 1519 (m), 1745 (w), 1739 (s), 2700–2970 (m) cm^–1^.

#### [5,15-Bis-(3,5-bis-*tert*-butyl-phenyl)-10-(4,4,5,5-tetramethyl-1,3,2-dioxaborolan-2-yl)-20-(4-benzaldehyde)-porphyrinato]zinc(ii) (**14**)

4-Iodobenzaldehyde (325 mg; 1.0 mmol; 4 eq.), **13** (250 mg; 0.25 mmol; 1.0 eq.), freshly prepared Pd(PPh_3_)_4_ (57 mg; 0.05 mmol; 0.2 eq.) and potassium carbonate (100 mg; 0.75 mmol; 3.0 eq.) were refluxed under argon for 80 min at 66 °C in THF (10 mL) and water (3 mL). The reaction was monitored by TLC (DCM/petrol ether 40–60 °C/pyridine 14/85/1). After 5 h, the product was extracted with DCM (3 × 50 mL), dried over MgSO_4_, evaporated and purified by chromatography (SiO_2_; eluent: DCM/petrol ether/pyridine 14/85/1) to yield **14** as a purple powder (85 mg; 0.087 mmol; 35%). Analytical data: ^1^H NMR (200 MHz, CDCl_3_ + 1% pyridine-*d*_5_): *δ*_H_ = 10.33 (s, 1H), 9.96 (d, 4.7 Hz, 2H), 9.16 (d, 4.7 Hz, 2H), 9.04 (d, 4.6 Hz, 2H), 8.90 (d, 4.6 Hz, 2H), 8.43 (d, 8.0 Hz, 2H), 8.22 (d, 8.1 Hz, 2H), 8.15 (d, 3.6 Hz, 4H), 7.89 (t, 3.6 Hz, 2H), 1.92 (s, 12H), 1.64 (s, 36H) ppm; ^13^C NMR (100 MHz, CDCl_3_ + 1% pyridine-*d*_5_): *δ*_C_ = 192.6, 154.2, 150.5, 150.4, 148.2, 148.1, 142.4, 135.0, 133.1, 132.3, 131.8, 130.9, 129.9, 127.5, 122.1, 120.5, 119.8, 85.0, 35.0, 31.8, 25.3 ppm; mass spectroscopy (MALDI-TOF positive ionization): calculated for [M + H]^+^ C_61_H_68_BN_4_O_3_Zn^+^: *m*/*z* = 979.46; found *m*/*z* = 980.60.

#### [5,15-Bis-(3,5-bis-*tert*-butylphenyl)-10-(4-caboxyphenyl)-20-(4-(diethylamino)phenyl)-4*H*-(isoindolin-2-yl)porphinato]zinc(ii) (**15**)

Tetrakistriphenylphosphine palladium(0) (17.7 mg; 0.015 mmol; 0.1 eq.), potassium carbonate (63 mg; 0.45 mmol; 3.0 eq.), **14** (150 mg; 0.15 mmol; 1.0 eq.) and **7** (104 mg; 0.30 mmol; 2.0 eq.) were refluxed under argon in THF (6.0 mL) and water (2.0 mL) for 5 h. The solvent was removed under vacuum and the mixture was purified by chromatography to remove hydrogenated side-products (SiO_2_; DCM/MeOH/NEt_3_ 99/1/0.5), followed by a size exclusion chromatography (CHCl_3_ + 1% pyridine) to remove the excess of **7** and yield **15** as a purple powder (149 mg; 0.133 mmol; 88%). Analytical data: ^1^H NMR (400 MHz, CDCl_3_ + 1% pyridine-*d*_5_): *δ*_H_ = 10.37 (s, 1H), 8.95–8.89 (m, 6H), 8.80 (d, 4.8 Hz, 2H), 8.39 (d, 7.4 Hz, 2H), 8.22 (d, 8.2 Hz, 2H), 8.14 (s, 1H), 8.12 (d, 7.6 Hz, 1H), 8.05 (s, 4H), 7.77 (s, 2H), 7.74 (d, 7.6 Hz, 1H), 6.93 (d, 8.6 Hz, 2H), 6.79 (d, 8.6 Hz, 2H), 4.94 (s, 2H), 4.87 (s, 2H), 3.25 (q, 6.8 Hz, 4H), 1.52 (m, 36H), 1.13 (t, 6.8 Hz, 6H) ppm; ^13^C NMR (100 MHz, CDCl_3_ + 1% pyridine-*d*_5_): *δ*_C_ = 192.5, 150.5, 150.3, 149.1, 148.3, 142.5, 142.3, 140.9, 140.1, 137.4, 136.6, 135.14, 135.09, 133.3, 132.3, 132.1, 131.6, 130.7, 129.8, 128.5, 127.6, 123.2, 123.0, 122.7, 122.3, 120.7, 120.5, 120.2, 118.2, 112.7, 54.4 (2 carbons), 46.1, 35.0, 31.7, 12.6 ppm; mass spectroscopy (MALDI-TOF positive ionization): calculated for [M + H]^+^ C_73_H_77_N_6_OZn^+^: *m*/*z* = 1117.54; found: *m*/*z* = 1118.00.

#### [5,15-Bis-(3,5-bis-*tert*-butylphenyl)-10-[4-(*N*-(3′,4′-didodecoxybenzyl)-([60]fullero[*c*]tetrahydropyrrol-2-yl)phenyl)]-20-(4-(diethylamino)phenyl)-4*H*-(isoindolin-2-yl)porphinato]zinc(ii) (**C_60_–ZnPAr–TAPD**)

**C_60_** (13.2 mg, 18.0 μmol, 2 eq.) was sonicated in dry toluene (2 mL) for 45 min. **15** (10 mg, 9.0 μmol, 1 eq.) and 2-((3,4-bis(dodecyloxy)benzyl)amino)acetic acid (see ESI†) (48 mg; 90 μmol; 10 eq.) were added. The solution was then purged with argon and subsequently heated to reflux for 135 min. The crude mixture was poured onto silica gel and eluted with a N_2_-purged toluene/DCM/pyridine 70/29/1 mixture to remove the fast running **C_60_** excess. The second fraction (a green solution, due to coordination of pyridine) was then further purified by size-exclusion chromatography (eluent: toluene) to collect the first band (red). Flash chromatography was repeated on silica gel eluting with toluene/DCM/pyridine 70/29/1 mixture to yield **C_60_–ZnP_Ar_–TAPD** (4.9 mg; 2.2 μmol; 22%) as a brown powder. Analytical data: ^1^H NMR (700 MHz, CD_2_Cl_2_ : CS_2_ : pyridine-*d*_*5*_ = 48 : 50 : 2 (v/v/v)): *δ*_H_ = 8.89 (m, 8H), 8.32 (br, 4H), 8.15 (s, 1H), 8.12 (d, 7.6 Hz, 1H), 8.05 (m, 4H), 7.77 (s, 2H), 7.67 (d, 7.6 Hz, 1H), 7.39 (d, 1.9 Hz, 1H), 7.35 (dd, 8.4, 1.9 Hz, 1H), 7.04 (d, 8.4 Hz, 1H), 6.84 (d, 8.8 Hz, 2H), 6.74 (d, 8.8 Hz, 2H), 5.52 (s, 1H), 5.02 (d, 8.3 Hz, 1H), 4.95 (d, 13.5 Hz, 1H), 4.94 (s, 2H), 4.88 (s, 2H), 4.34 (d, 8.3 Hz, 1H), 4.16 (t, 6.6 Hz, 2H), 4.05 (t, 6.6 Hz, 2H), 3.96 (d, 13.4 Hz, 1H), 3.26 (q, 6.9 Hz, 4H), 1.86 (m, 4H), 1.51 (m, 36H), 1.27–1.19 (br, 36H), 1.13 (t, 6.9 Hz, 6H), 0.90–0.83 (m, 6H) ppm; mass spectroscopy (MALDI-TOF positive ionization): calculated for [M + H]^+^ C_166_H_134_N_7_O_2_Zn^+^: *m*/*z* = 2310.99; found: *m*/*z* = 2311.02.

## Supplementary Material

Supplementary information
