# Supplementary material for: Synthesis and investigation of donor–porphyrin–acceptor triads with long-lived photo-induced charge-separate states
Source: Chem Sci. 2015 Jul 31;6(11):6468–81. doi: 10.1039/c5sc01830g (PMC6054115; doi:10.1039/c5sc01830g)
Supplement: Supplementary file 1 [file SC-006-C5SC01830G-s001.pdf]

Electronic Supplementary Material (ESI) for Chemical Science.
This journal is © The Royal Society of Chemistry 2015

Electronic Supplementary Information

Synthesis and investigation of donor-porphyrin-acceptor triads with long-lived photo-induced charge-separate states

Julien B. Kelber, Naitik A. Panjwani, Di Wu, Rafael Gómez-Bombarelli, Brendon W. Lovett, John J. L. Morton and Harry L. Anderson

TABLE OF CONTENTS

page

1) ***General information*** **S2**

2) ***Characterization of reaction intermediates and new compounds*** **S3**
   - (a) 2-(4-Nitrophenyl)isoindoline-1,3-dione-5-benzoic acid (**1**) S3
   - (b) (2-(4-Nitrophenyl)isoindolin-5-yl)methanol (**2**) S5
   - (c) (2-(4-Nitrophenyl)isoindoline-5-carbaldheyde (**3**) S7
   - (d) 5-Bromo-2-(4-(diethylamino)phenyl)isoindoline-1,3-dione (**6**) S9
   - (e) 5-Bromo-2-(4-(diethylamino)phenyl)-4H-isoindoline (**7**) S11
   - (f) 5-(4,4,5,5-Tetramethyl-1,3,2-dioxaborolan)-2-(4-(diethylamino)phenyl)-4H-isoindoline (**8**) S13
   - (g) 2-Bromoanthraquinone S15
   - (h) 2-Bromoanthracene (mixture with anthracene) (**9**) S15
   - (i) 2-Bromo-5,12-[1,2]benzenotetracene-6,11(5H,12H)-dione S16
   - (j) 2-(4,4,5,5-Tetramethyl-1,3,2-dioxaborolan-2-yl)-5,12-[1,2]benzenotetracene-6,11(5H,12H)-dione (**10**) S17
   - (k) Bromoporphyrin **12** S19
   - (l) **TNQ-ZnP$_{Ar}$-TAPD** S22
   - (m) Porphyrin aldehyde **14** S30
   - (n) Porphyrin aldehyde **15** S31
   - (o) **C_{60}-ZnP$_{Ar}$-TAPD** S33

3) ***Synthesis of reference and known compounds*** **S37**
   - (a) Porphyrin boronic acids **13** and **S1** S37
   - (b) 2-((3,4-Bis(dodecyloxy)benzyl)amino)acetic acid (**S3**) S39
   - (c) Porphyrins **S4** and **S5** S41

4) ***Assignment of the TAPD-ZnP$_{Ar}$-TNQ triad ^{1}H NMR spectrum using reference compounds*** **S43**

5) ***Frontier orbitals energies of triads*** **S44**

6) ***Frontier orbitals distribution of triads*** **S45**

7) ***Ionization potential and electron affinities of moieties*** **S47**

8) ***Inner-sphere reorganization energies*** **S48**

9) ***Predicted recombination rates*** **S49**

10) ***Analysis of the influence of conformational factors on electronic coupling*** **S50**

11) ***Cambridge database analysis of the dihedral angles between meso-phenyls and porphyrin planes*** **S52**

12) ***Methods and kinetic modeling of the Electronic Paramagnetic Resonance (EPR) experiments*** **S53**

13) ***References*** **S56**

1) General information

All chemical reagents were used as received. 2-Bromoanthracene (ref S1) and [5,15-bis-(3,5-bis-*tert*-butylphenyl)-10,20-bisbromoporphinato]zinc(II) (ref S2) were synthesized following a literature procedure. Dichloromethane (DCM) and tetrahydrofuran (THF) were dried over activated alumina prior to use. Anhydrous *N,N*-dimethylformamide (DMF), acetic acid, nitrobenzene, pyridine, toluene, xylenes and anhydrous 2-methyltetrahydrofuran (MTHF) were supplied by Aldrich and used without further purification. Purge gas was high purity argon. Chromatography was performed on silica (200–400 mesh). All reported ^{1}H NMR spectra were collected using a 400 MHz (Bruker AVII 400) or a 500 MHz (Bruker AVII 500) spectrometer. Chemical shifts (in the ppm scale) were determined versus TMS using the residual solvent peak as the internal reference ($C\underline{H}Cl_3$, δ = 7.26 ppm). Deuterated chloroform was stored over potassium carbonate to avoid any acid trace. The UV/Vis absorption spectra were recorded in MTHF using a Perkin Elmer Lambda 20 UV-Vis Spectrometer. The absorption wavelengths are reported in nm with the extinction coefficient in M^{-1} cm^{-1}. Infra-red spectra were recorded in the solid state (neat) using a Bruker Tensor27 FT-IR spectrometer. Mass spectroscopy was performed either on ESI-TOF (Waters LCT Premier) or on MALDI-TOF (Waters MALDI Micro MX) spectrometer. Preparative scale size exclusion chromatography (SEC) was carried out using BioRad Bio-Beads S-X1 with toluene as eluent. ESR samples were prepared in 3.8 mm quartz tubes, sealed under vacuum and kept at 77 K in the dark.

2) Characterization of new compounds

a. 2-(4-Nitrophenyl)isoindoline-1,3-dione-5-benzoic acid (**1**)

^{1}H NMR ; 400MHz ; DMSO-d_6+ H_2O

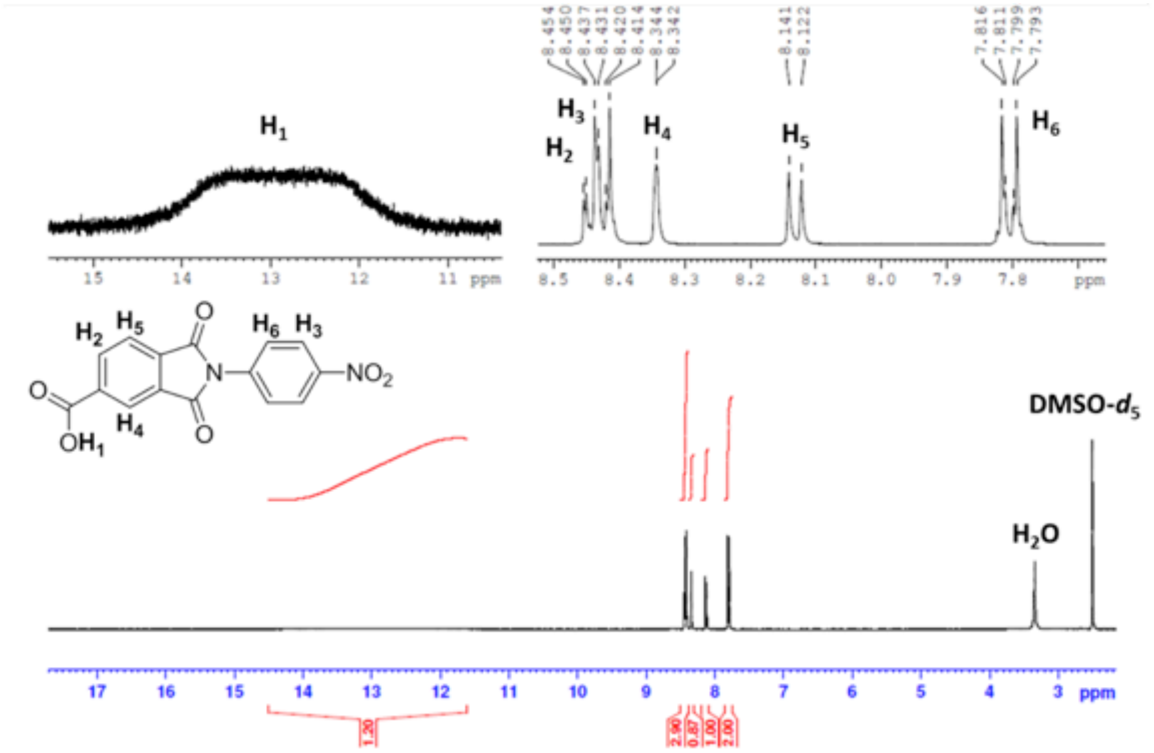

^{13}C NMR ; 100MHz ; DMSO-d_6+ H_2O

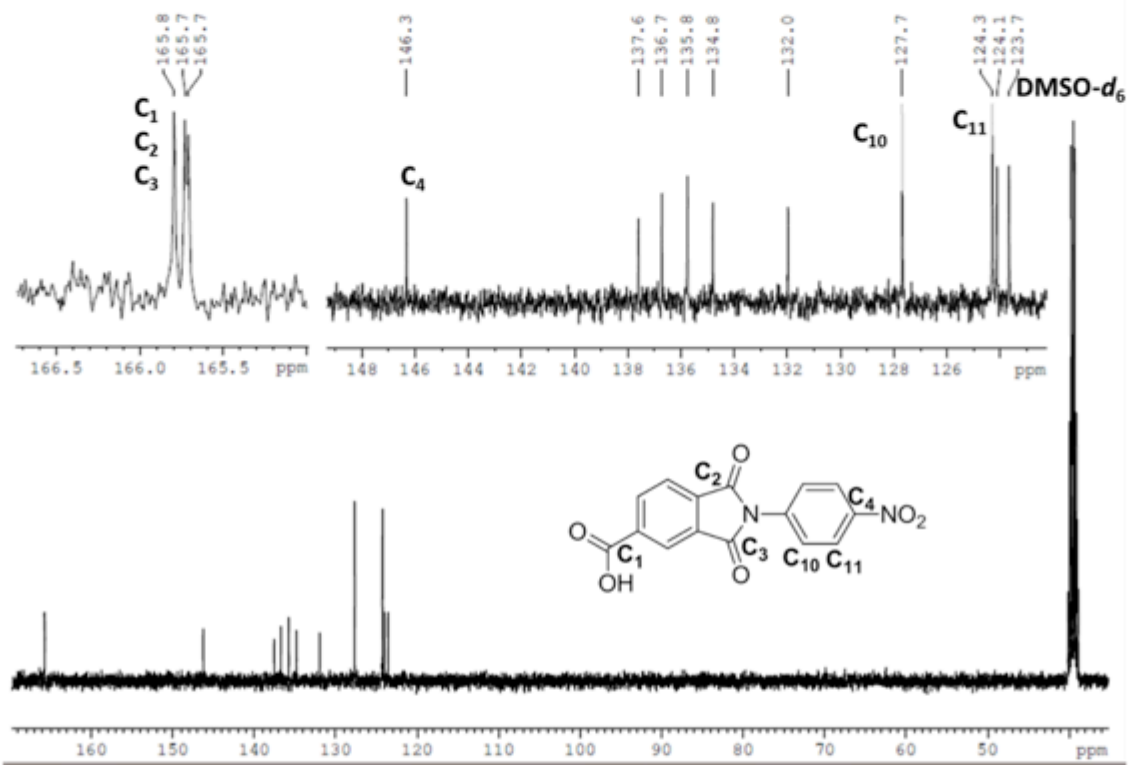

Mass Spectrum SmartFormula Report

Analysis Info

Analysis Name	\\Utof\data\Mar 14\ESI45309_4_01_17597.d	Acquisition Date	17/03/2014 08:09:07
Method	2.5min_cal_sample_pos_naf_05-08-13.m	Operator	Mass Spec
Sample Name	ESI45309	Instrument / Ser#	micrOTOF 92
Comment			

Acquisition Parameter

Source Type	ESI	Ion Polarity	Positive	Set Nebulizer	2.0 Bar
Focus	Not active			Set Dry Heater	180 °C
Scan Begin	100 m/z	Set Capillary	4500 V	Set Dry Gas	10.0 l/min
Scan End	1000 m/z	Set End Plate Offset	-500 V	Set Divert Valve	Source

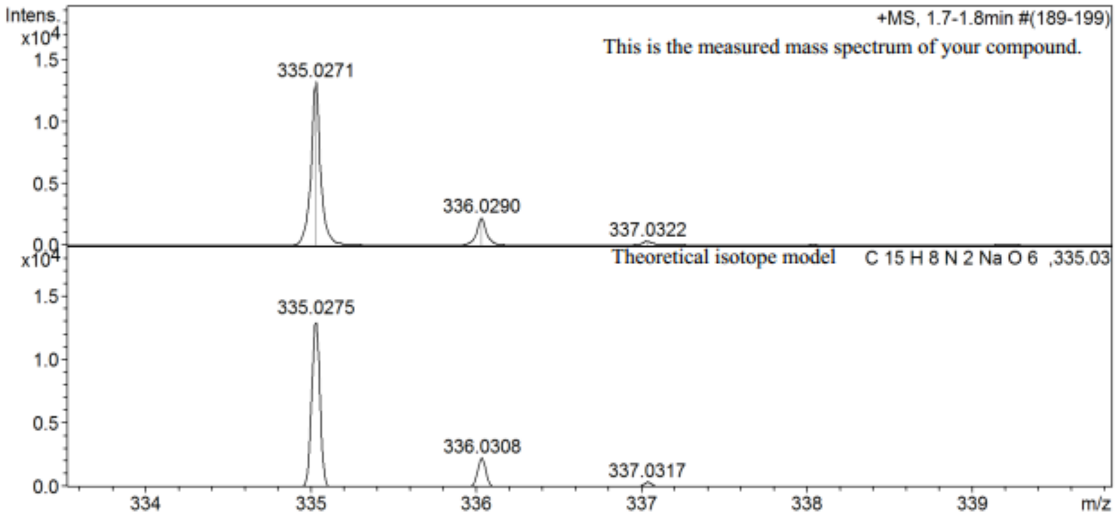

Meas. m/z	#	Formula	m/z	err [ppm]	Mean err [ppm]	rdb	e¯ Conf	mSigma
335.0271								
	1	C 15 H 8 N 2 Na O 6	335.0275	1.1	1.6	12.5	even	1.43

b. (2-(4-Nitrophenyl)isoindolin-5-yl)methanol (**2**)

^{1}H NMR ; 400MHz ; DMSO-d_6+ H_2O

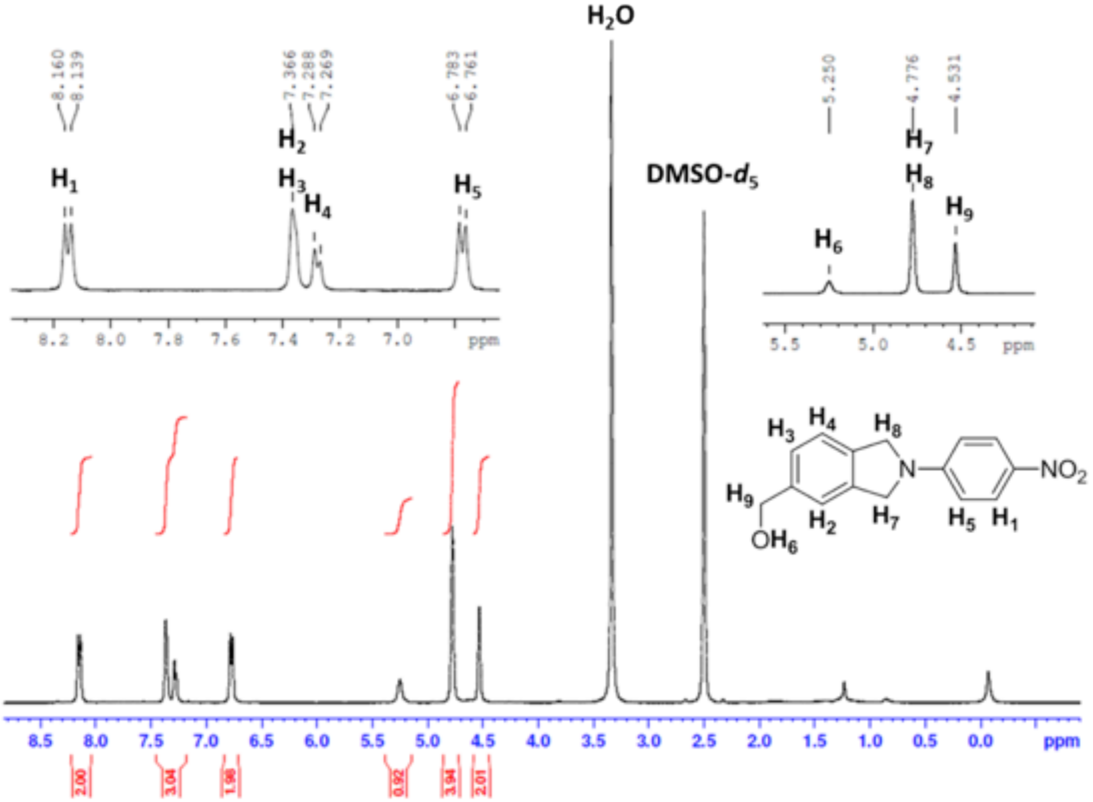

^{13}C NMR ; 100MHz ; DMSO-d_6+ H_2O

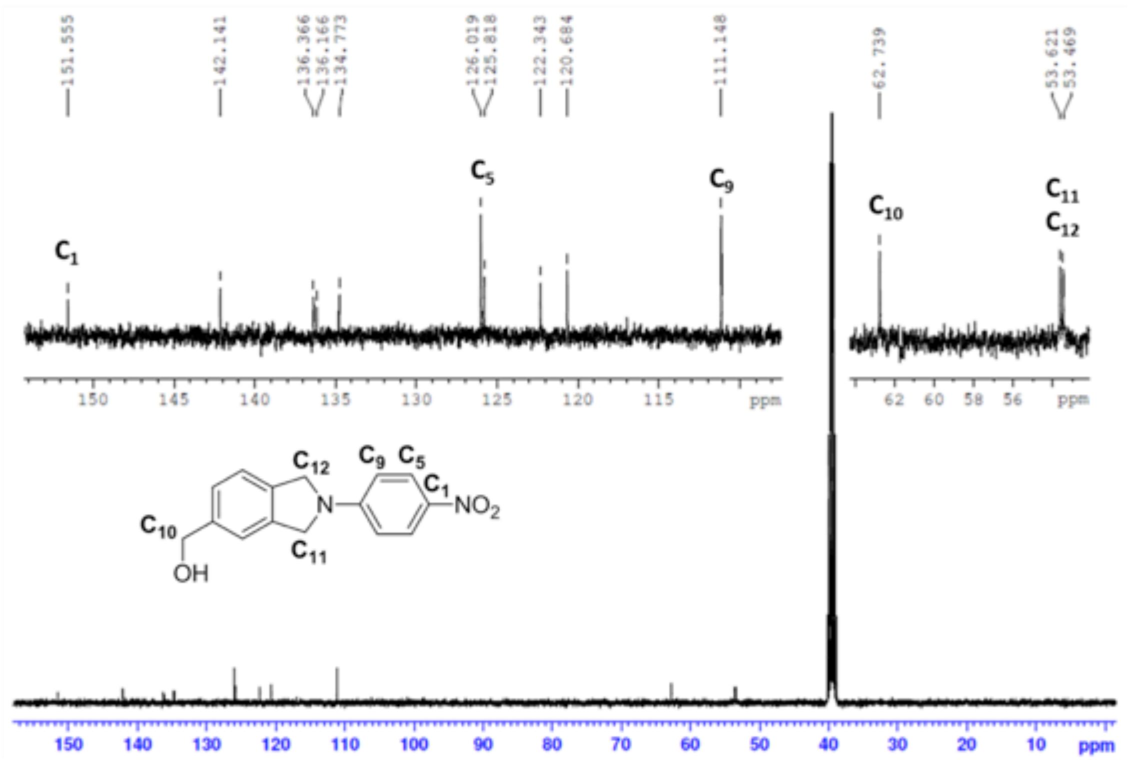

Data File: D:\EZXDATA\CHEMIST\62957-ALCOHOLISOINDOLIN-NO21-101213.D
User Name: Julien kelber
Sample Name: alcoholisoindolin-NO2
Location: P4-C-08
Sample Info: Easy-Access Method: '1_POS1000' ->
Method Info: Positive open access method
105-1000

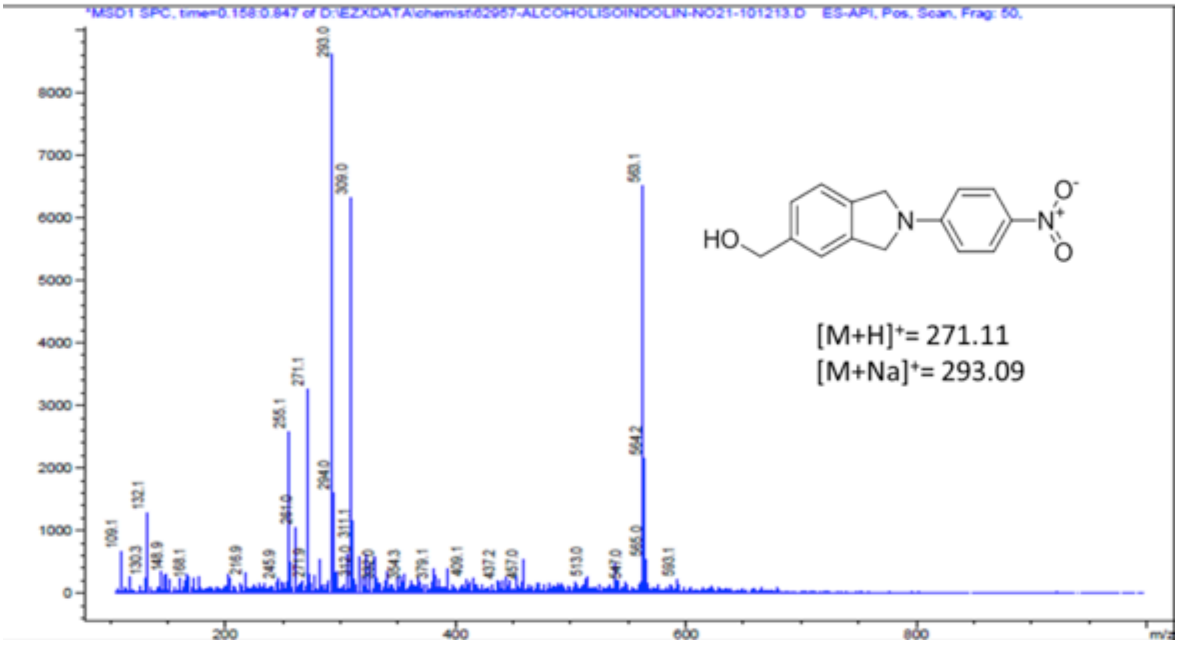

ESI+ HRMS

Analysis Info

Analysis Name	\\Utof\data\Dec 13\ESI43875_12_01_14935.d	Acquisition Date	11/12/2013 09:05:07
Method	2.5min_cal_sample_pos_naf_05-08-13.m	Operator	Mass Spec
Sample Name	ESI43875	Instrument / Ser#	micrOTOF 92
Comment			

Acquisition Parameter

Source Type	ESI	Ion Polarity	Positive	Set Nebulizer	2.0 Bar
Focus	Not active			Set Dry Heater	180 °C
Scan Begin	100 m/z	Set Capillary	4500 V	Set Dry Gas	10.0 l/min
Scan End	1000 m/z	Set End Plate Offset	-500 V	Set Divert Valve	Source

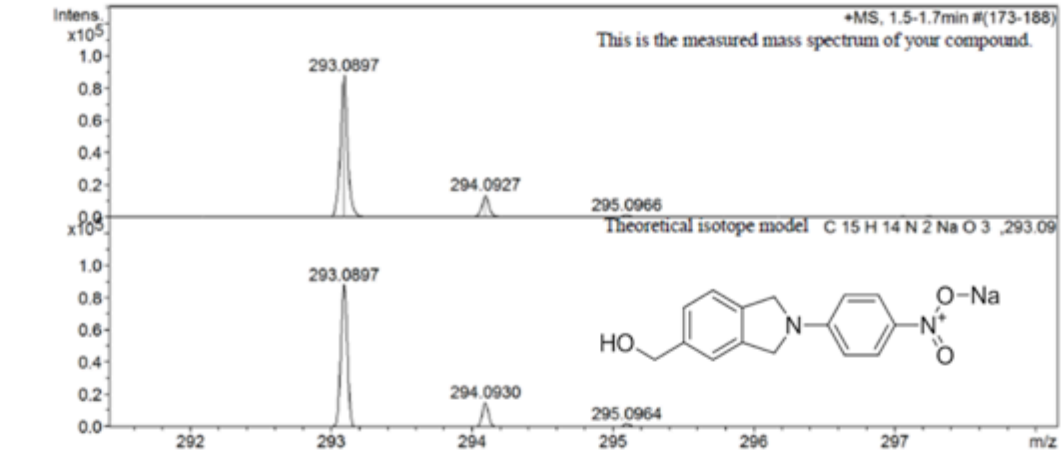

Meas. m/z	#	Formula	m/z	err [ppm]	Mean err [ppm]	rdb	e⁻ Conf	mSigma
293.0897								
	1	C 15 H 14 N 2 Na O 3	293.0897	-0.1	0.1	9.5	even	6.97

c. (2-(4-Nitrophenyl)isoindoline-5-carbaldheyde (**3**)

^{1}H NMR ; 400MHz ; $CDCl_3$+ K_2CO_3

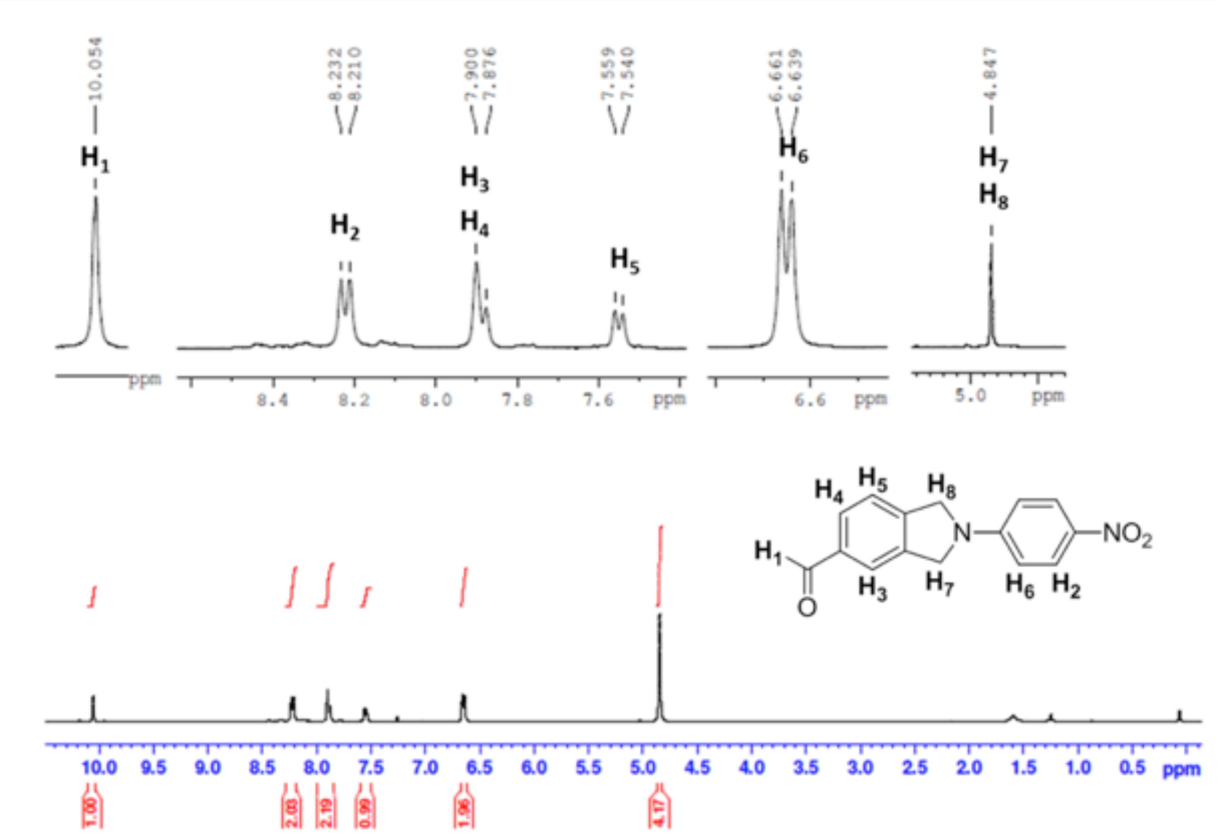

^{13}C NMR ; 100MHz ; $CDCl_3$+ K_2CO_3

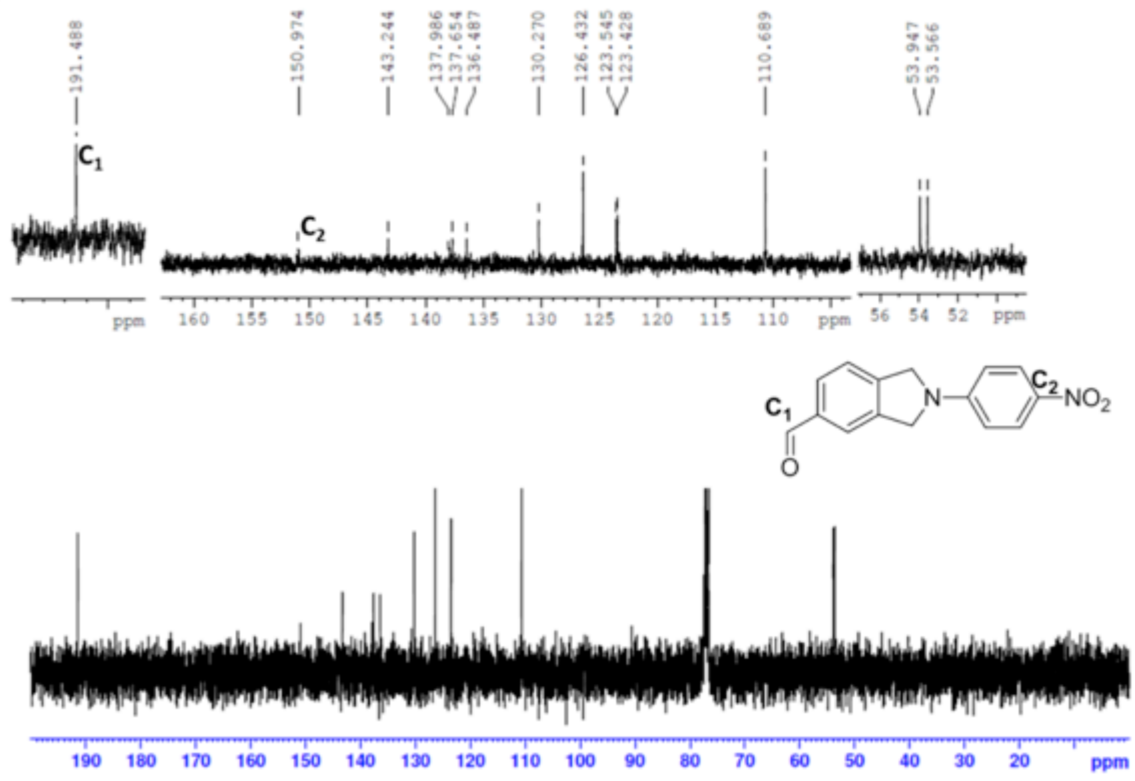

Mass Spectrum SmartFormula Report

Analysis Info

Analysis Name	\\Utof\data\Mar 14\ESI45310_5_01_17598.d	Acquisition Date	17/03/2014 08:12:59
Method	2.5min_cal_sample_pos_naf_05-08-13.m	Operator	Mass Spec
Sample Name	ESI45310	Instrument / Ser#	micrOTOF 92
Comment			

Acquisition Parameter

Source Type	ESI	Ion Polarity	Positive	Set Nebulizer	2.0 Bar
Focus	Not active			Set Dry Heater	180 °C
Scan Begin	100 m/z	Set Capillary	4500 V	Set Dry Gas	10.0 l/min
Scan End	1000 m/z	Set End Plate Offset	-500 V	Set Divert Valve	Source

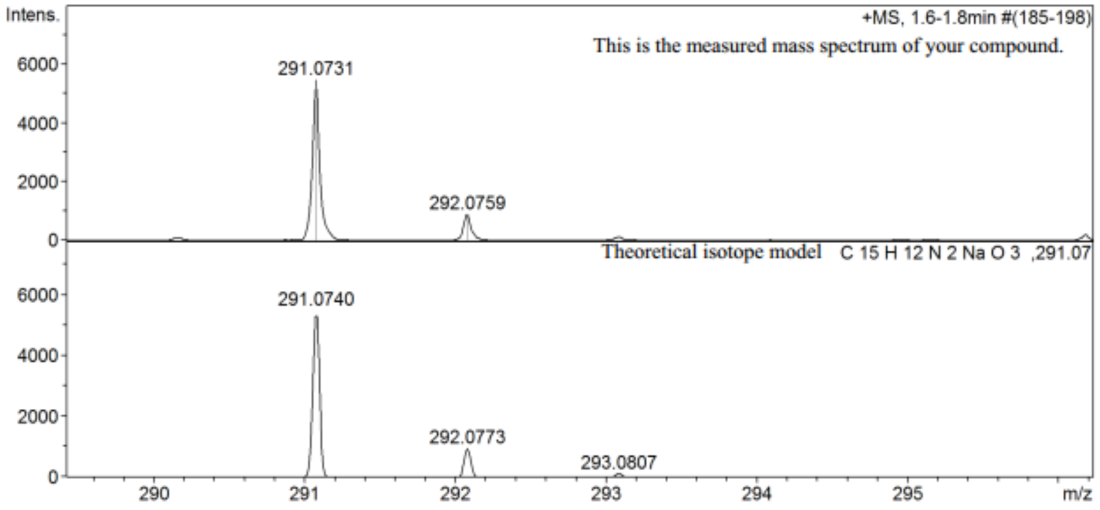

Meas. m/z	#	Formula	m/z	err [ppm]	Mean err [ppm]	rdb	e⁻ Conf	mSigma
291.0731								
	1	C 15 H 12 N 2 Na O 3	291.0740	3.2	3.5	10.5	even	12.72

d. 5-Bromo-2-(4-(diethylamino)phenyl)isoindoline-1,3-dione (6)

^{1}H NMR ; 400MHz ; $CDCl_3$+ K_2CO_3

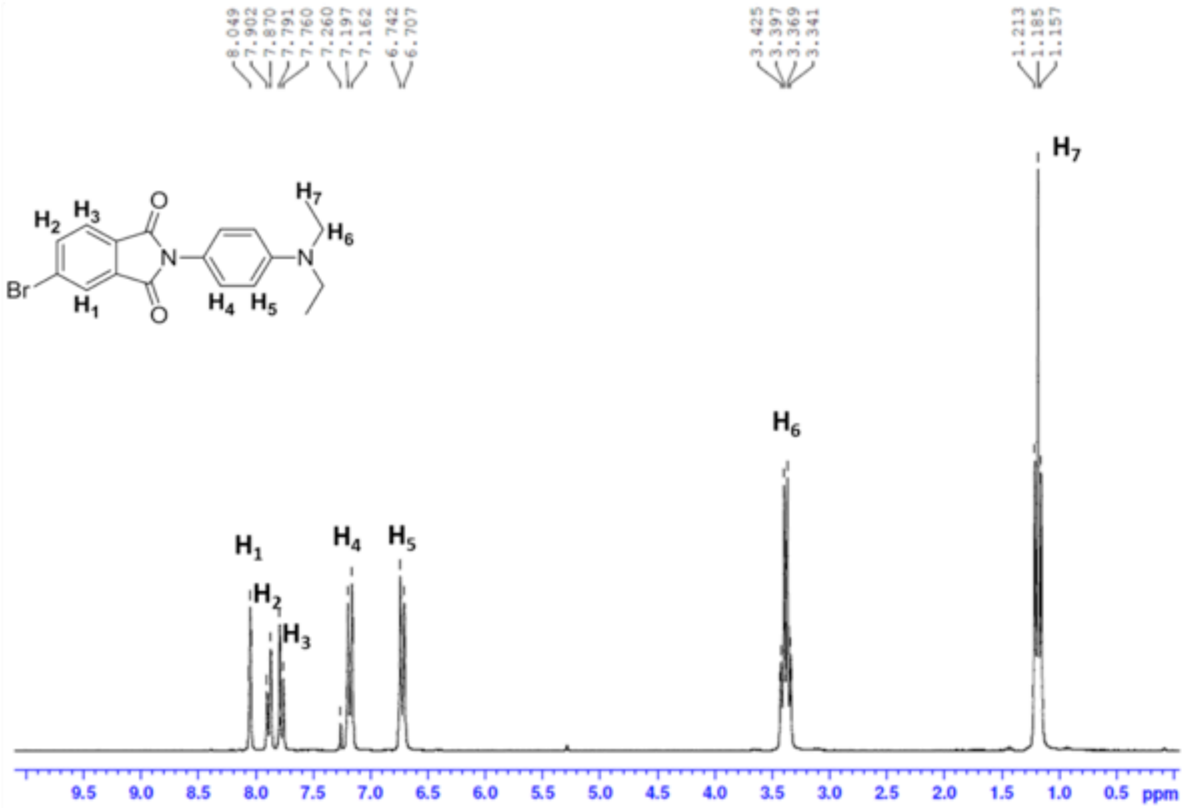

^{13}C NMR ; 100MHz ; $CDCl_3$+ K_2CO_3

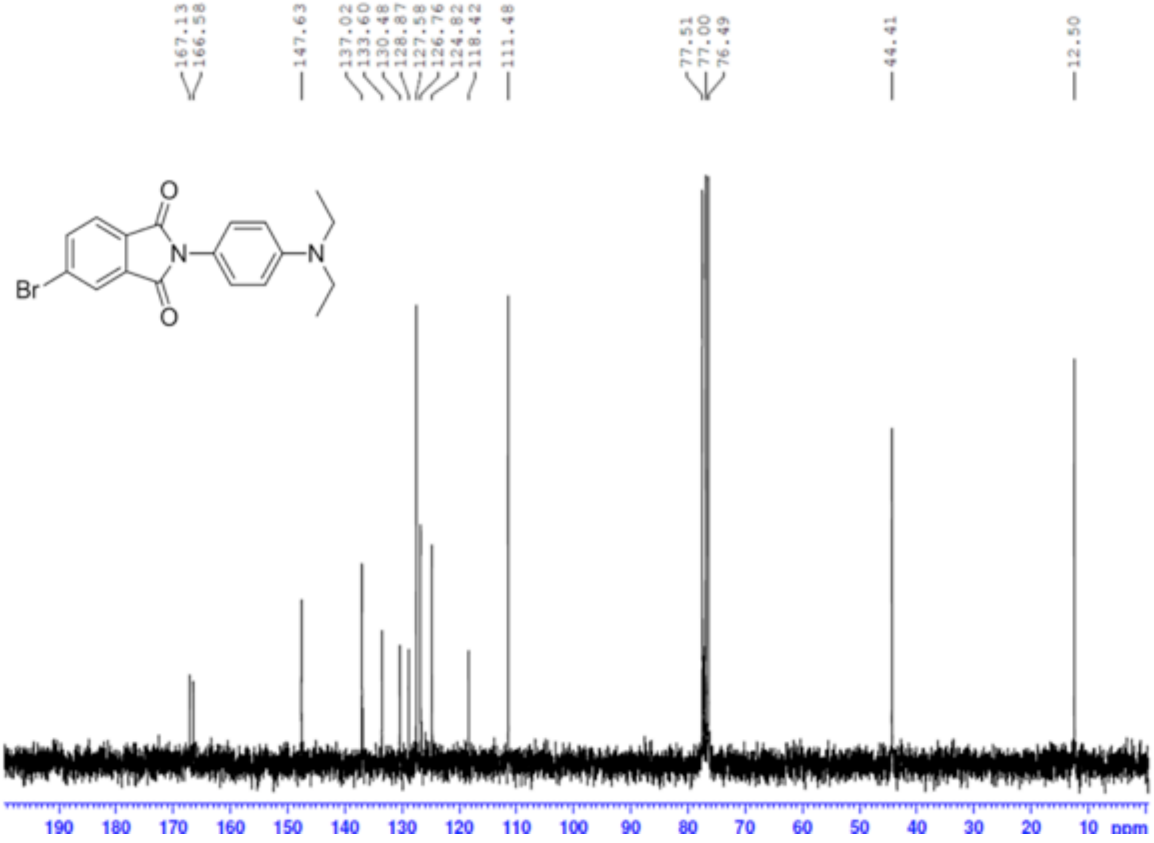

ESI+ HRMS

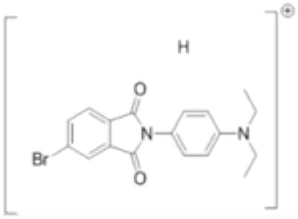

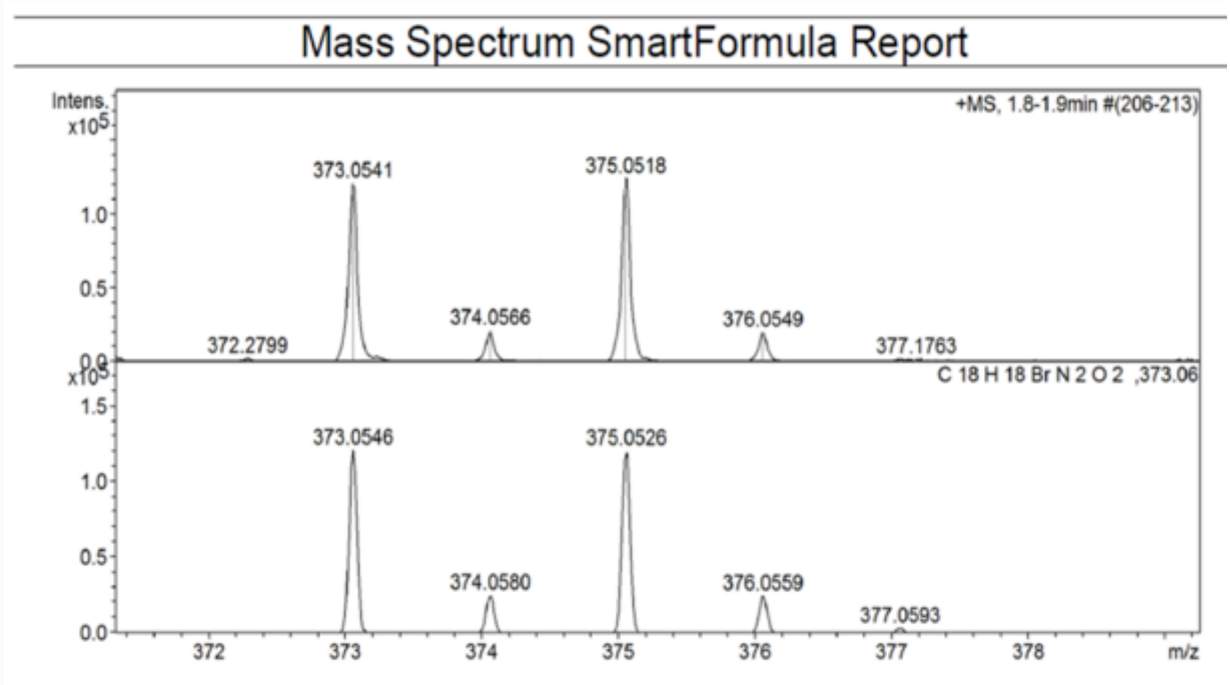

Infra-red vibrational spectroscopy

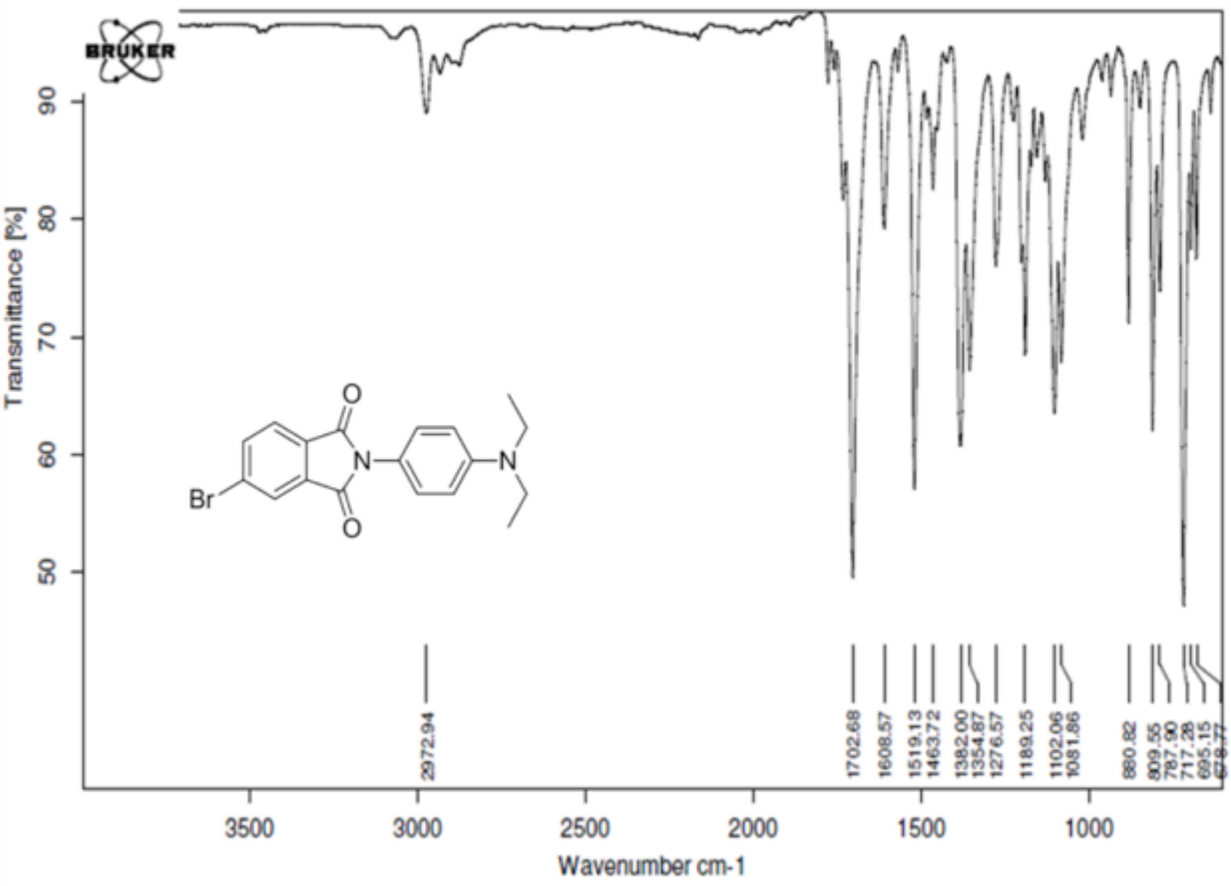

e. 5-Bromo-2-(4-(diethylamino)phenyl)-*4H*-isoindoline (7)

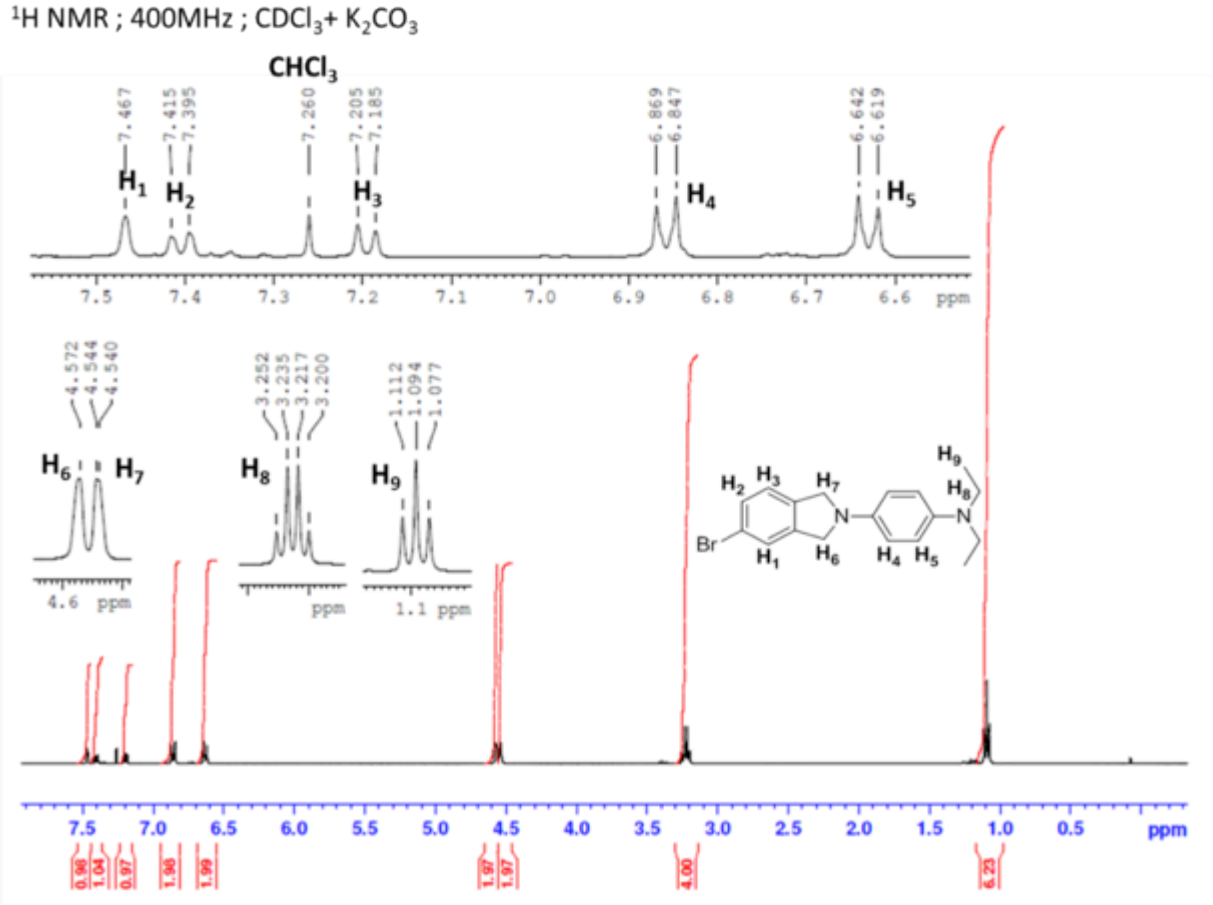

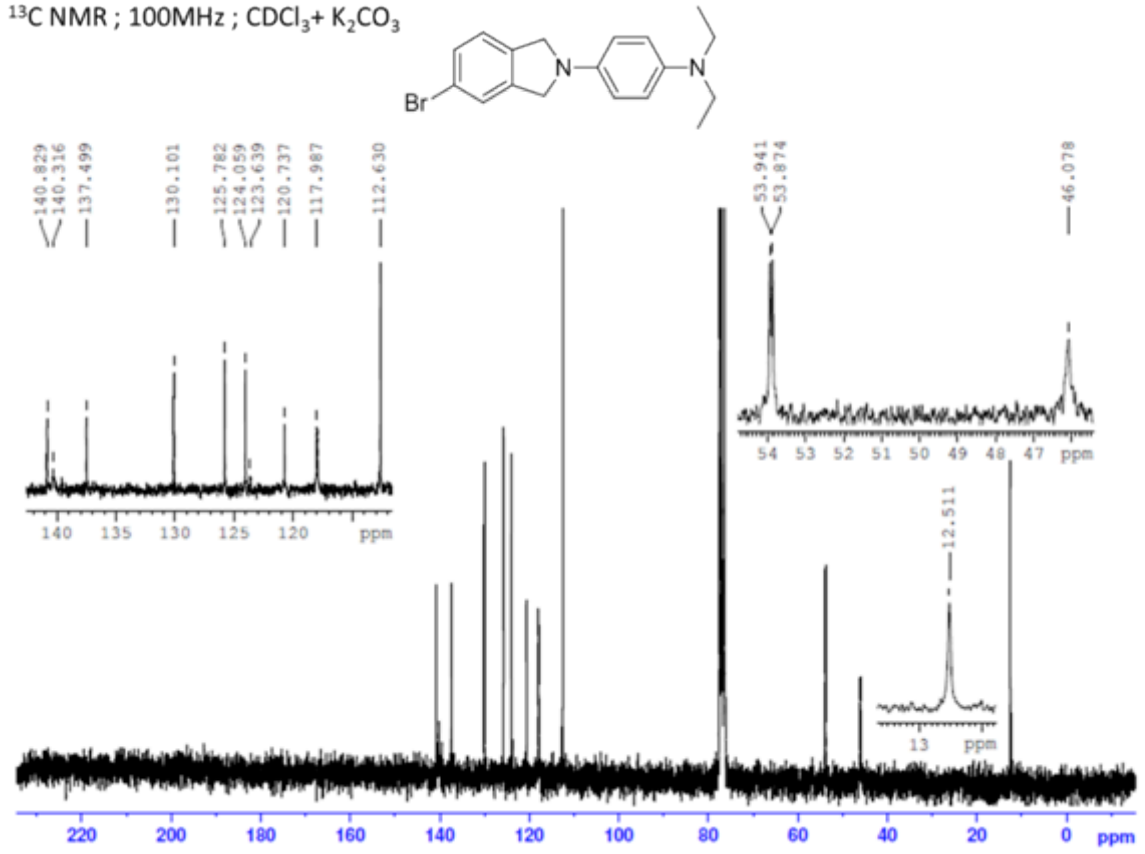

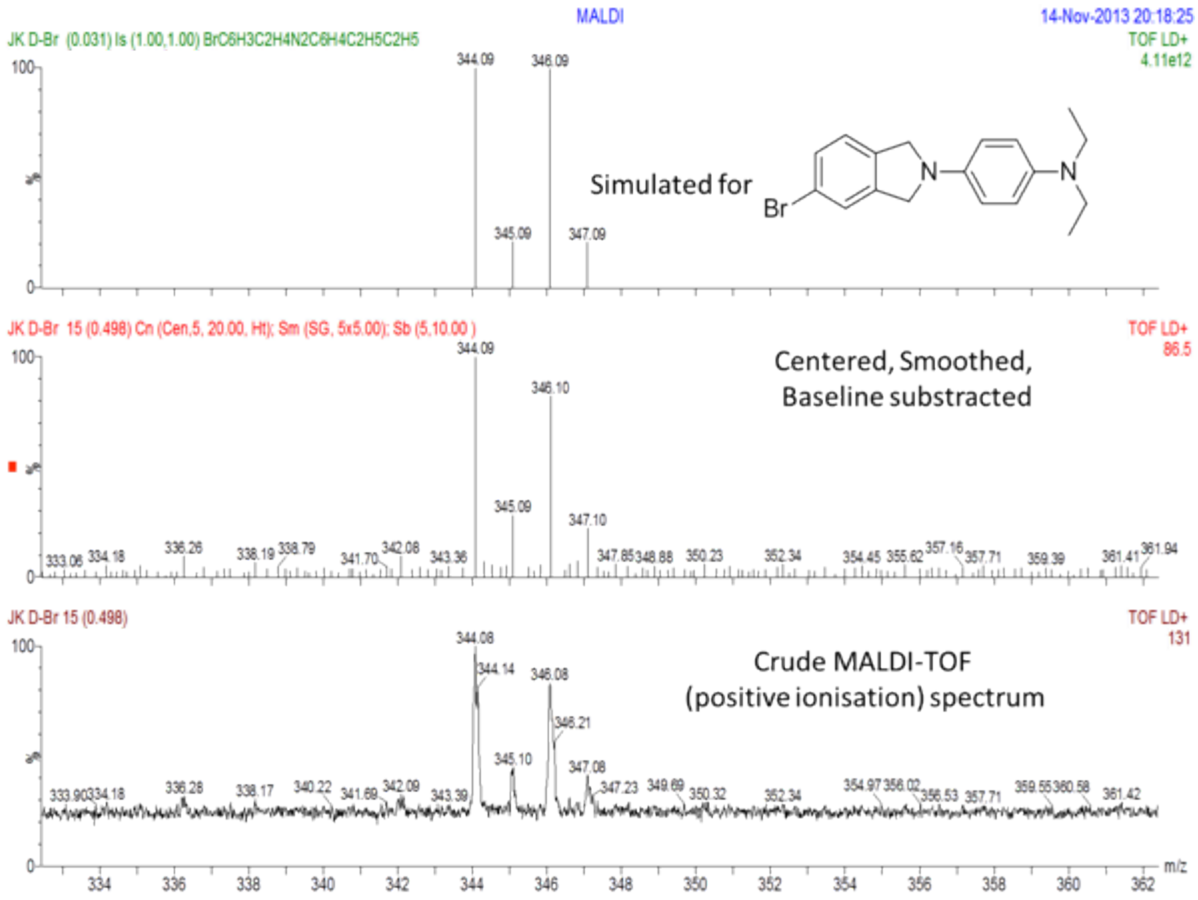

Infra-red vibrational spectroscopy

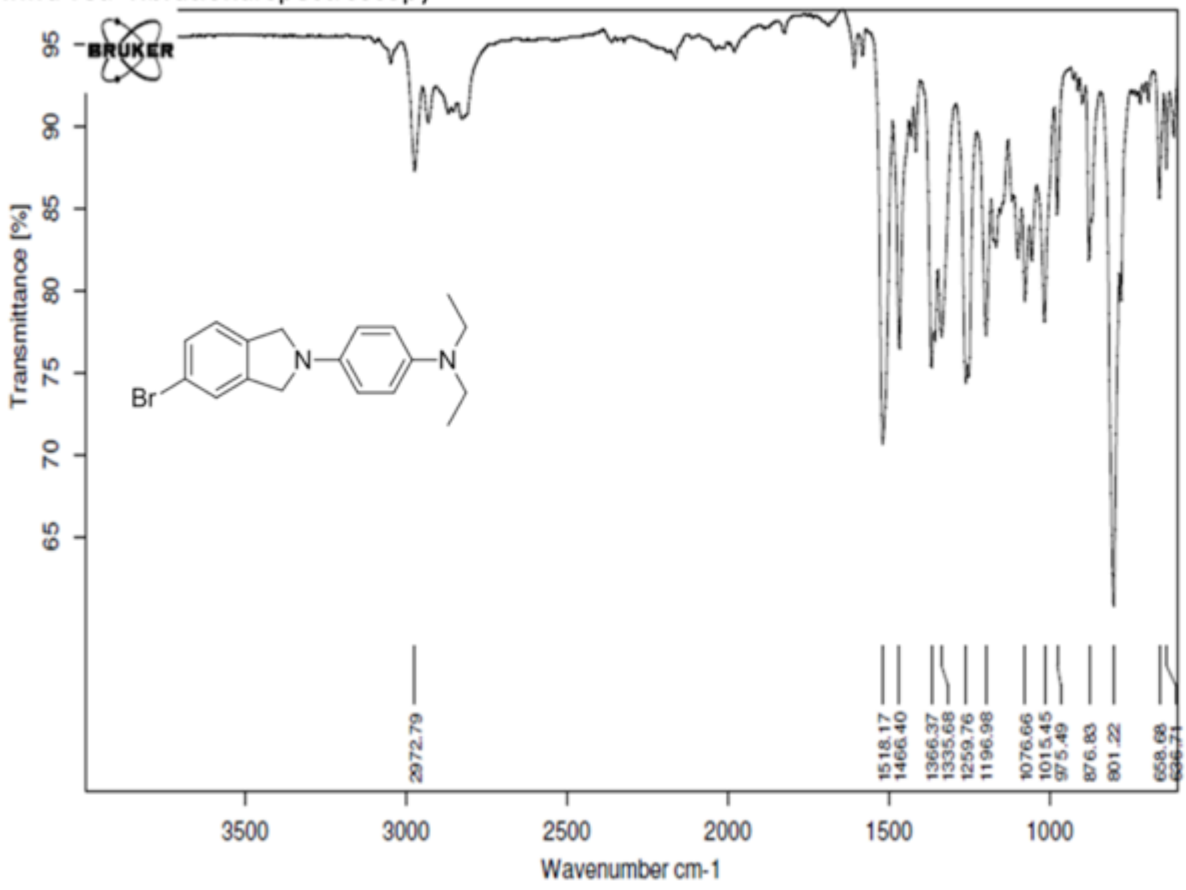

f. **5-(4,4,5,5-Tetramethyl-1,3,2-dioxaborolan)-2-(4-(diethylamino)phenyl)-*4H*-isoindoline (8)**

^{1}H NMR ; 400MHz ; $CDCl_3$ + K_2CO_3

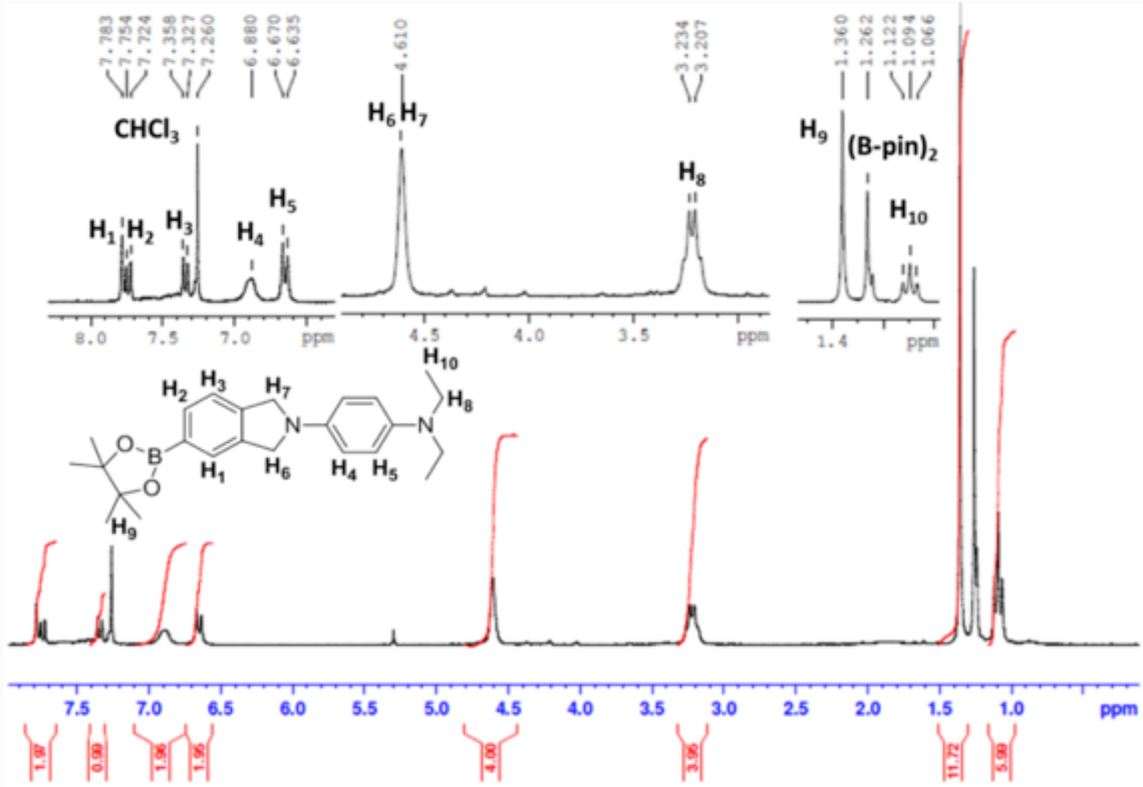

^{13}C NMR ; 100MHz ; $CDCl_3$+ K_2CO_3

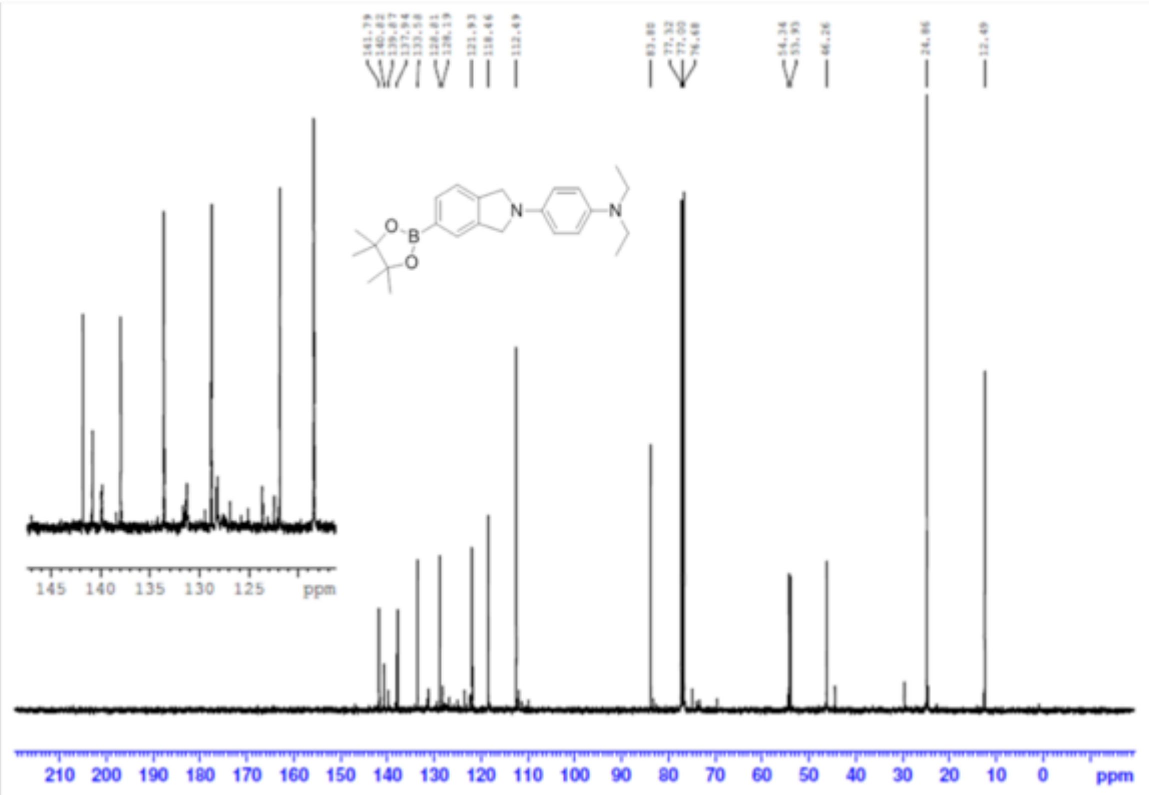

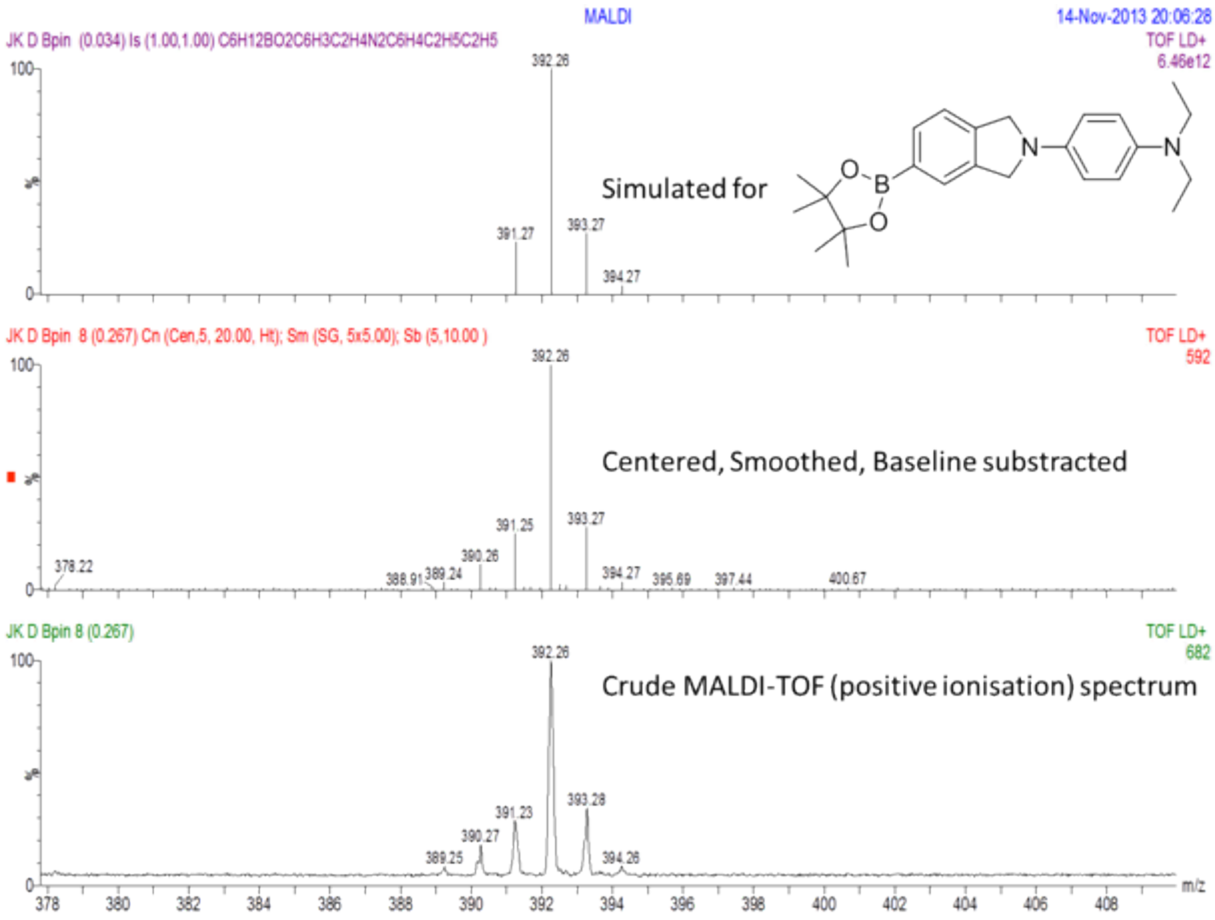

Infra-red vibrational spectroscopy

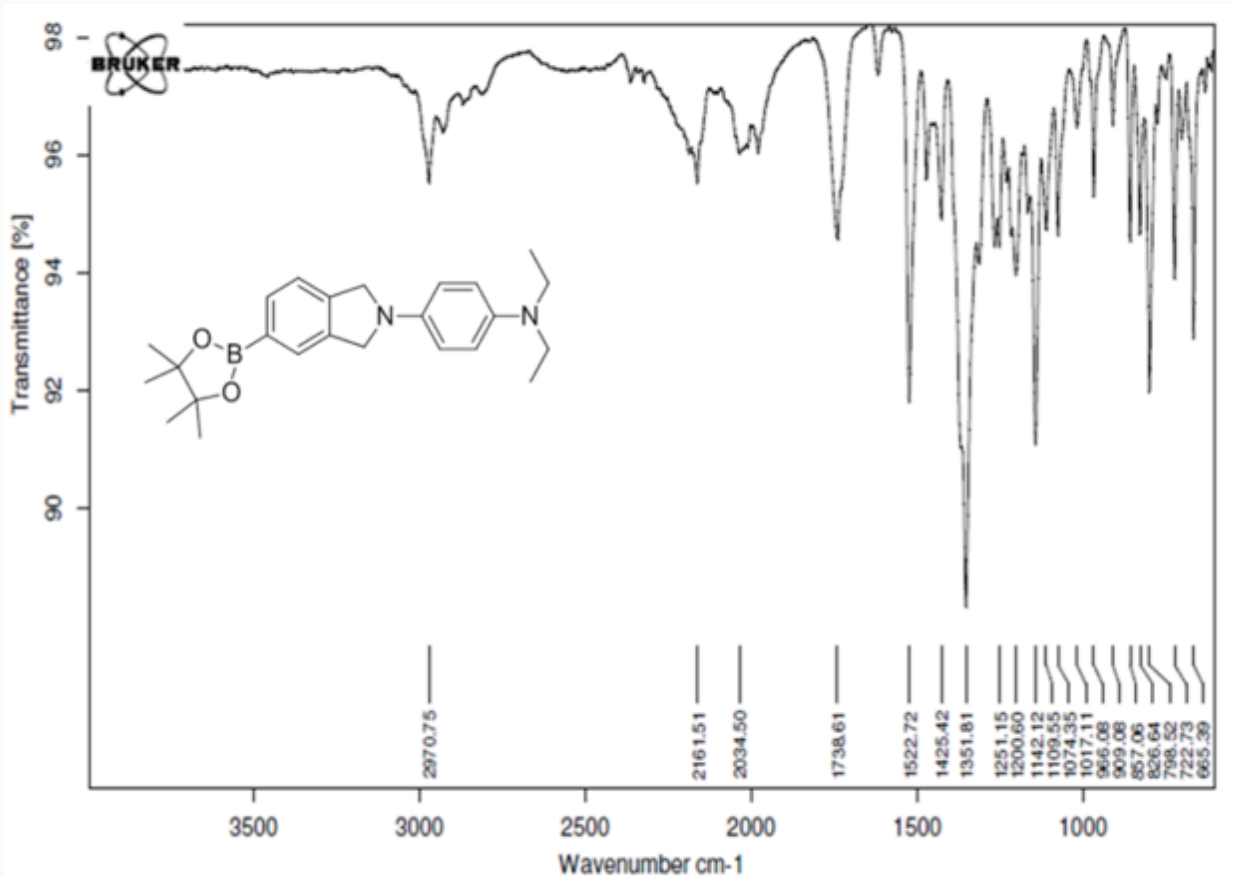

g. 2-Bromoanthraquinone

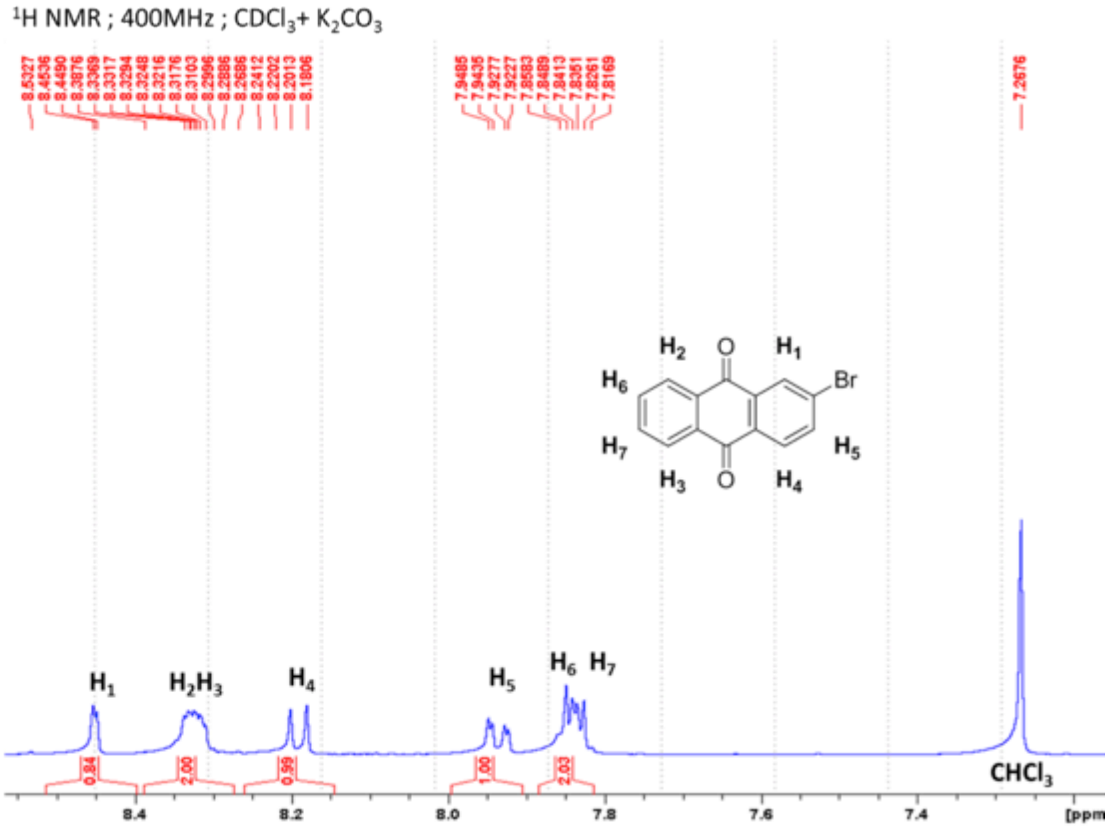

h. 2-Bromoanthracene (9) (mixture with anthracene)[S1]

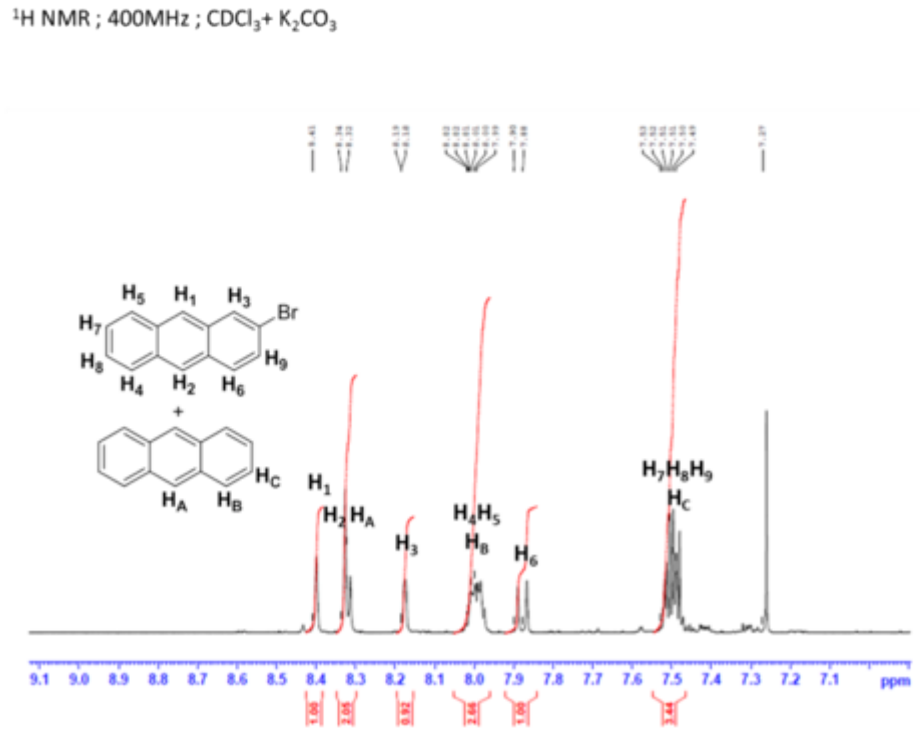

i. **2-Bromo-5,12-[1,2]benzenotetracene-6,11(5H,12H)-dione (mixture with 5,12-[1,2]benzenotetracene-6,11(5H,12H)-dione)**

^{1}H NMR ; 400MHz ; $CDCl_3$+ K_2CO_3

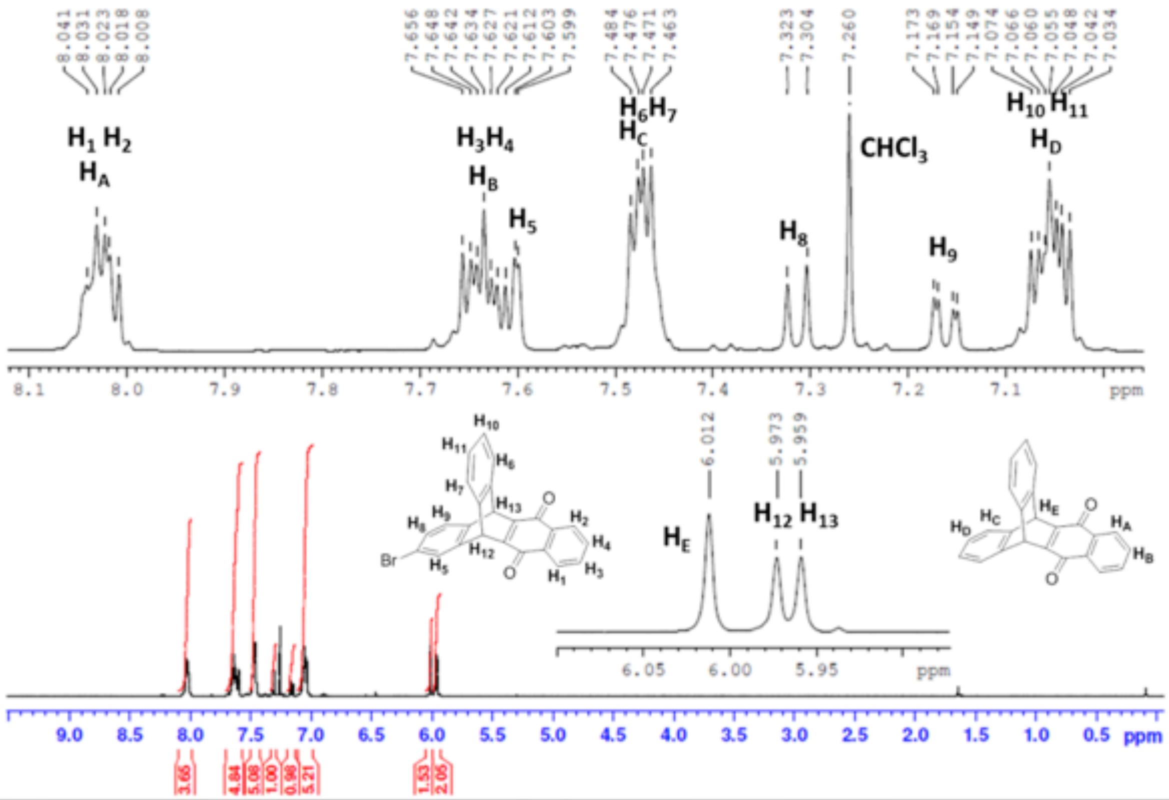

j. 2-(4,4,5,5-Tetramethyl-1,3,2-dioxaborolan-2-yl)-5,12-[1,2]benzenotetracene-6,11(5H,12H)-dione (10)

^{1}H NMR ; 400MHz ; $CDCl_3$+ K_2CO_3

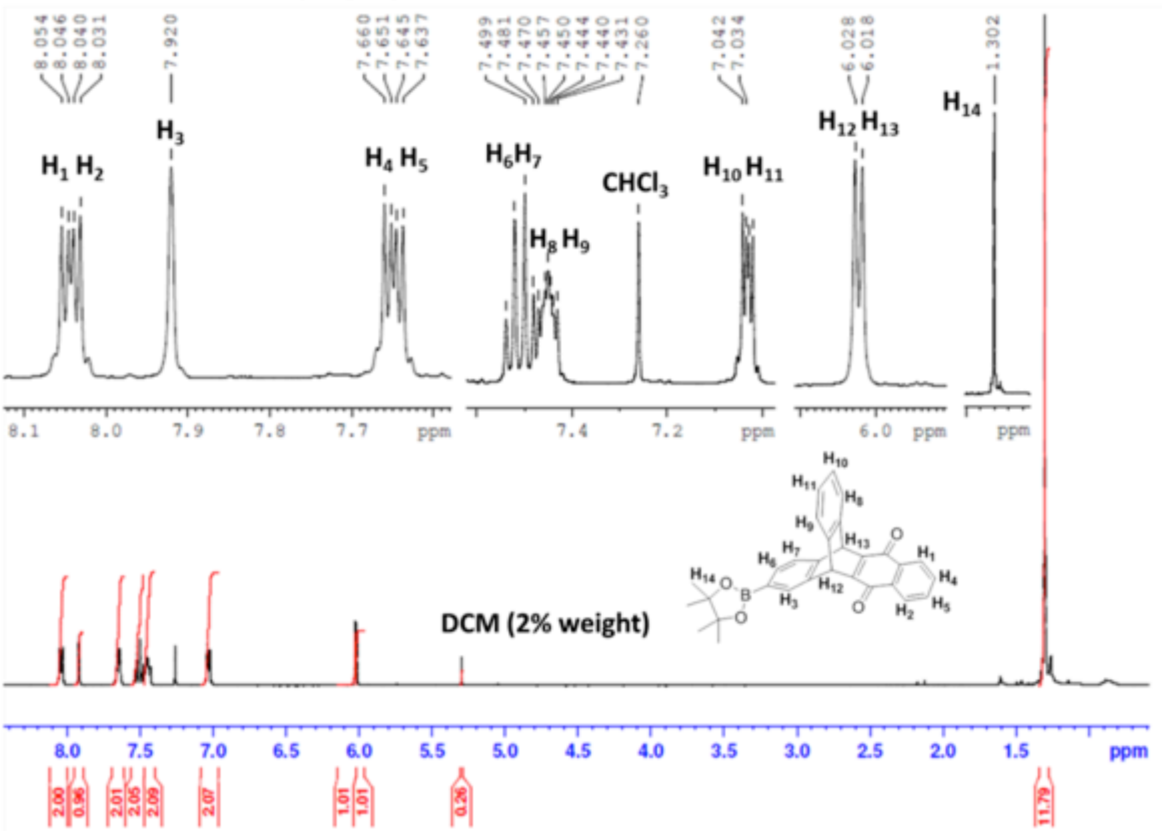

^{13}C NMR ; 100MHz ; $CDCl_3$+ K_2CO_3

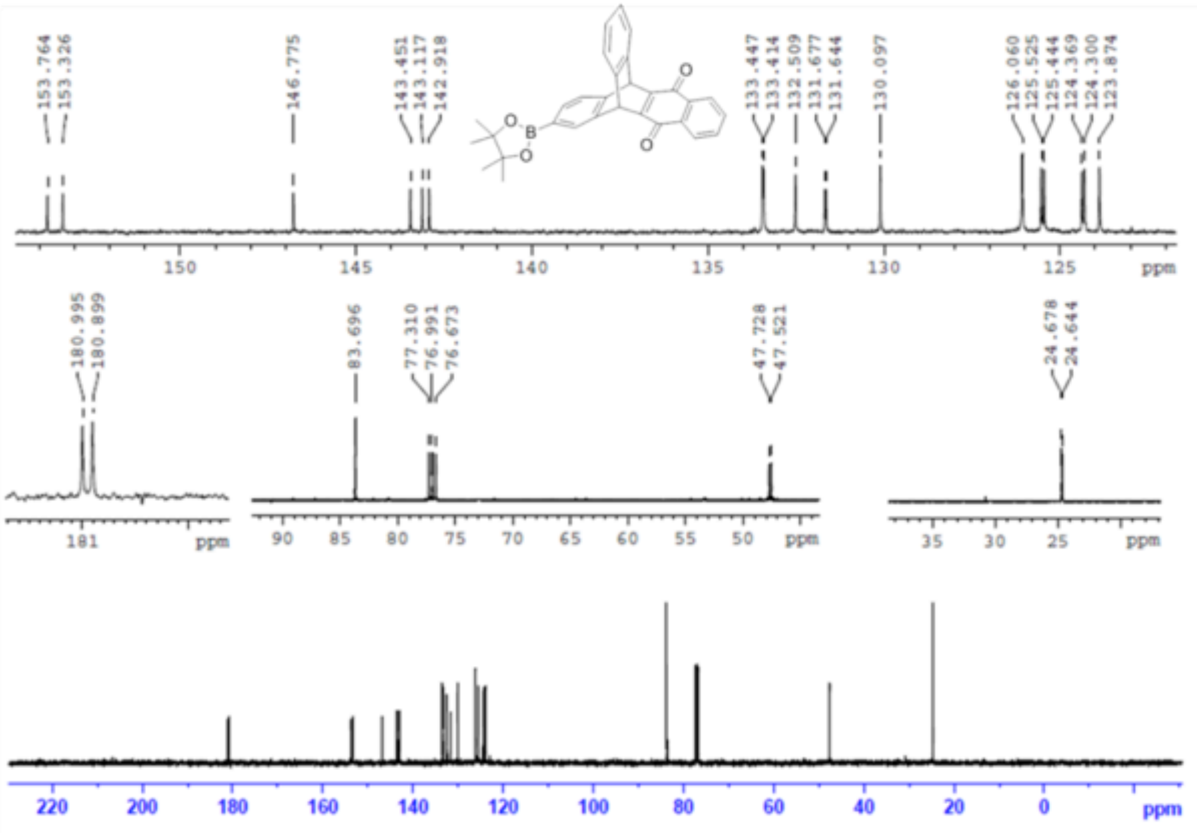

ESI⁺ HRMS

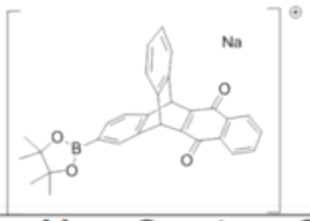

Mass Spectrum SmartFormula Report

Analysis Info

Analysis Name	\\Utof\data\Nov 13\ESI43670_20_01_14587.d	Acquisition Date	28/11/2013 09:26:46
Method	2.5min_cal_sample_pos_naf_05-08-13.m	Operator	Mass Spec
Sample Name	ESI43670	Instrument / Ser#	micrOTOF 92
Comment			

Acquisition Parameter

Source Type	ESI	Ion Polarity	Positive	Set Nebulizer	2.0 Bar
Focus	Not active			Set Dry Heater	180 °C
Scan Begin	100 m/z	Set Capillary	4500 V	Set Dry Gas	10.0 l/min
Scan End	1000 m/z	Set End Plate Offset	-500 V	Set Divert Valve	Source

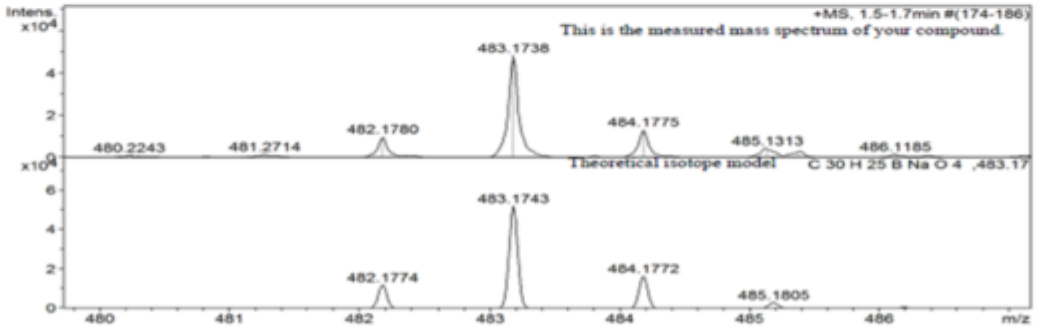

Meas. m/z	#	Formula	m/z	err [ppm]	Mean err [ppm]	rdb	e⁻ Conf	mSigma
483.1738	1	C 30 H 25 B Na O 4	483.1738	-0.0	6.4	18.5	even	26.32

Infra-red vibrational spectroscopy

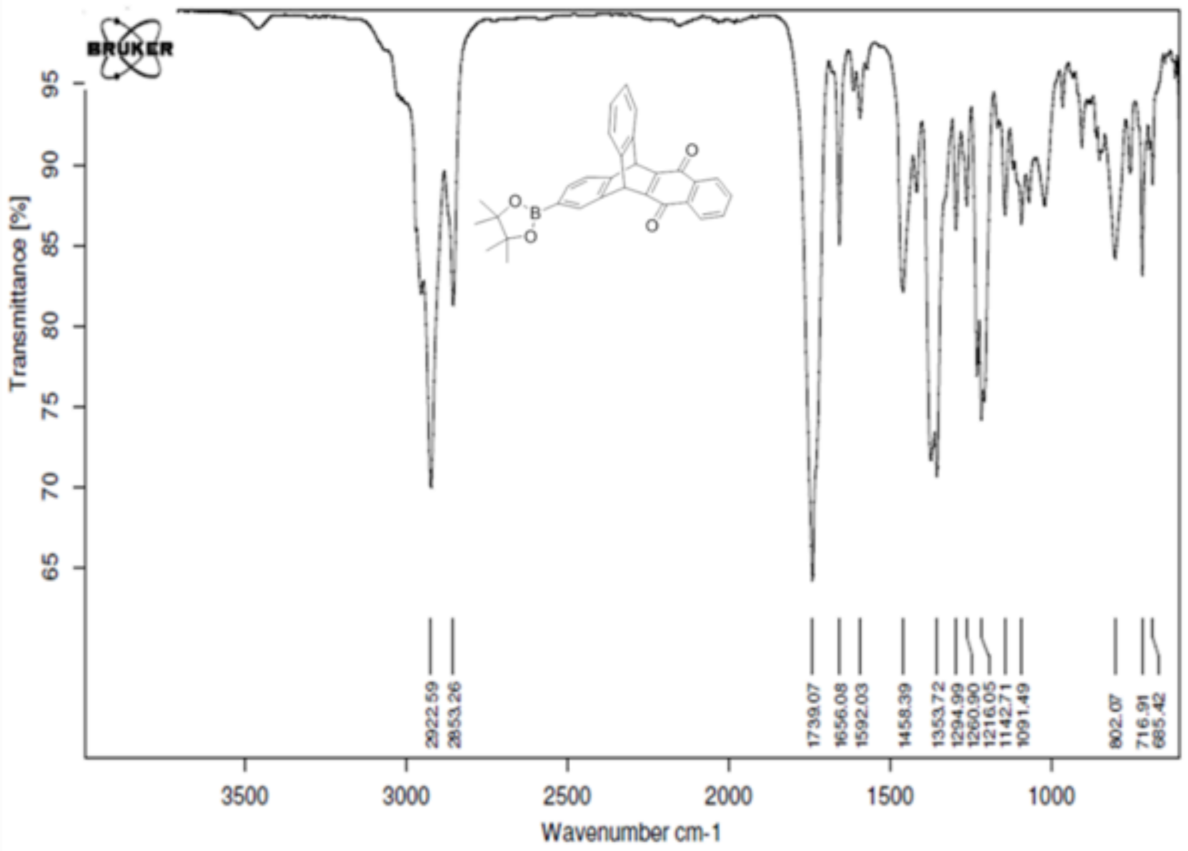

k. [5,15-Bis-(3,5-bis-*tert*-butylphenyl)-10-[5,12-[1,2]benzenotetracen-2-yl-6,11(5H,12H)-dione]-20-bromoporphinato]zinc(II) (12)

^{1}H NMR ; 400MHz ; $CDCl_3$+ 1% Pyridine-d_5

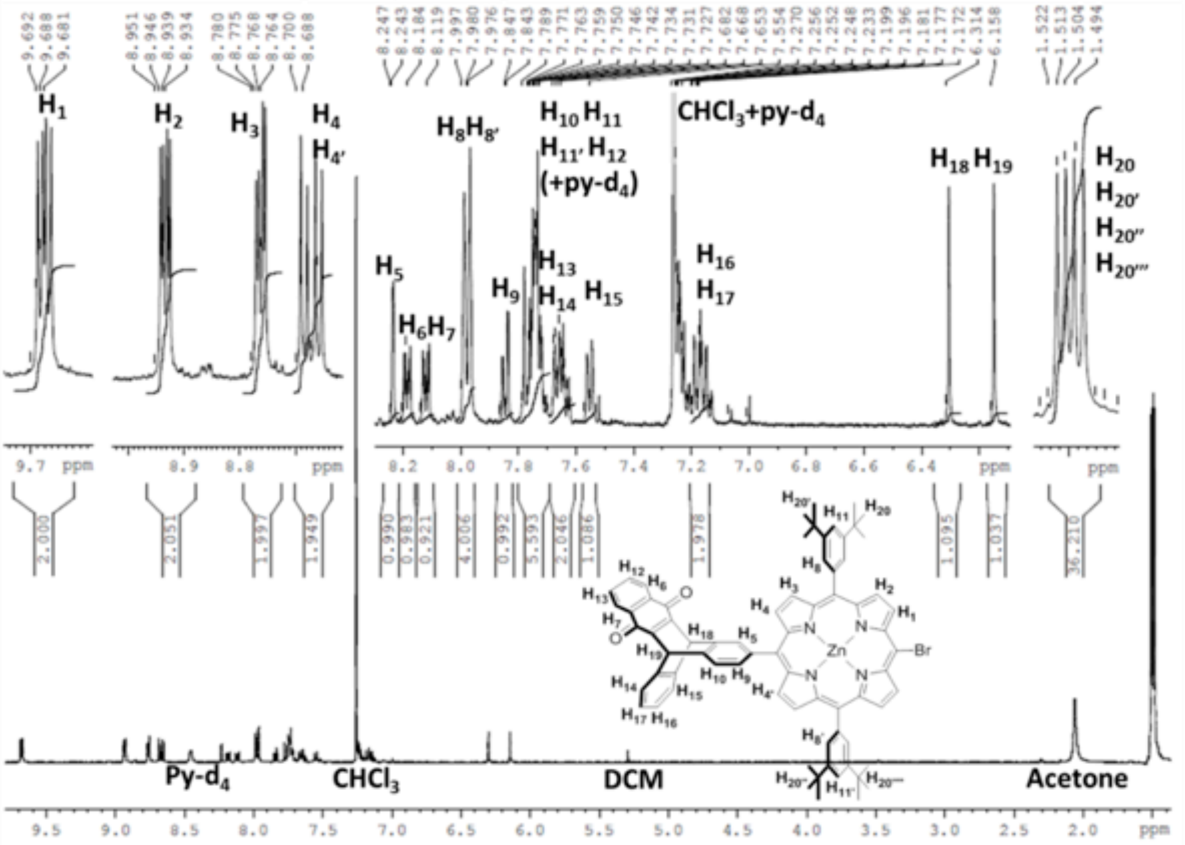

^{13}C NMR ; 100MHz ; $CDCl_3$+ 1% Pyridine-d_5

* For these carbons, the slow rotation around the porphyrin-acceptor bond breaks the perpendicular to porphyrin symmetry plan, causing dedoubling of the otherwise identical top and bottom part carbons of the porphyrin.

** Pyridine-d_5

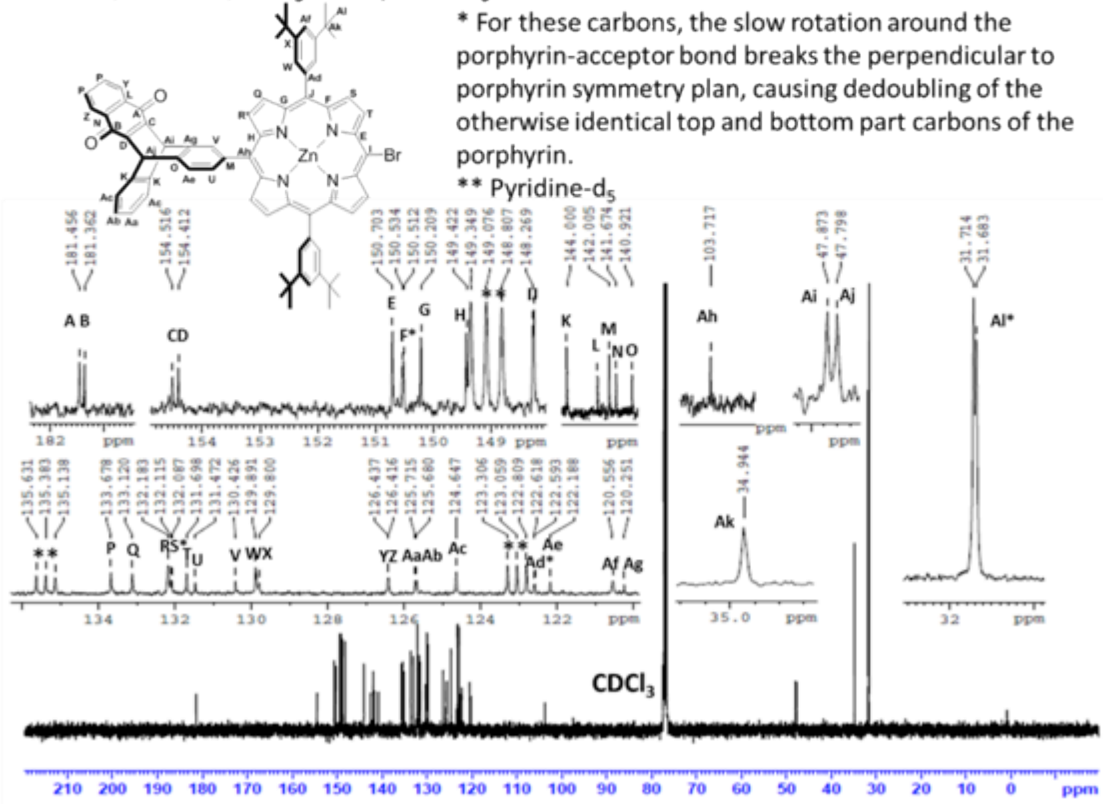

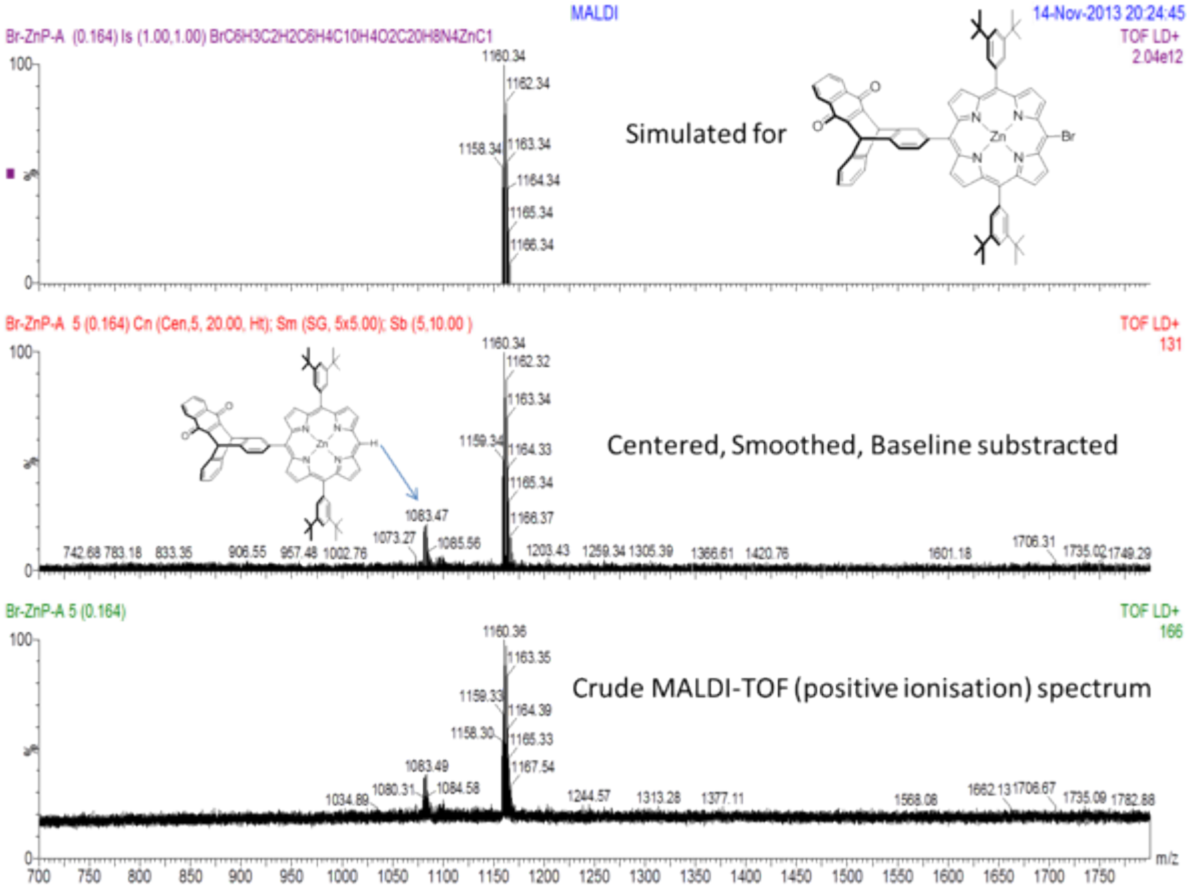

MALDI
14-Nov-2013 20:24:45
Br-ZnP-A (0.164) Is (1.00,1.00) BrC6H3C2H2C6H4C10H4O2C20H8N4ZnC1
TOF LD+
2.04e12
Simulated for
Br-ZnP-A 5 (0.164) Cn (Cen,5, 20.00, Ht); Sm (SG, 5x5.00); Sb (5,10.00)
TOF LD+
131
Centered, Smoothed, Baseline substracted
Br-ZnP-A 5 (0.164)
TOF LD+
166
Crude MALDI-TOF (positive ionisation) spectrum
m/z

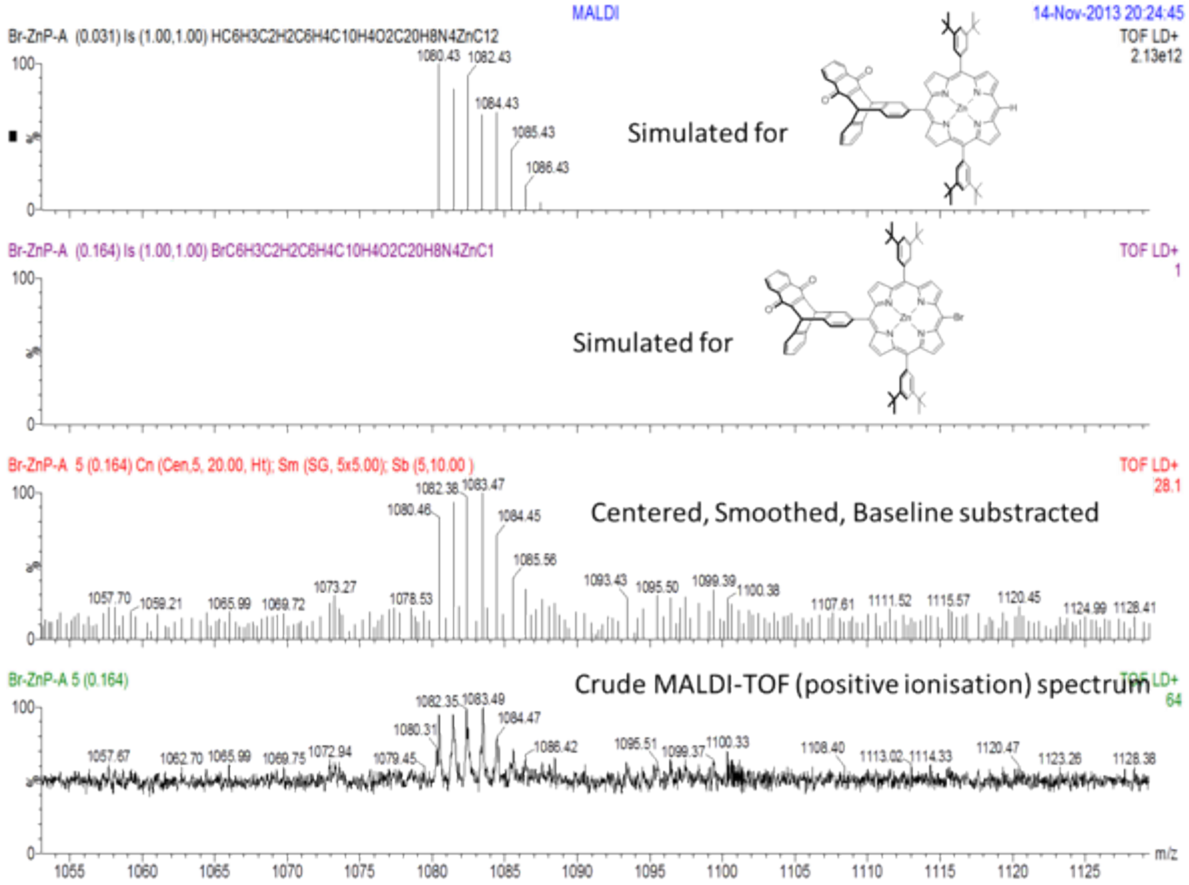

MALDI
14-Nov-2013 20:24:45
Br-ZnP-A (0.031) Is (1.00,1.00) HC6H3C2H2C6H4C10H4O2C20H8N4ZnC12
TOF LD+
2.13e12
Simulated for
Br-ZnP-A (0.164) Is (1.00,1.00) BrC6H3C2H2C6H4C10H4O2C20H8N4ZnC1
TOF LD+
1
Simulated for
Br-ZnP-A 5 (0.164) Cn (Cen,5, 20.00, Ht); Sm (SG, 5x5.00); Sb (5,10.00)
TOF LD+
28.1
Centered, Smoothed, Baseline substracted
Br-ZnP-A 5 (0.164)
Crude MALDI-TOF (positive ionisation) spectrum
TOF LD+
64
m/z

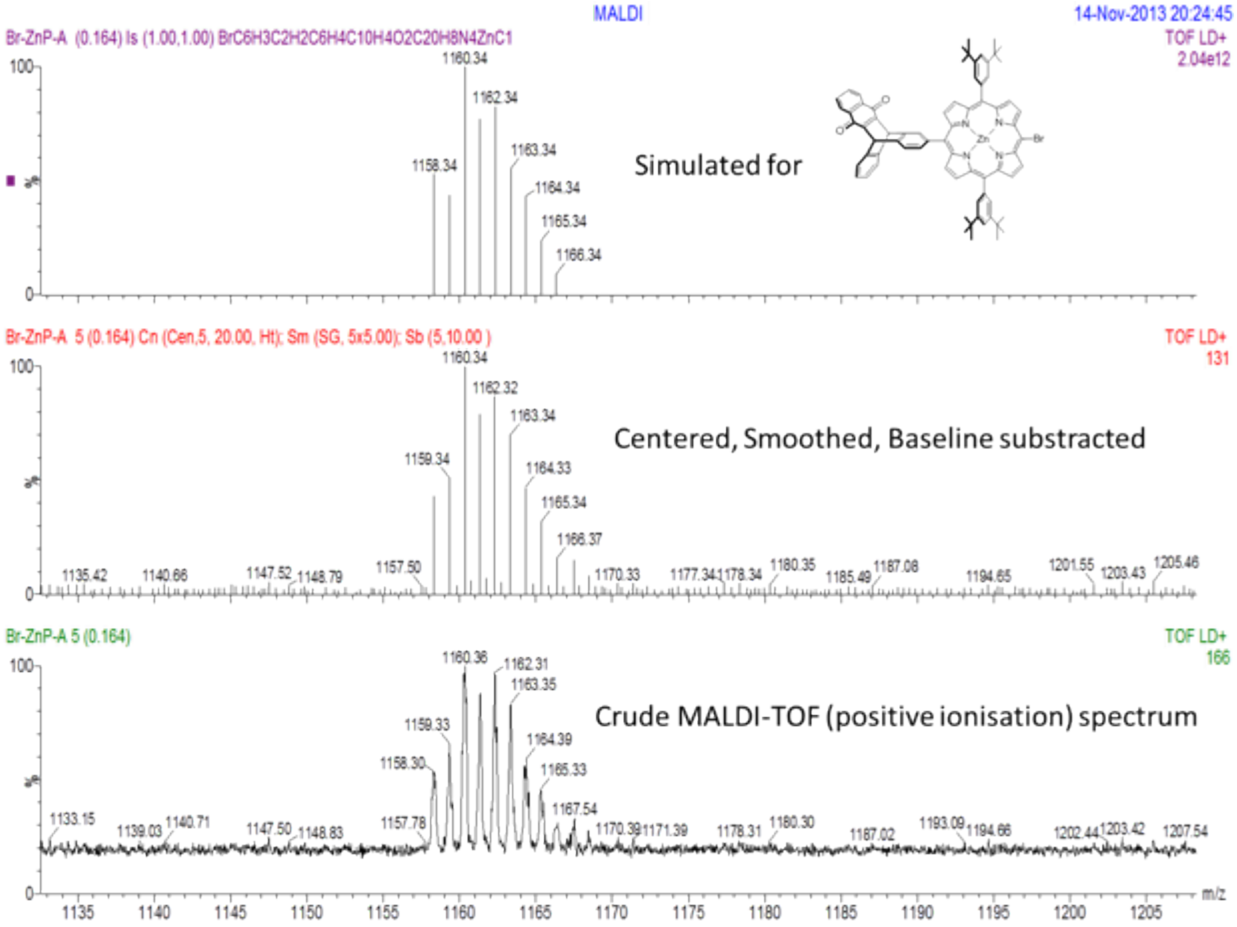

Infra-red vibrational spectroscopy

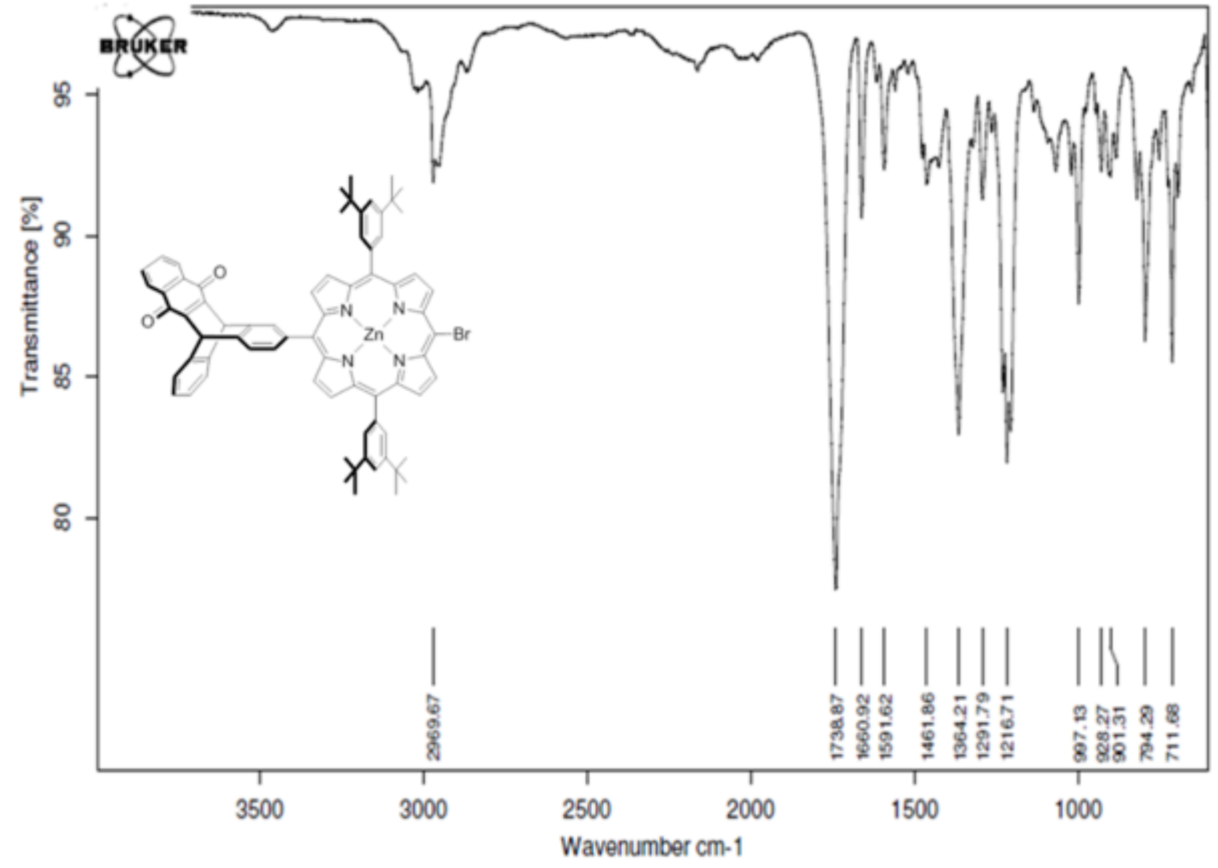

I. [5,15-Bis-(3,5-bis-*tert*-butylphenyl)-10-[5,12-[1,2]benzenotetracen-2-yl-6,11(5H,12H)-dione]-20-(4-(diethylamino)phenyl)-*4H*-isoindolin-2-yl)porphinato]zinc(II) (TNQ-ZnP$_{Ar}$-TAPD)

^{1}H NMR ; 400MHz ; $CDCl_3$+ pyridine-d_5 + K_2CO_3

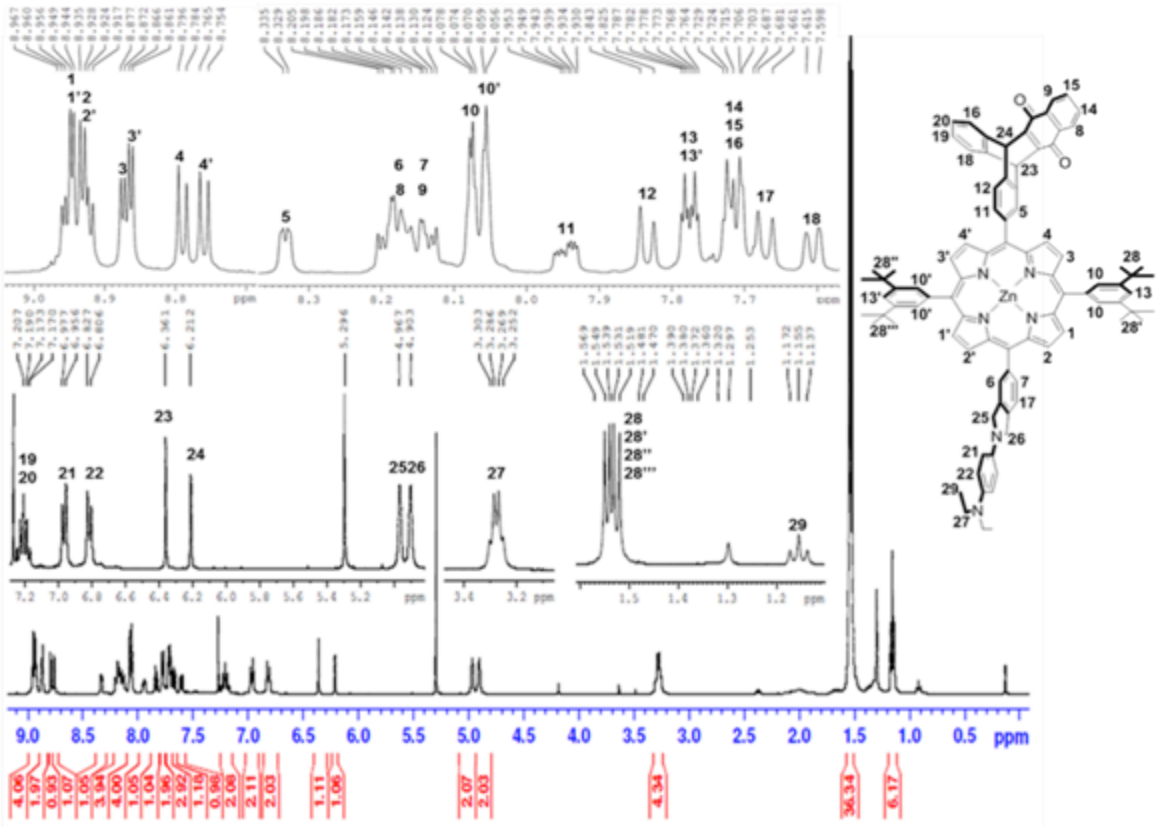

^{13}C NMR ; 125MHz ; $CDCl_3$+ 1% Pyridine-d_5

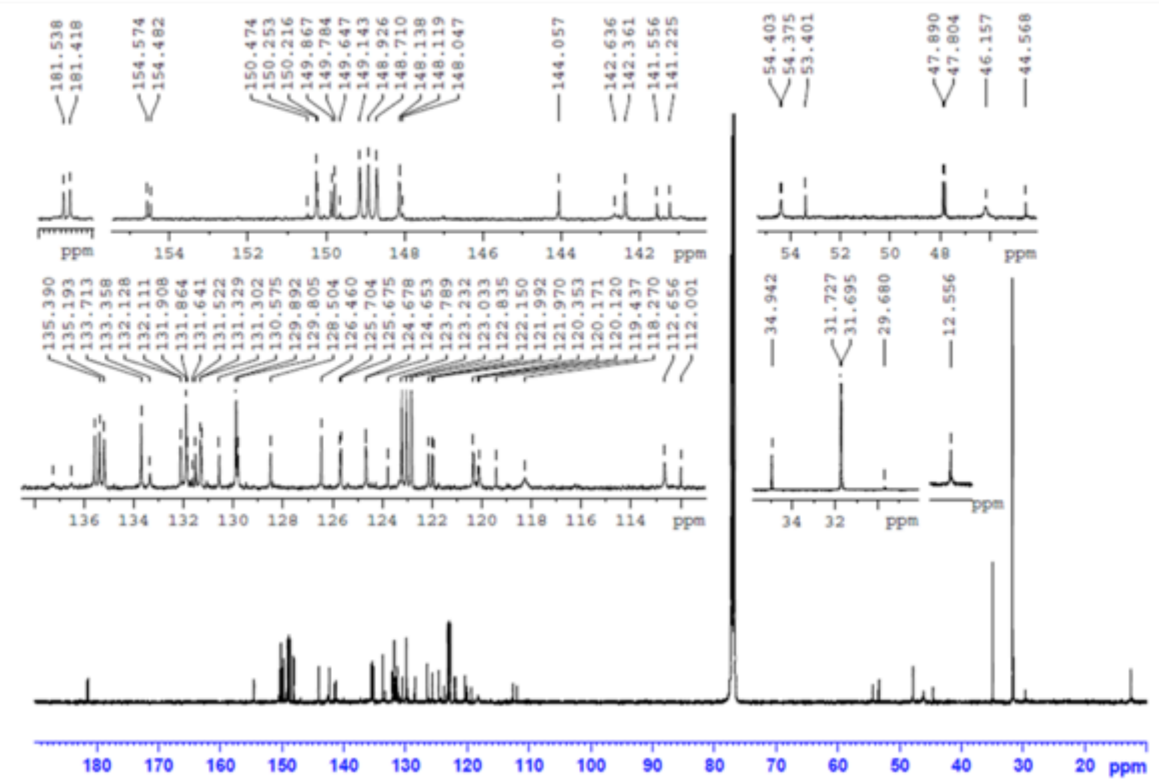

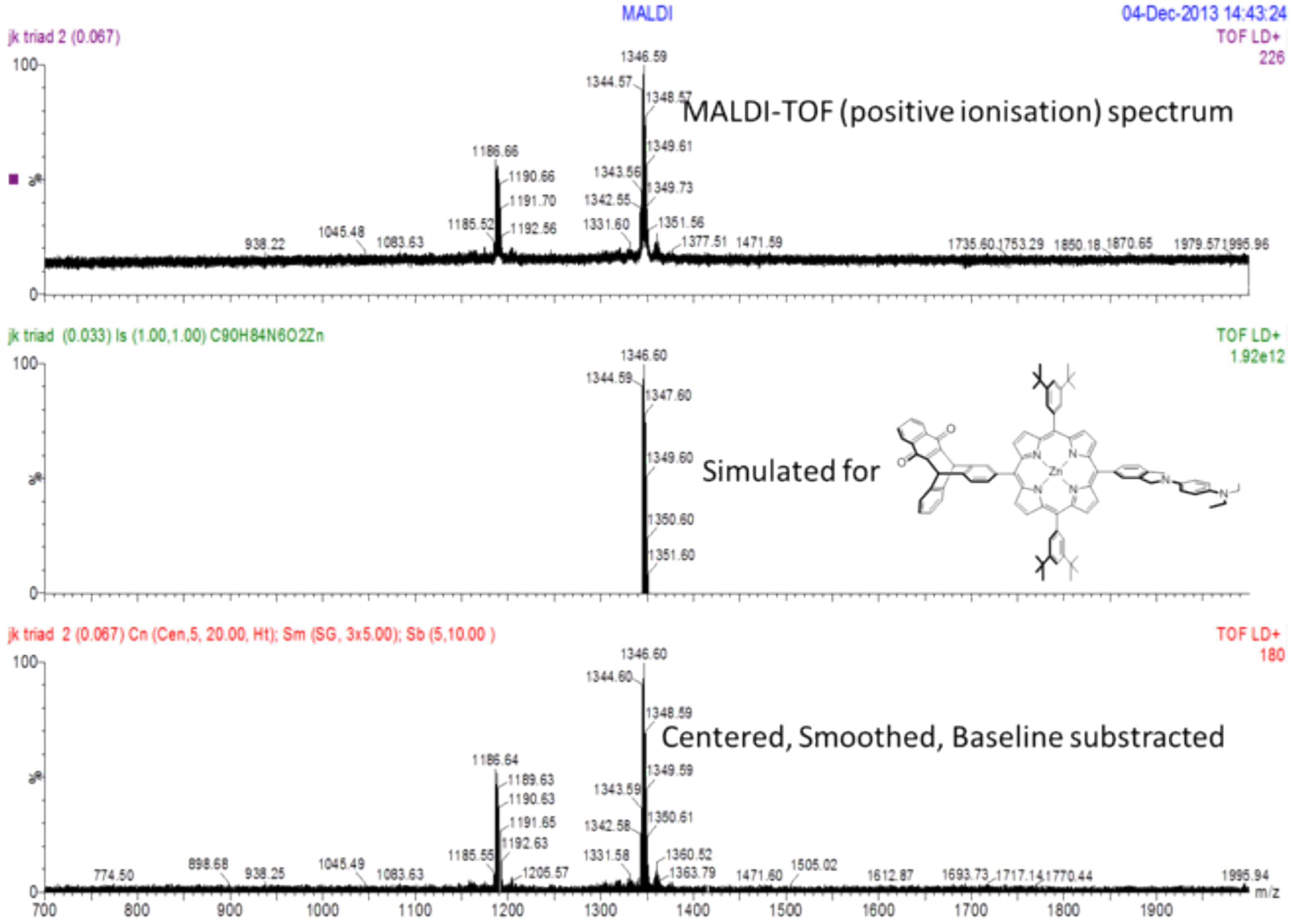

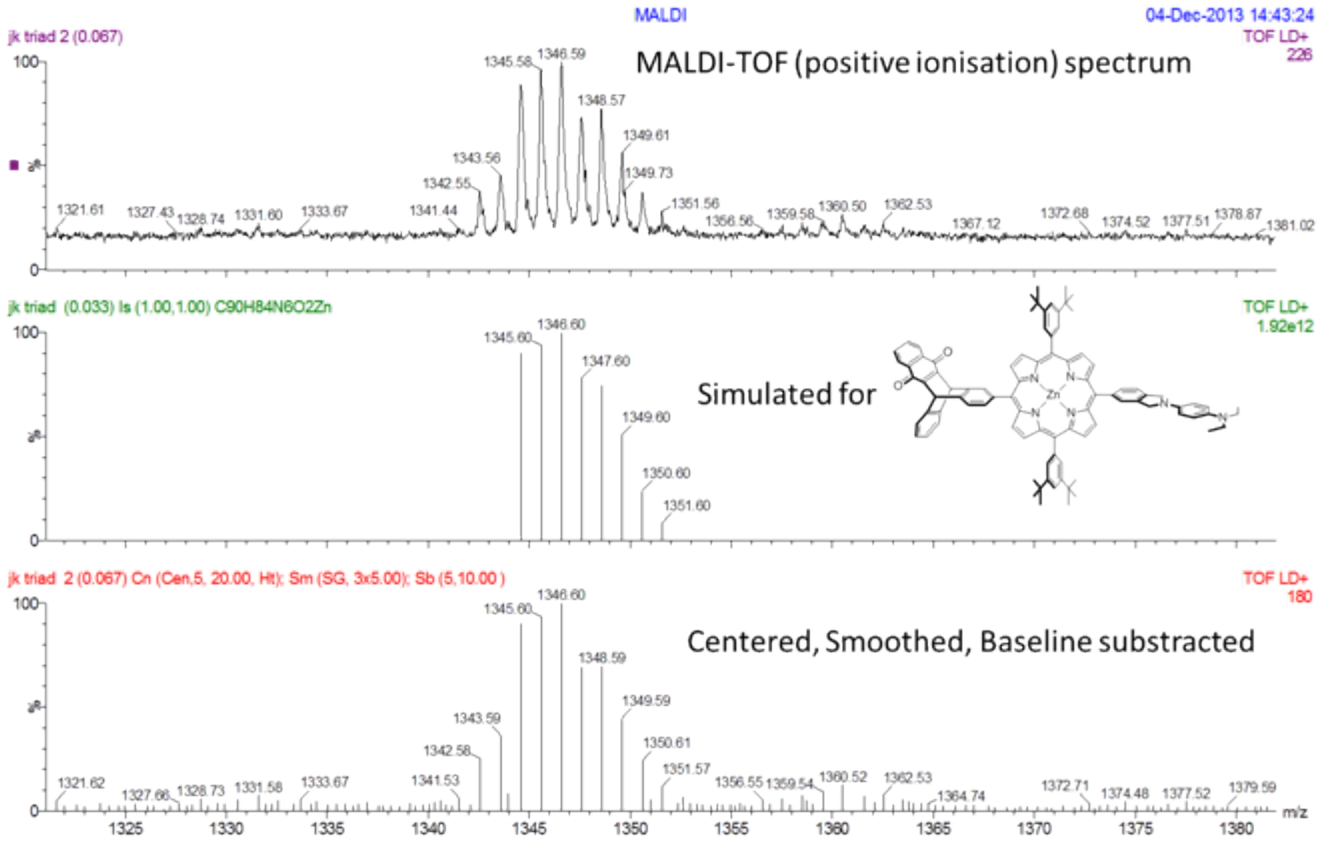

Infra-red vibrational spectroscopy

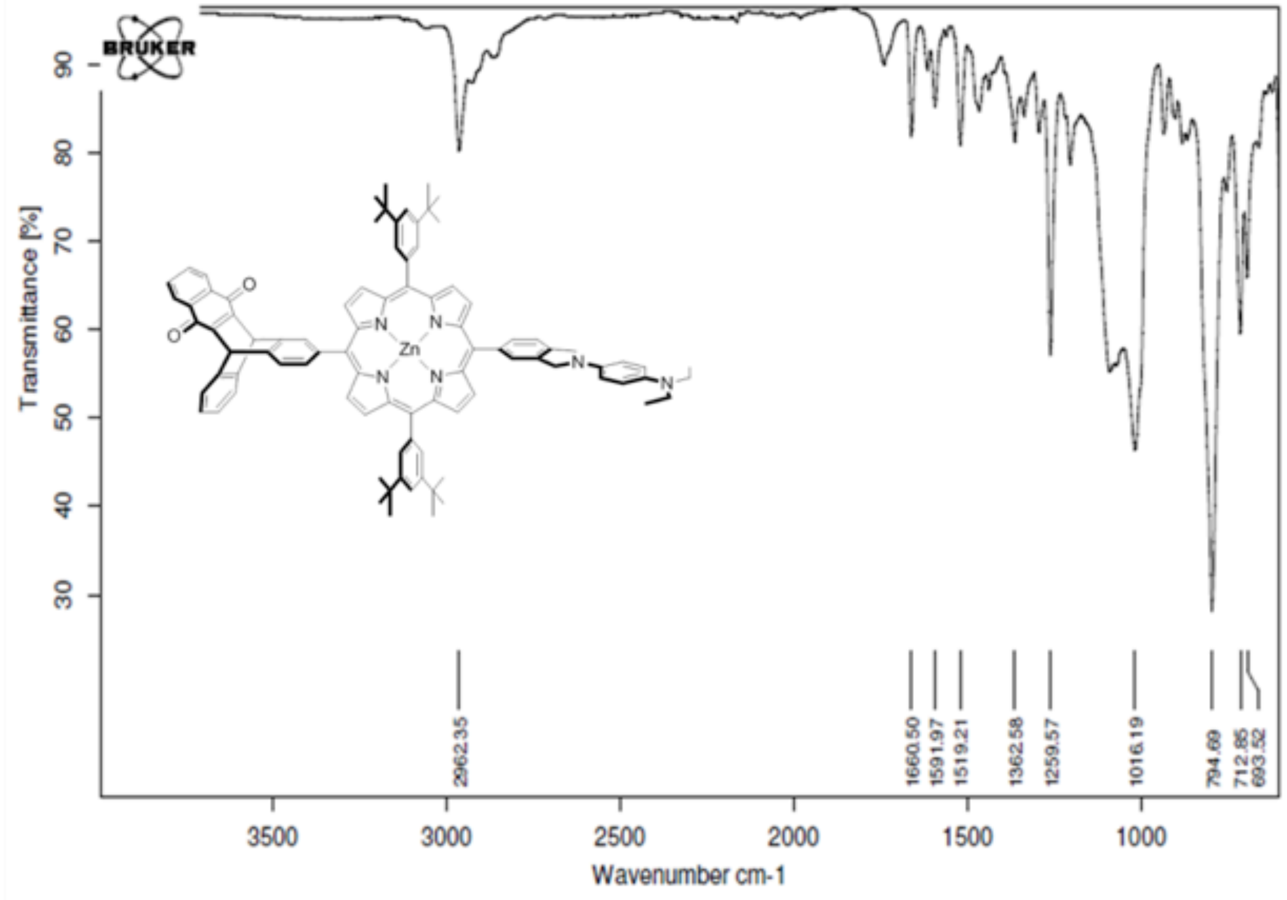

COSY (^1H-^1H) 400 MHz, $CDCl_3$+ 1% Py-d_5

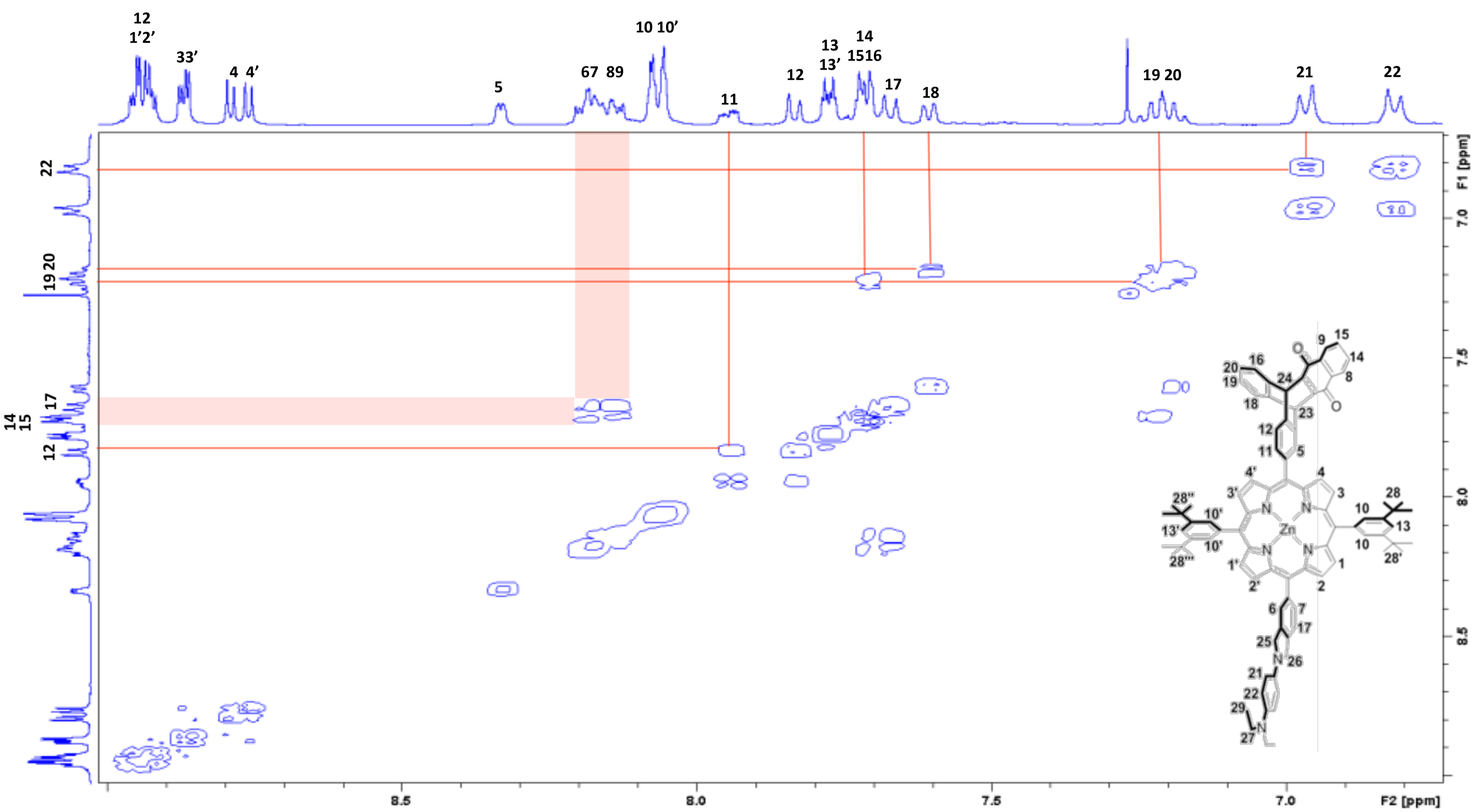

HSQC (^{1}H-^{13}C) 400 MHz, $CDCl_3$+ 1% Py-d_5

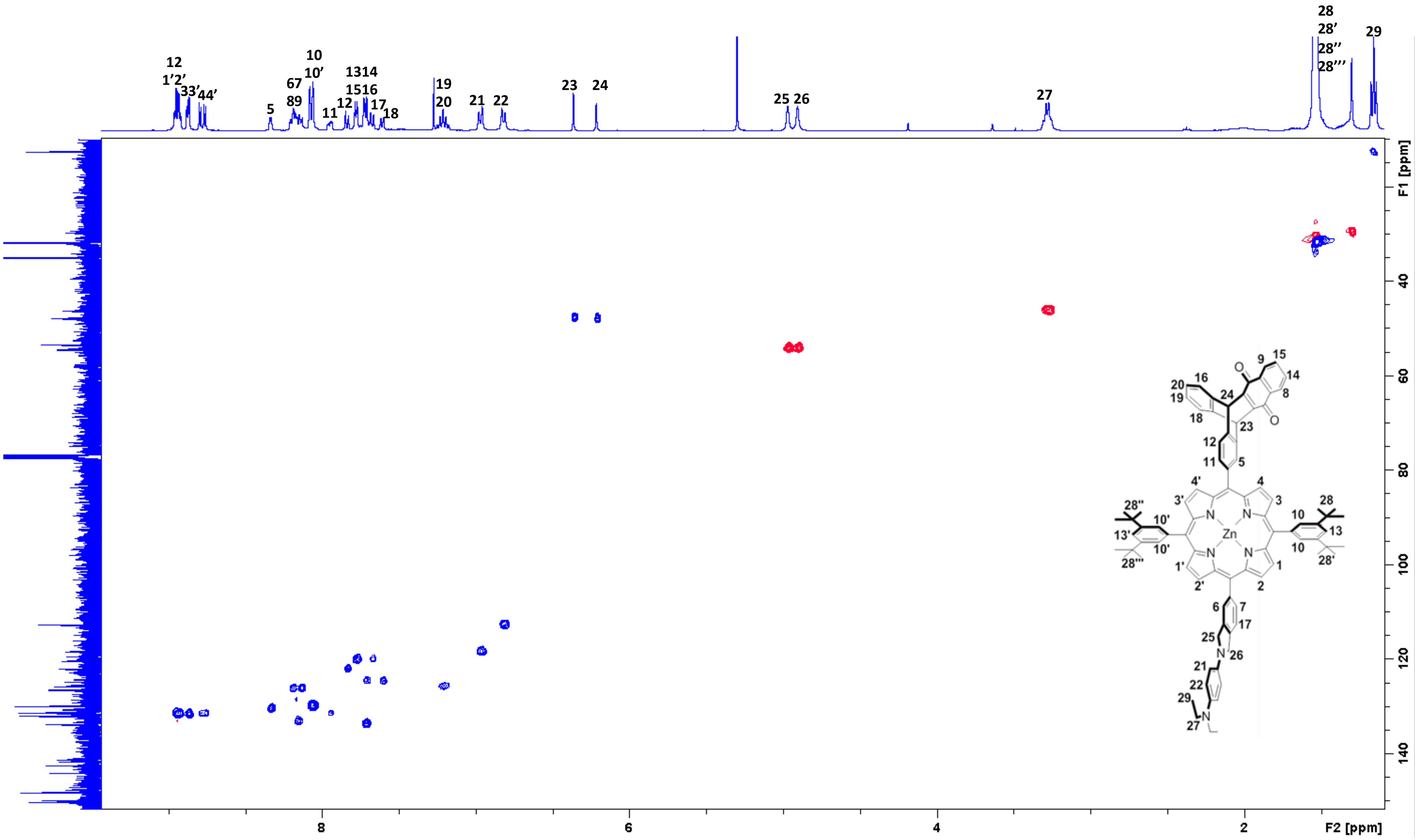

HMBC (^{1}H-^{13}C) 400 MHz, $CDCl_3$+ 1% Py-d_5

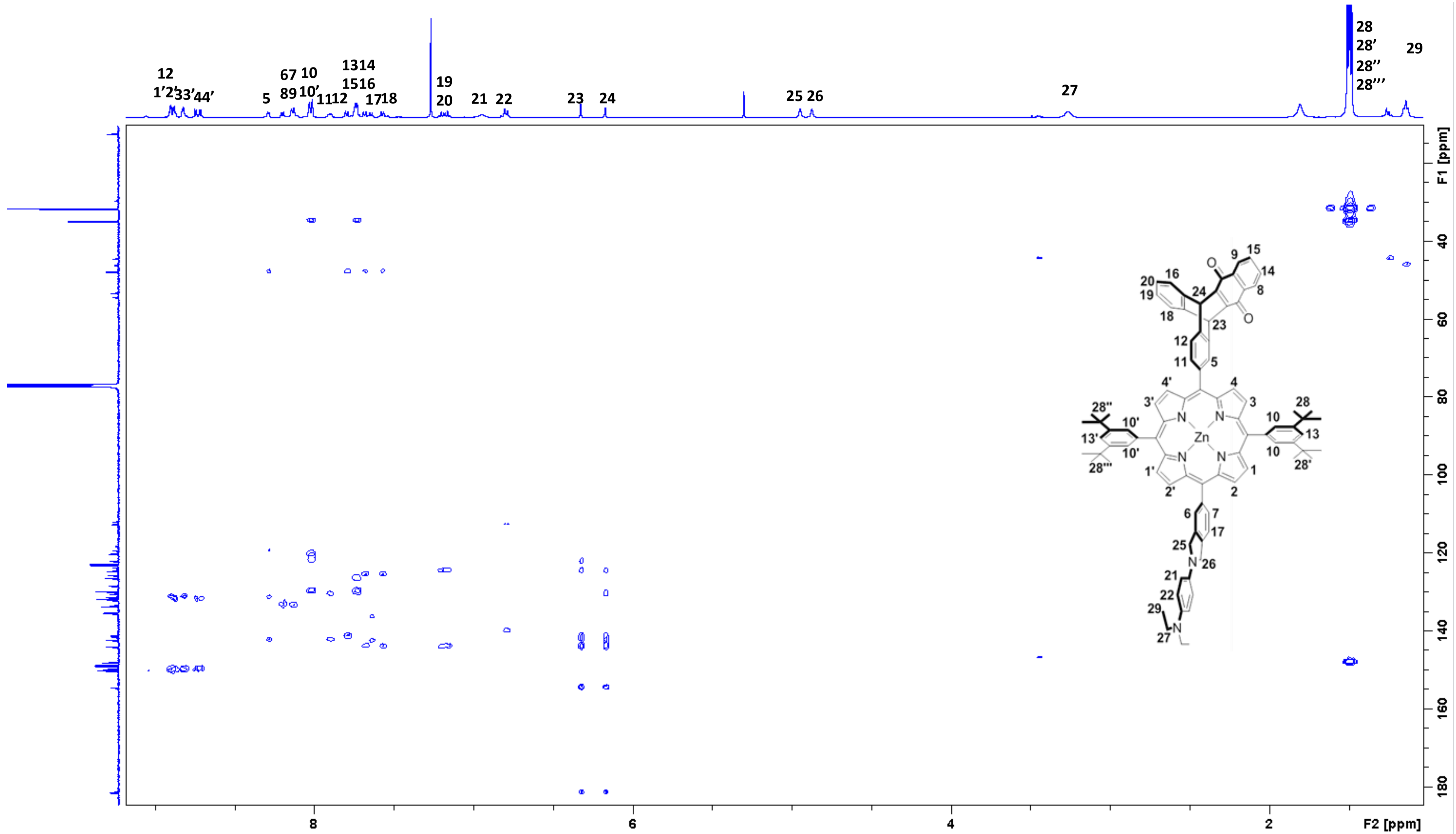

Steady-state absorption spectroscopy of the triad (298K, solvent MTHF)

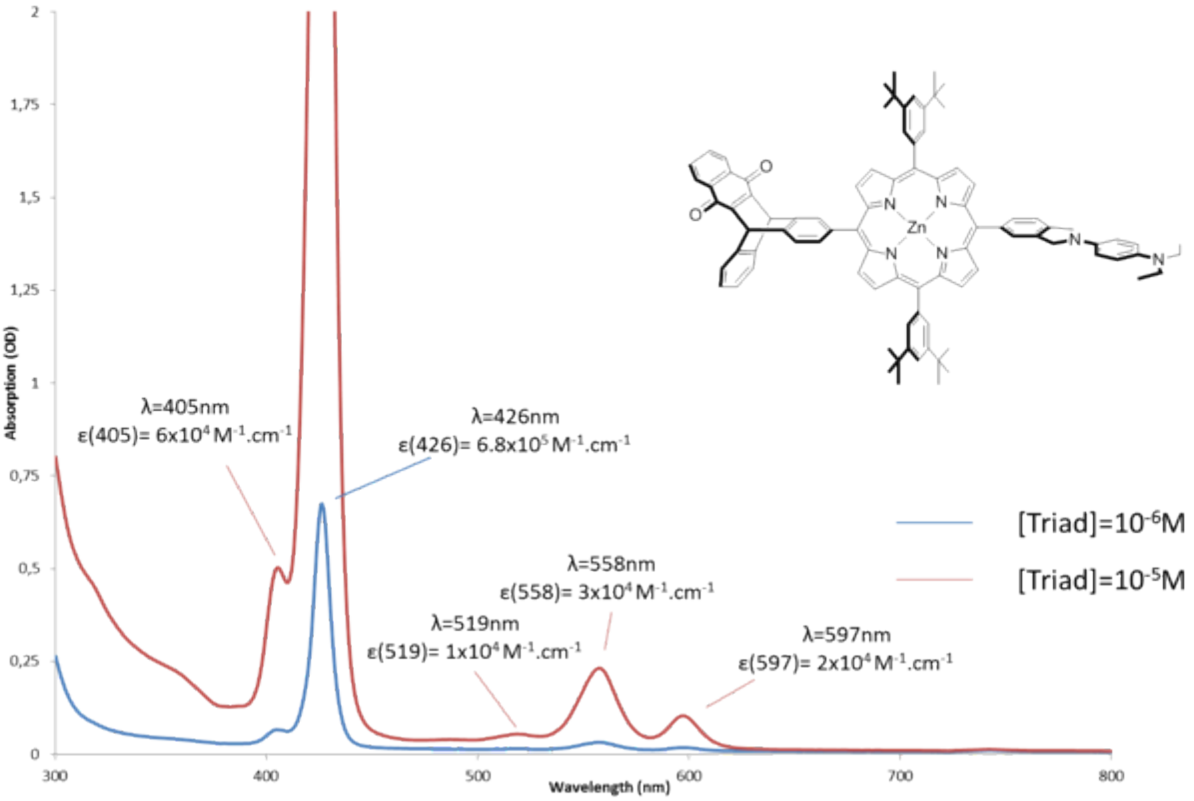

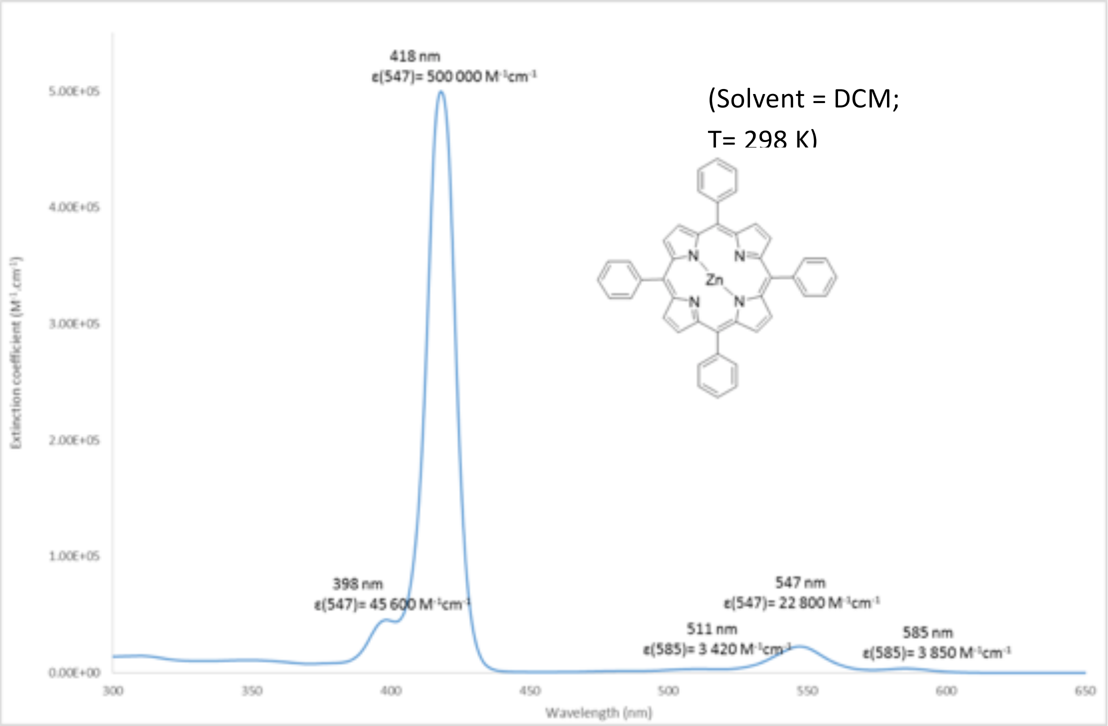

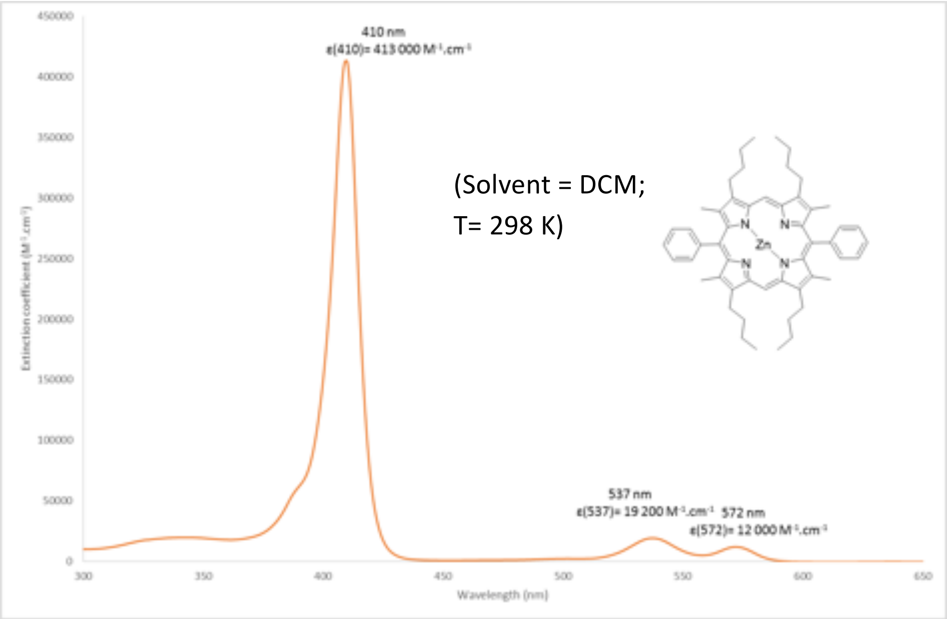
410 nm
ε(410)= 413 000 M⁻¹.cm⁻¹
(Solvent = DCM;
T= 298 K)
537 nm
ε(537)= 19 200 M⁻¹.cm⁻¹
572 nm
ε(572)= 12 000 M⁻¹.cm⁻¹
Extinction coefficient (M⁻¹.cm⁻¹)
Wavelength (nm)

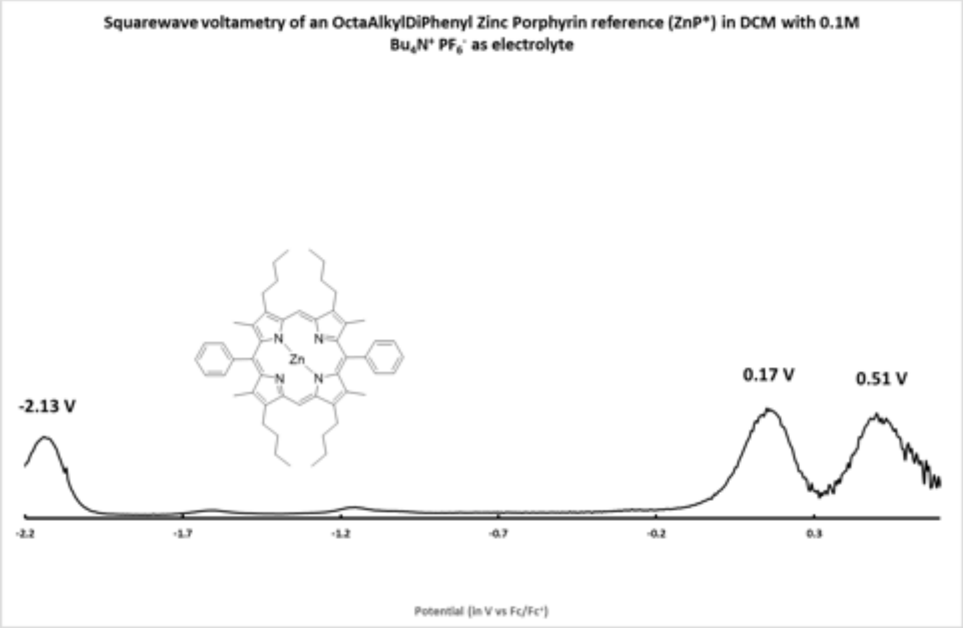
Squarewave voltametry of an OctaAlkylDiPhenyl Zinc Porphyrin reference (ZnP*) in DCM with 0.1M Bu4N+ PF6- as electrolyte
-2.13 V
0.17 V
0.51 V
Potential (in V vs Fc/Fc+)

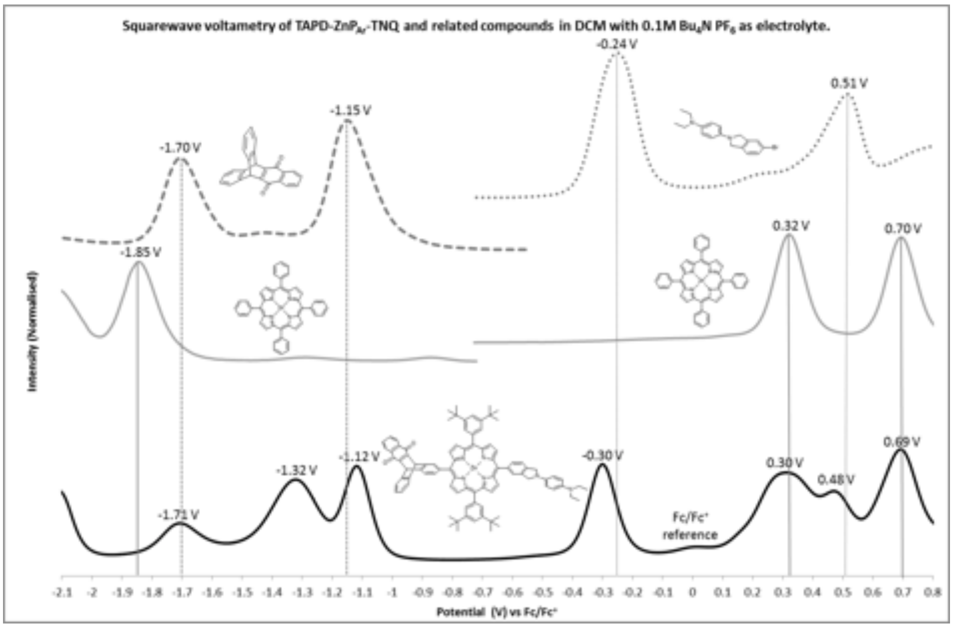
Squarewave voltametry of TAPD-ZnPAr-TNQ and related compounds in DCM with 0.1M Bu4N PF6 as electrolyte.
-0.24 V
0.51 V
-1.15 V
-1.70 V
-1.85 V
0.32 V
0.70 V
-1.32 V
-1.12 V
-1.71 V
-0.30 V
0.30 V
0.48 V
0.69 V
Fc/Fc+ reference
Intensity (Normalised)
Potential (V) vs Fc/Fc+

m. [5,15-Bis-(3,5-bis-*tert*-butyl-phenyl)-10-(4,4,5,5-tetramethyl-1,3,2-dioxaborolan-2-yl)-20-(4-benzaldehyde)-porphyrinato]zinc(II) (13)

^{1}H NMR ; 200MHz ; $CDCl_3$+ pyridine-d_5 + K_2CO_3

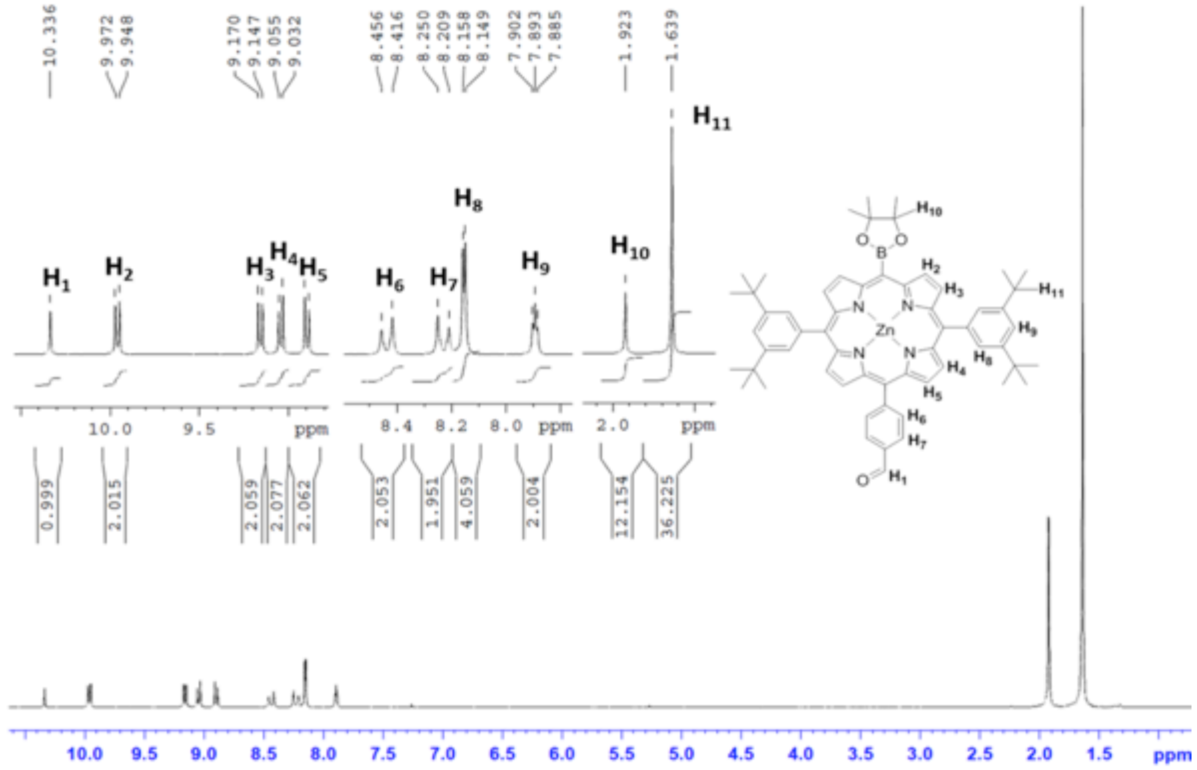

^{13}C NMR ; 100MHz ; $CDCl_3$+ 1% Pyridine-d_5 + K_2CO_3

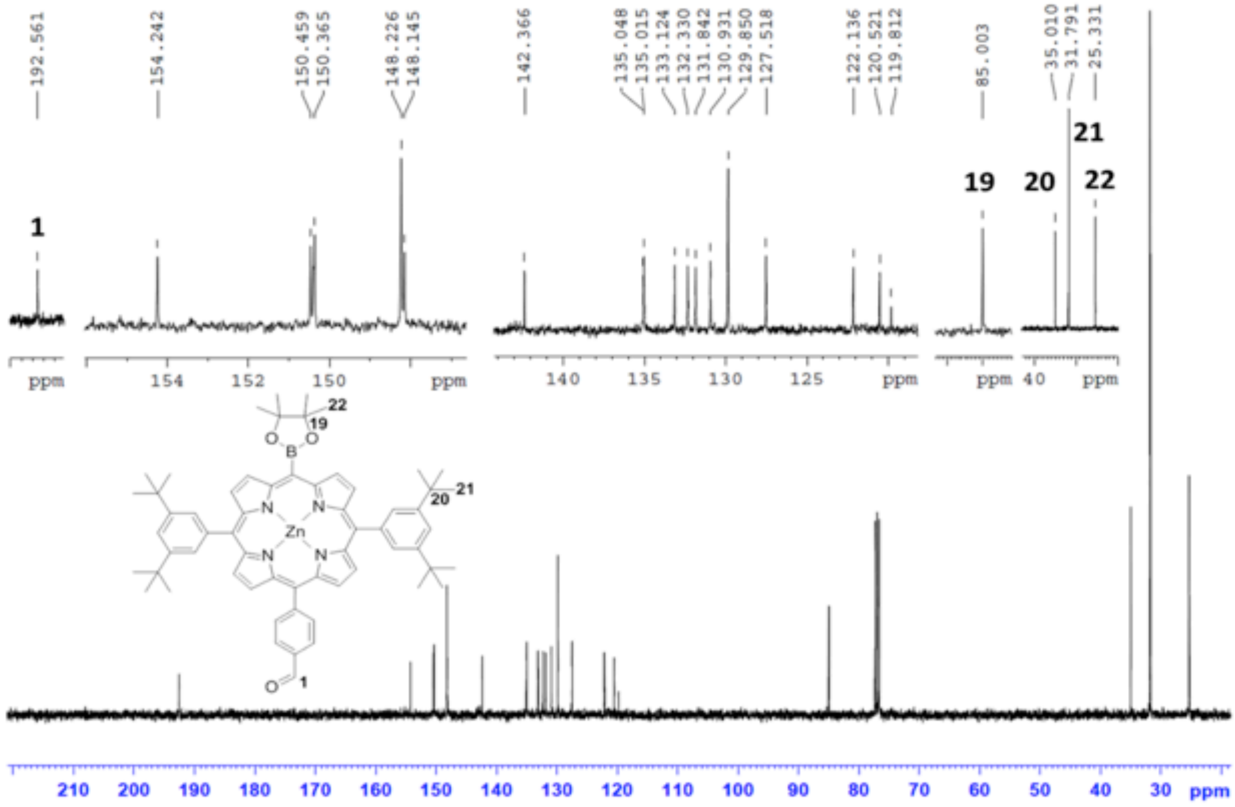

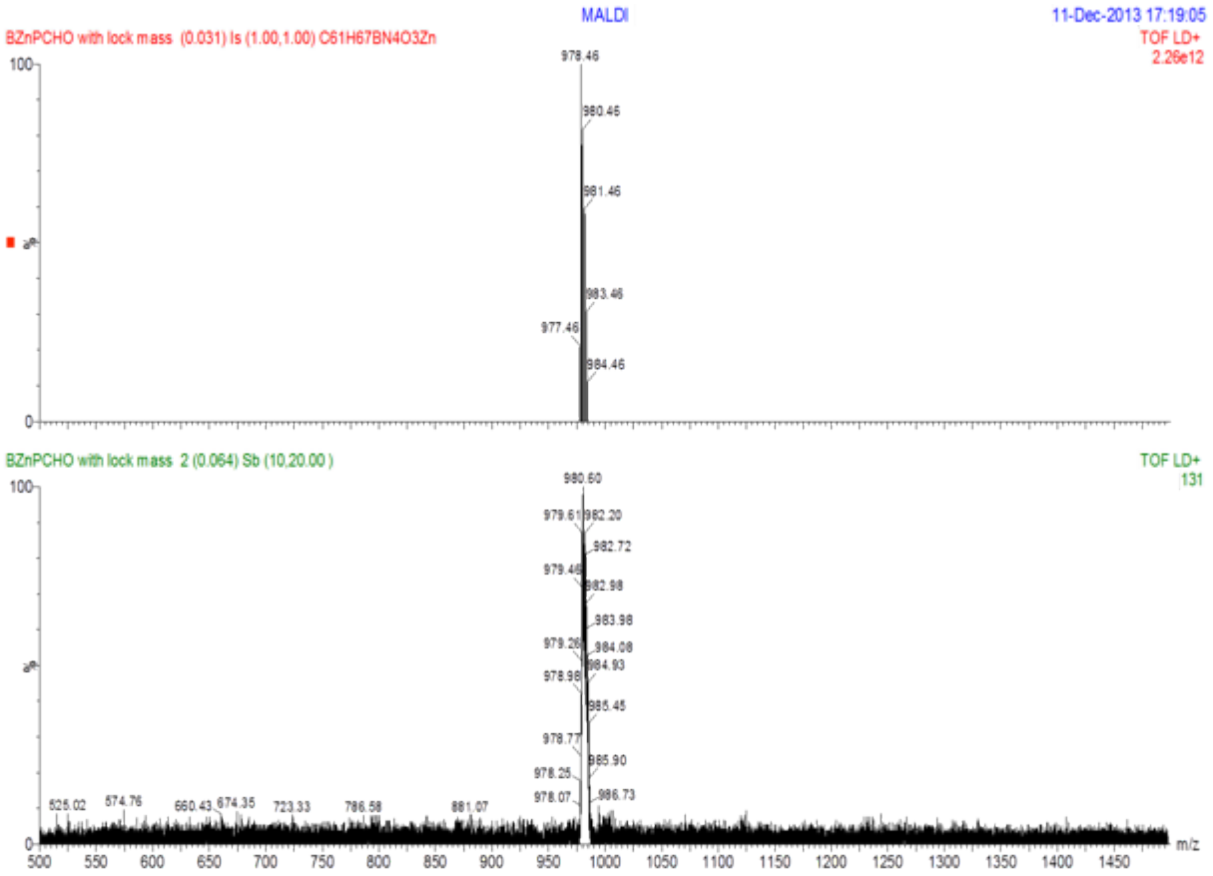

n. **[5,15-Bis-(3,5-bis-*tert*-butylphenyl)-10-(4-caboxyphenyl)-20-(4-(diethylamino)phenyl)-4H-isoindolin-2-yl)porphinato]zinc(II) (14):**

^{1}H NMR ; 400MHz ; $CDCl_3$+ 1% Pyridine-d_5

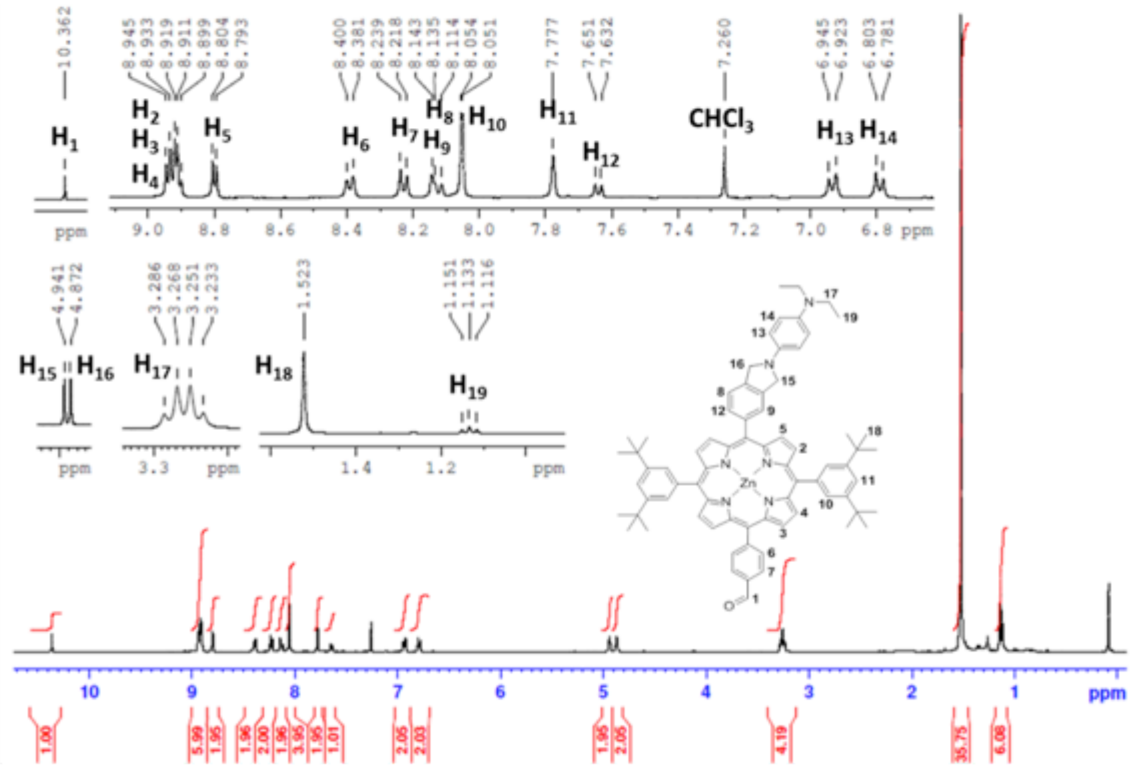

^{13}C NMR ; 100MHz ; $CDCl_3$+ 1% Pyridine-d_5 + K_2CO_3

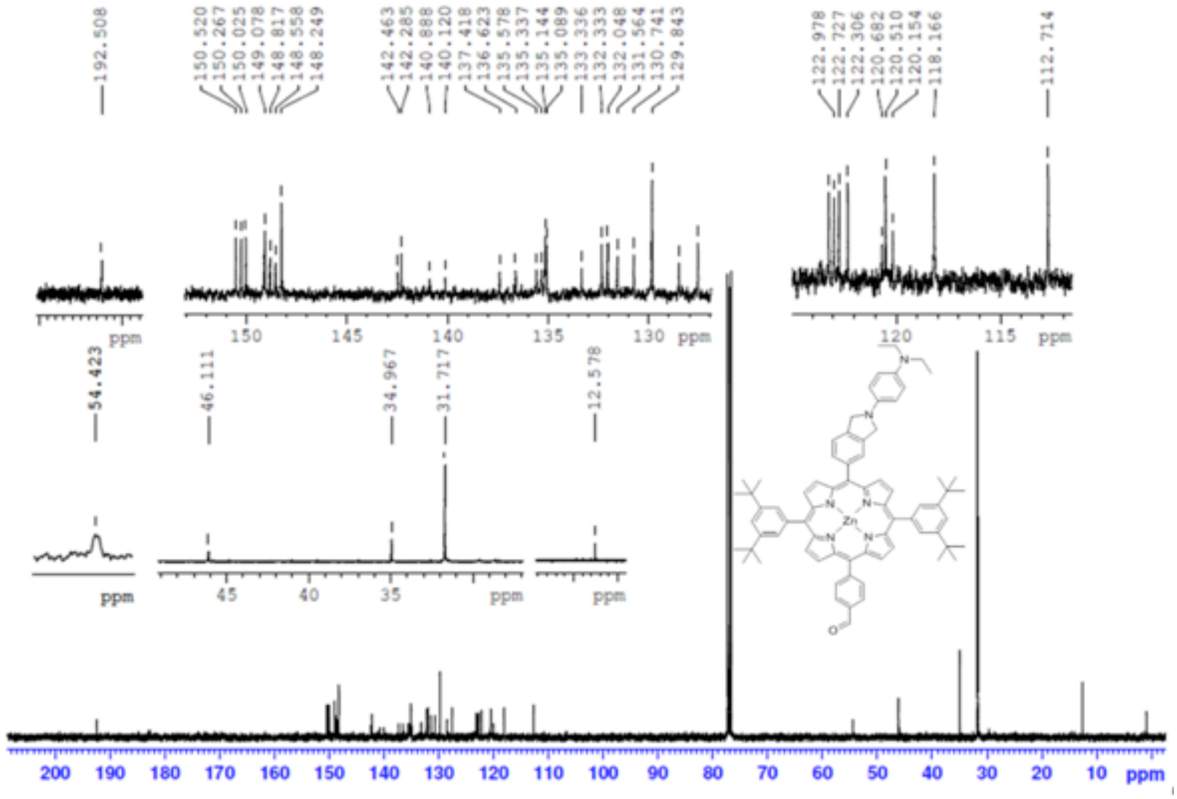

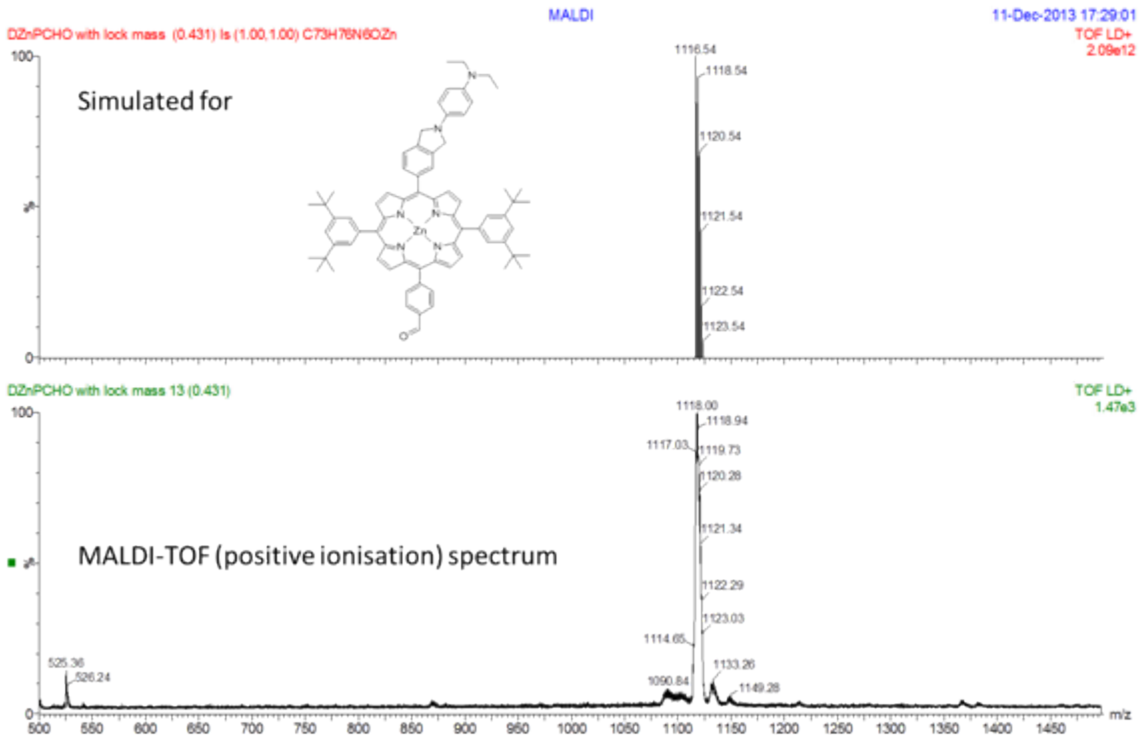

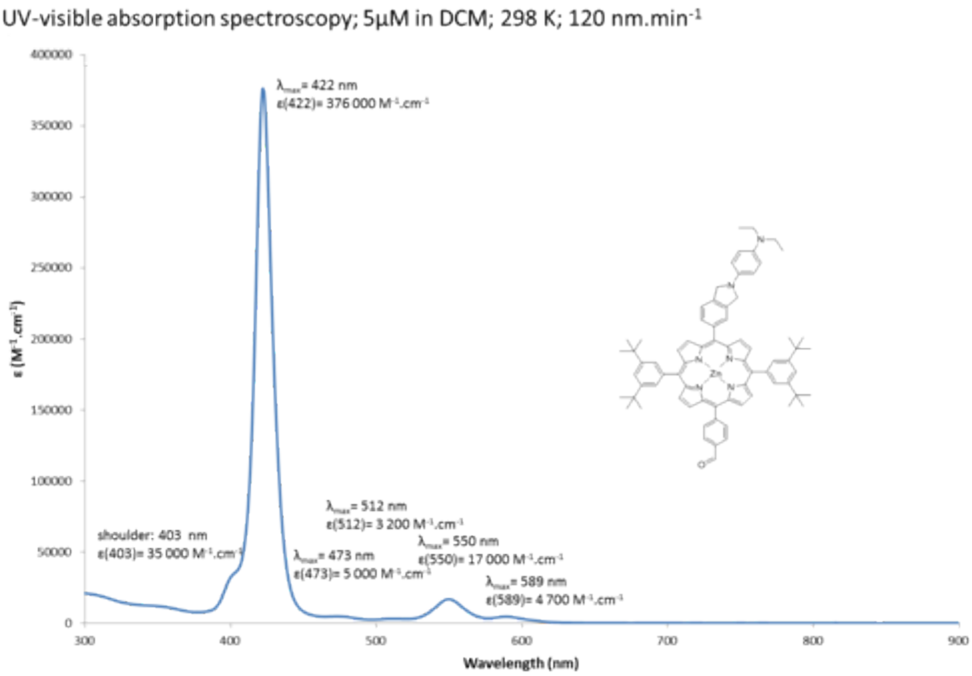

o. [5,15-Bis-(3,5-bis-*tert*-butylphenyl)-10-[4-(N-(3',4'-didodecoxybenzyl)-[60]fullero[c]tetrahydropyrrol-2-yl)phenyl)]-20-(4-(diethylamino)phenyl)-4H-isoindolin-2-yl)porphinato]zinc(II) (C_{60}-ZnP_{Ar}-TAPD)

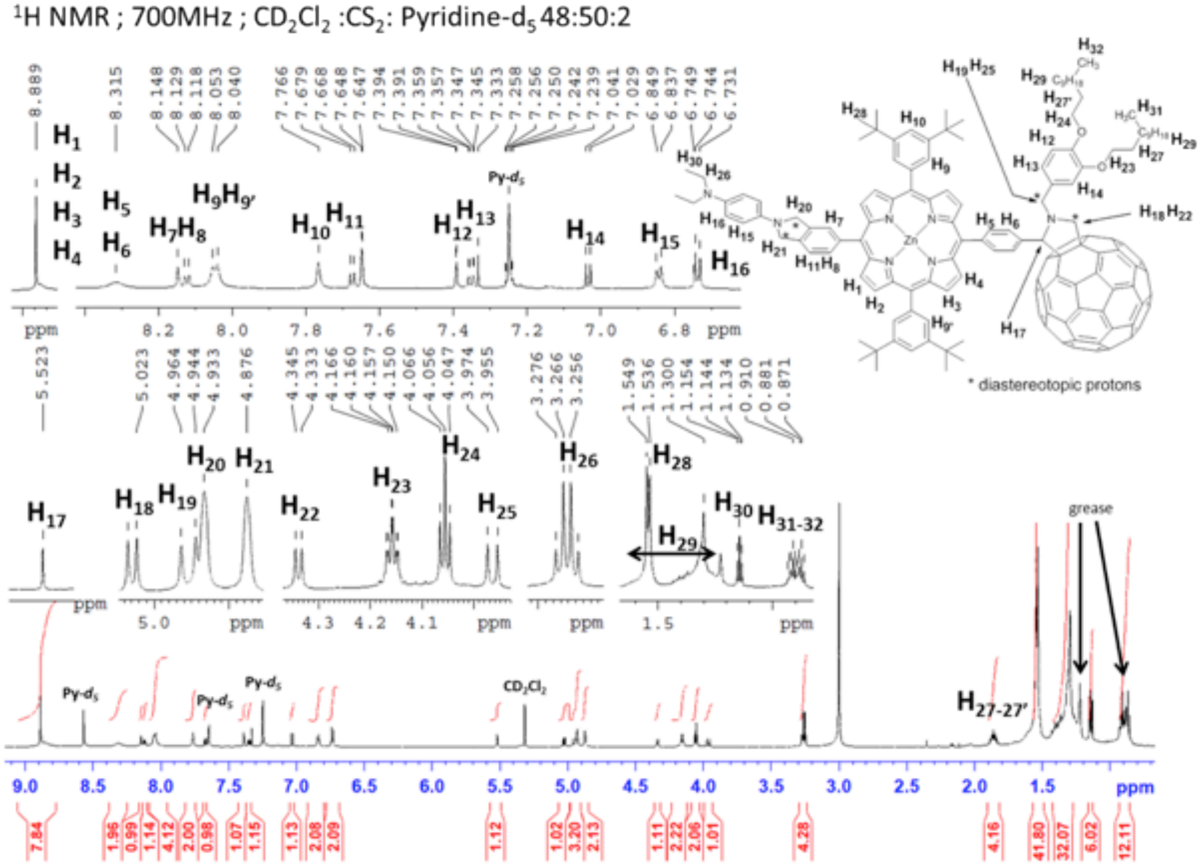

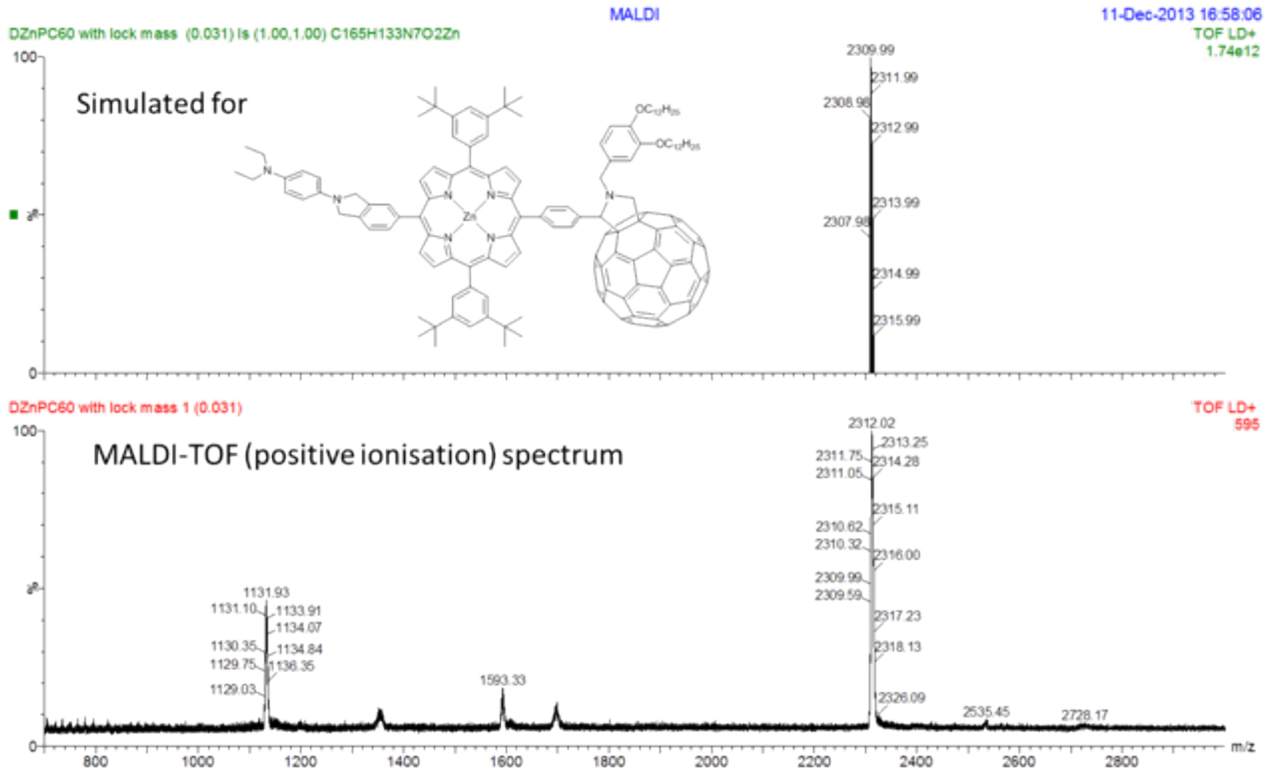

HMRS- MALDI TOF (Swansea National Mass-spectroscopy Facility):

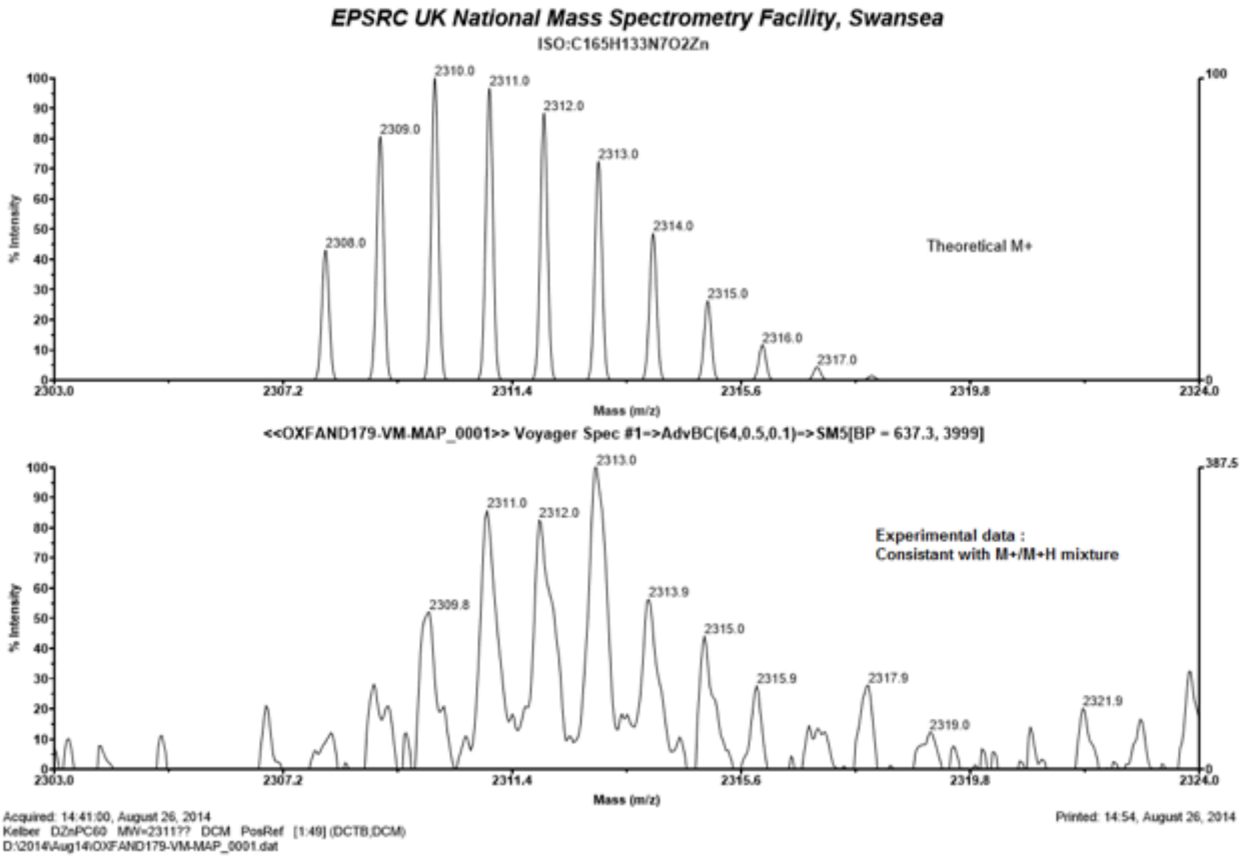

COSY (^1H-^1H) 400 MHz, $CDCl_3$+ 1% Py-d_5

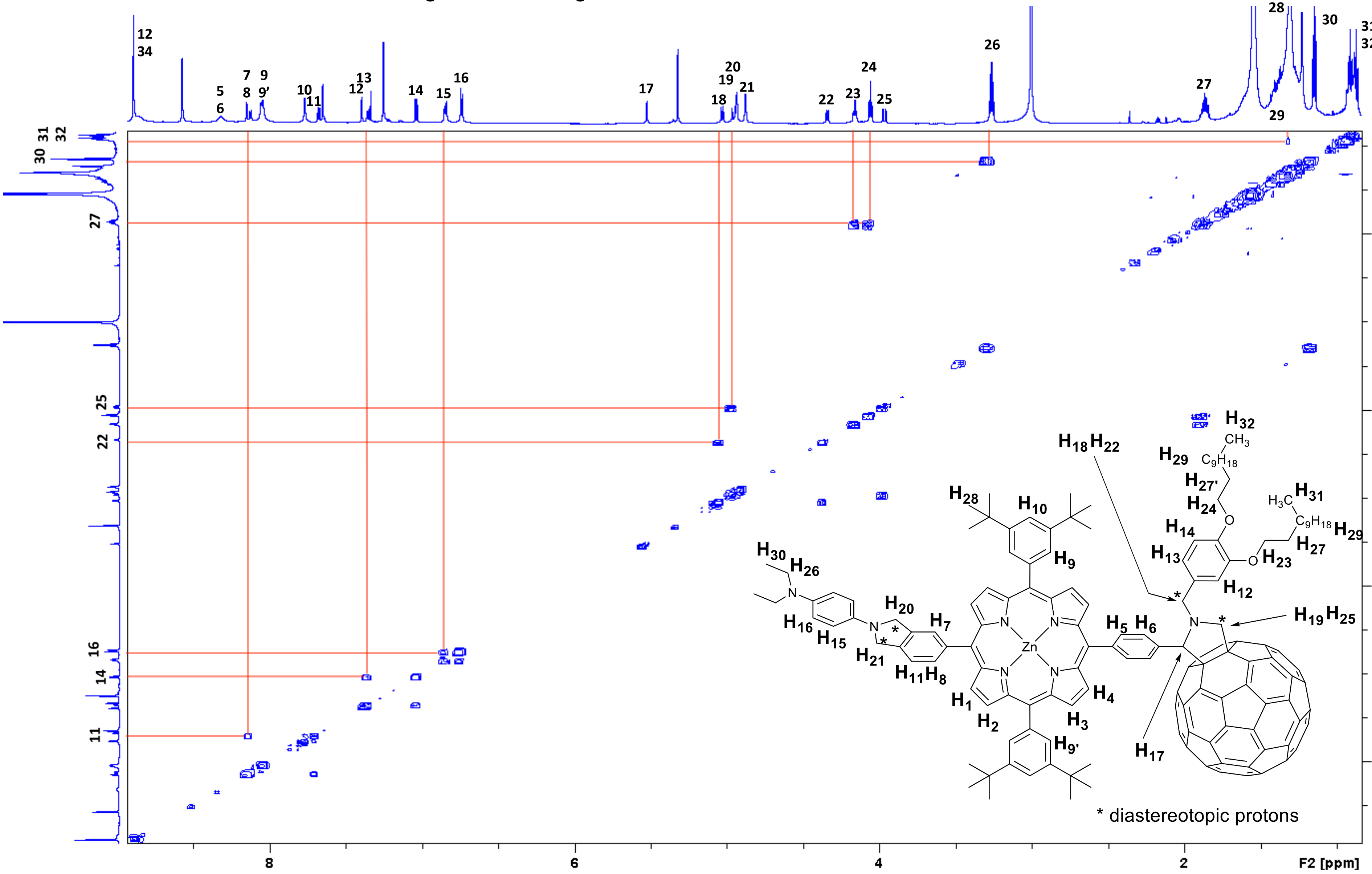

UV-visible absorption spectroscopy; 5 µM in toluene ; 298 K; 120 nm.min^{-1}

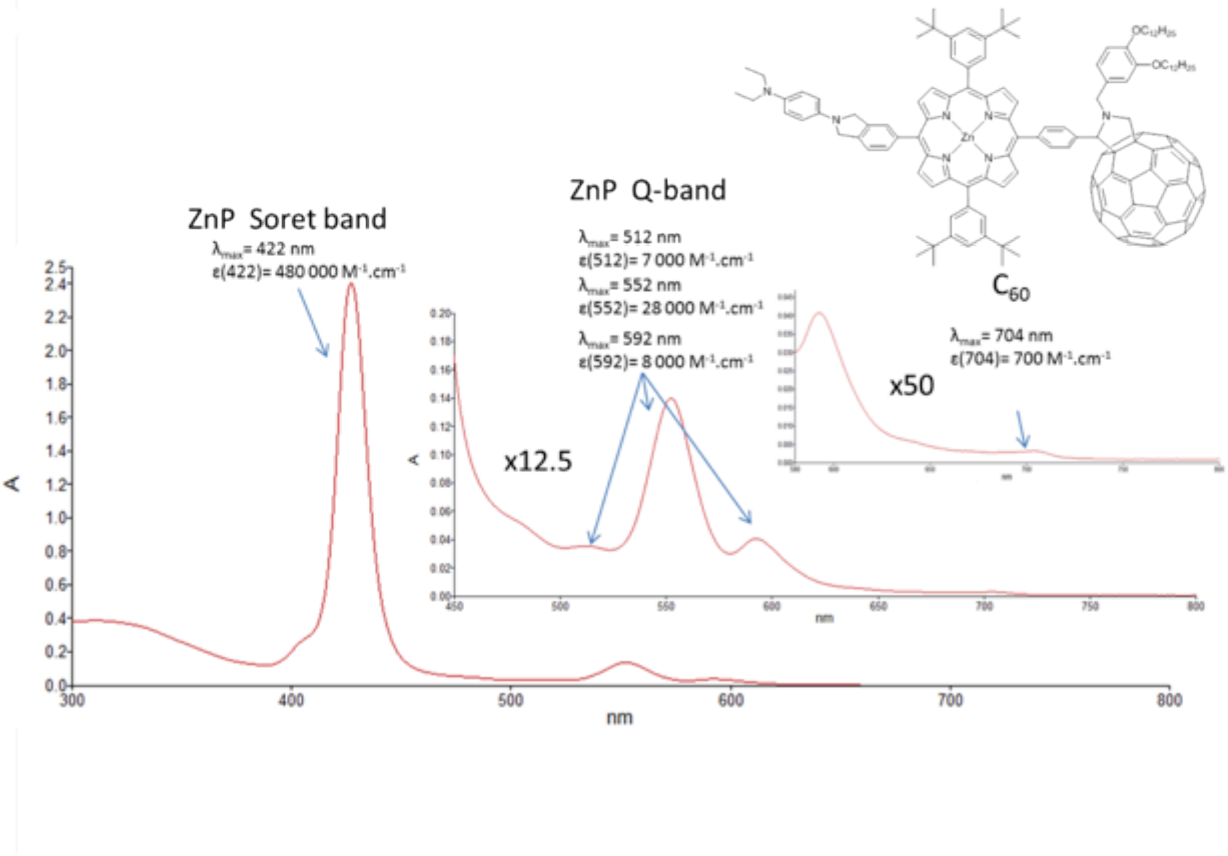

Squarewave voltametry of TAPD-ZnP$_{Ar}$-C$_{60}$ and related compounds in DCM with 0.1M Bu$_4$N PF$_6$ as electrolyte.

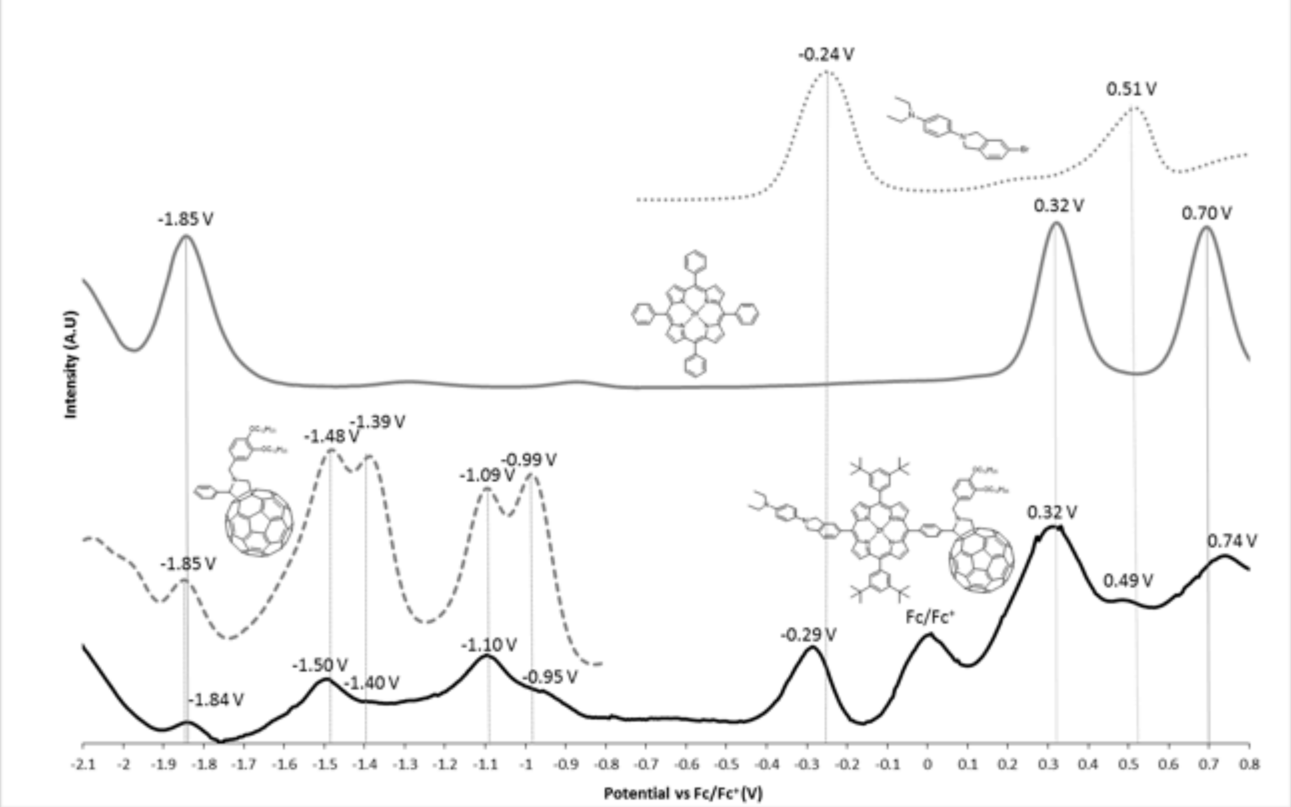

3) Synthesis of reference and known compounds

a. [5,15-Bis-(3,5-bis-*tert*-butylphenyl)-10,20-Bis-(4,4,5,5-tetramethyl-1,3,2-dioxaborolan-2-yl)porphinato]zinc(II) (13) + [5,15-Bis-(3,5-bis-*tert*-butylphenyl)-10-(4,4,5,5-tetramethyl-1,3,2-dioxaborolan-2-yl)porphinato]zinc(II) S1

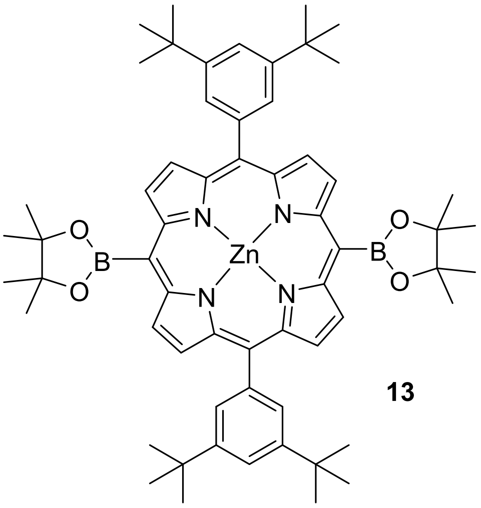

13

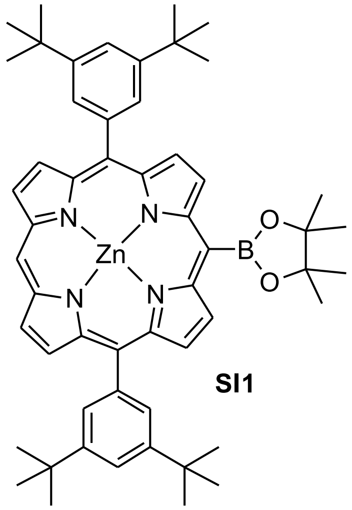

SI1

The procedure was adapted from a formerly reported method[S3]

A dry two-neck flask equipped with a condenser was charged with [5,15-bis-(3,5-bis-*tert*-butylphenyl)-10,20-dibromoporphinato]zinc(II)[S2] (400 mg; 440 µmol; 1 eq.) and *trans*-dichloro-di(triphenylphosphine)palladium(II) (62 mg; 88 µmol; 0.2 eq.). The mixture was purged 3 times with argon and dry toluene (40 mL), dry THF (40 mL) and distilled triethylamine (9 mL; 150 eq.) were added. The solution was purged 5 more times with argon and pinacolborane (2.30 mL; 15.8 mmol; 36 eq.) was added. The reaction mixture was stirred at 68 °C for one day. The excess of pinacolborane was neutralized by adding carefully water (20 mL, dropwise via syringe) at 0 °C. The organic phase was collected, washed three times with water (3 x 20 mL) and brine (20 mL), dried over $MgSO_4$ and filtered. The solvents were removed under reduced pressure and separation of **13** and **S1** was achieved by chromatography (SiO_2). The first fraction was collected while using dichloromethane 2:1 petroleum ether 40–60°C (**S1**; red powder; 76 mg; 21%) as eluent and the second fraction was collected while using dichloromethane/methanol 98/2 as eluent (**13**; pink powder; 326 mg; 74%).

^{1}H NMR (400 MHz, $CDCl_3$ + 1 % Pyridine-d_5): δ = 9.84 (d, 4.6Hz, 4H), 9.03 (d, 4.6Hz, 4H), 8.03 (d, 1.8 Hz, 4H), 7.77 (t, 1.8 Hz, 2H), 1.83 (s, 12H), 1.53 (s, 36H) ppm.

^{13}C NMR (100 MHz, $CDCl_3$ + 1 % Pyridine-d_5): δ = 152.9, 150.0, 147.9, 142.5, 132.3, 132.0, 129.7, 125.2, 121.4, 120.2, 84.8, 34.8, 31.6, 25.1 ppm.

^1H NMR ; 400MHz ; $CDCl_3$+ 1% Pyridine-d_5

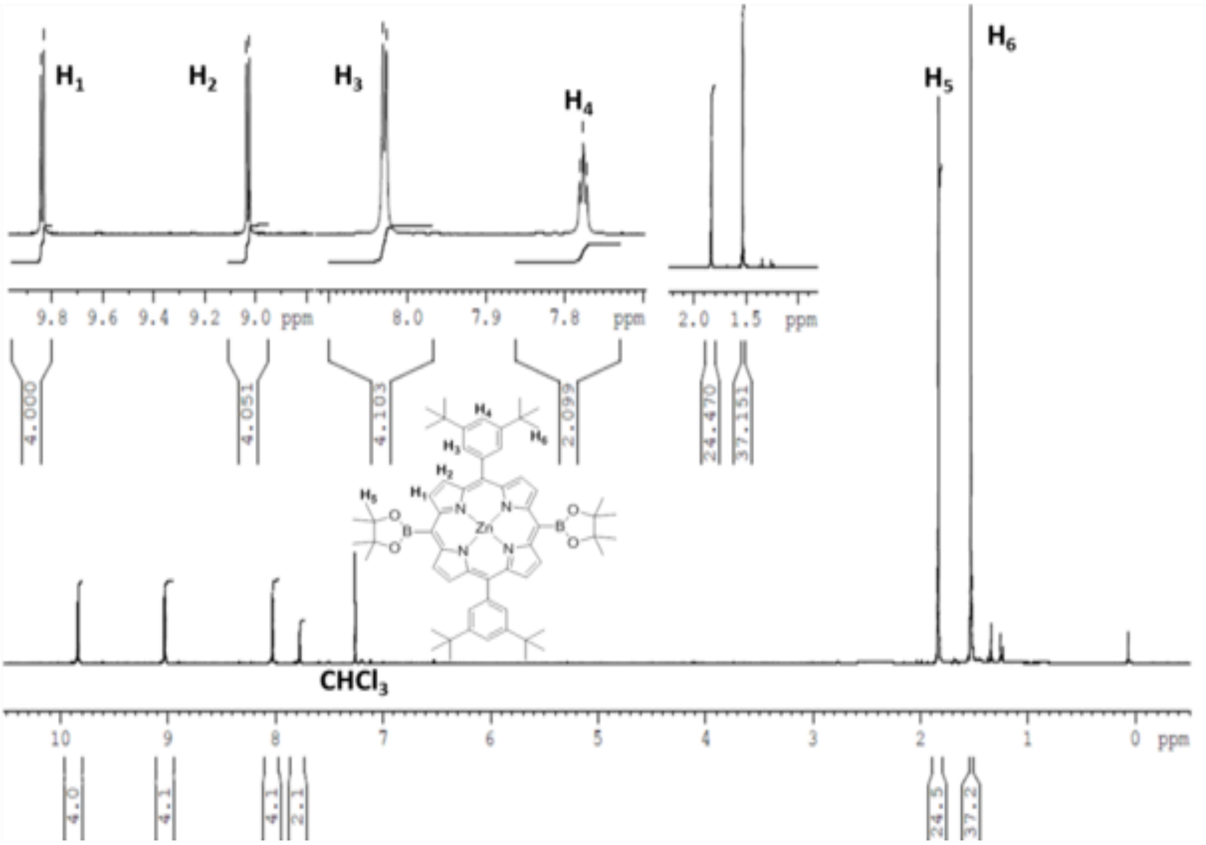

^1H NMR ; 400MHz ; $CDCl_3$+ 1% pyridine-d_5

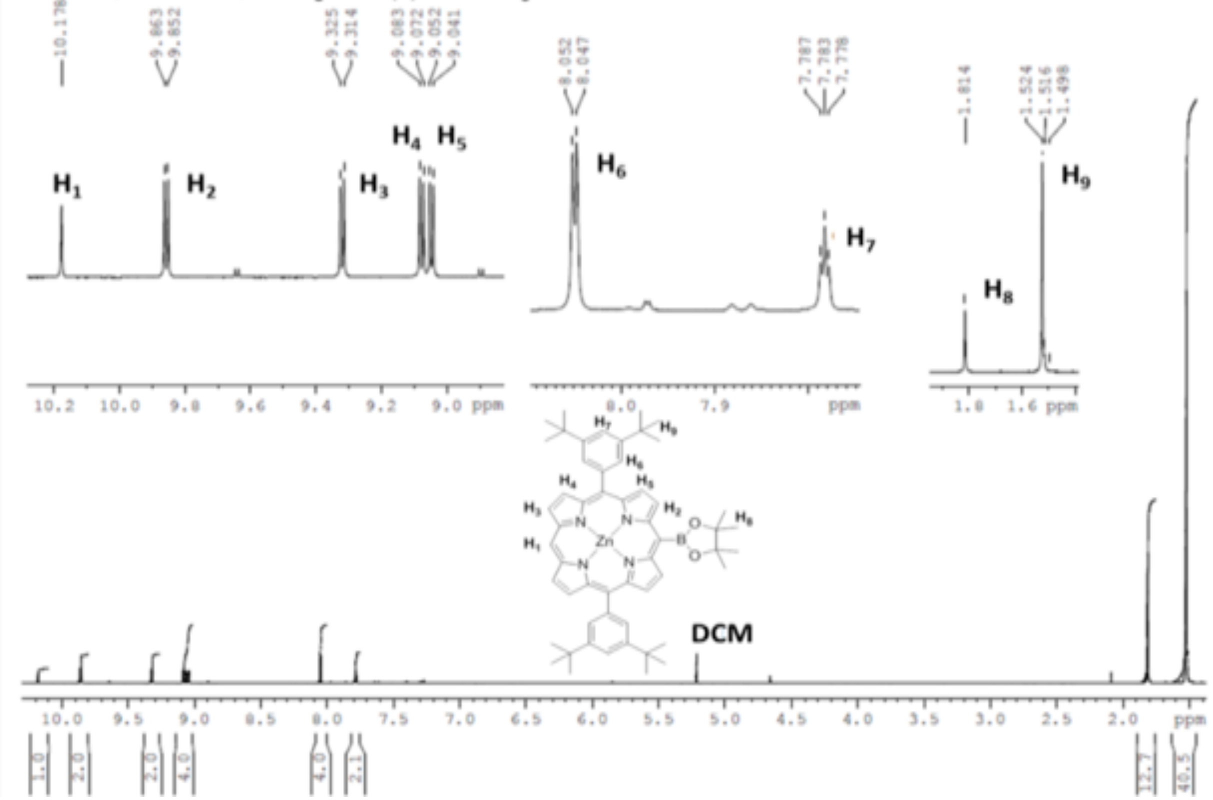

b. 2-((3,4-Bis(dodecyloxy)benzyl)amino)acetic acid (S3)

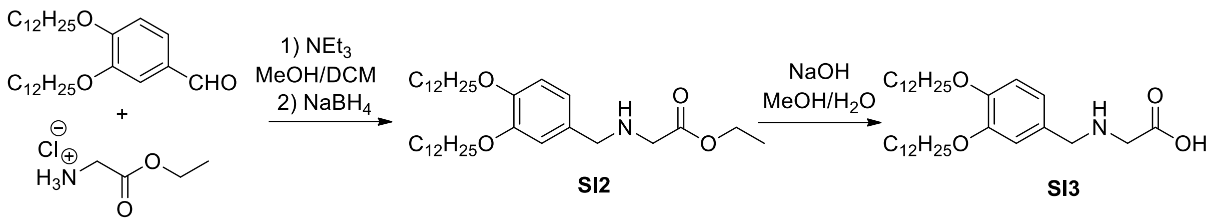

2-((3,4-Bis(dodecyloxy)benzyl)amino)acetic acid **S3** was synthesized in two steps: reductive amination of 3,4-didocylbenzaldehyde with glycine ethyl ester and sodium borohydride, followed by saponification of the ester with aqueous sodium hydroxide.

3,4-Didodecyloxybenzaldehyde (1.4 g; 10 mmol; 1.25 eq.) was dissolved in a methanol/dichloromethane (10/1 mixture; 20 mL). Triethylamine (1.4 mL; 10 mmol; 1.25 eq.) and glycine ethyl ester hydrochloride (4.2 g; 8 mmol; 1 eq.) were added. The mixture was stirred for 1 h at room temperature and sodium borohydride (0.72 g; 20 mmol; 2.5 eq.) was then added in portions at 0 °C. The solution was subsequently stirred overnight at room temperature. Water (10 mL) was added and the amino-ester **SI2** was extracted with ethyl acetate (2 x 15 mL), washed with water (2 x 10 mL) and brine (1 x 10 mL), dried over $MgSO_4$ and filtrated. The solvent was evaporated, and **S2** was further purified by chromatography (SiO_2; *Eluent 1* = petroleum ether 40–60 °C /ethyl acetate 4/1 to remove the remaining starting material; *Eluent 2* = petroleum ether 40–60 °C /ethyl acetate 1/1 to get the desired product). This yielded **S2** as a pale yellow oil, which was then added mixture of aqueous concentrate sodium hydroxide (10 mL; 277 mmol) and methanol (5 mL). The suspension was then vigorously stirred 2 days at 70 °C and the solution was neutralized at 0 °C with concentrated aqueous hydrochloric acid (23.1 mL; 277 mmol). The precipitate was then filtered-off, washed several time with water until the pH of the mother-liquid reaches 7, and dried under vacuum over P_2O_5 to yield **S3** as a white powder (3.4 g; 6.3 mmol; 79%).

^{1}H NMR ; 400MHz ; $CDCl_3$+ TFA

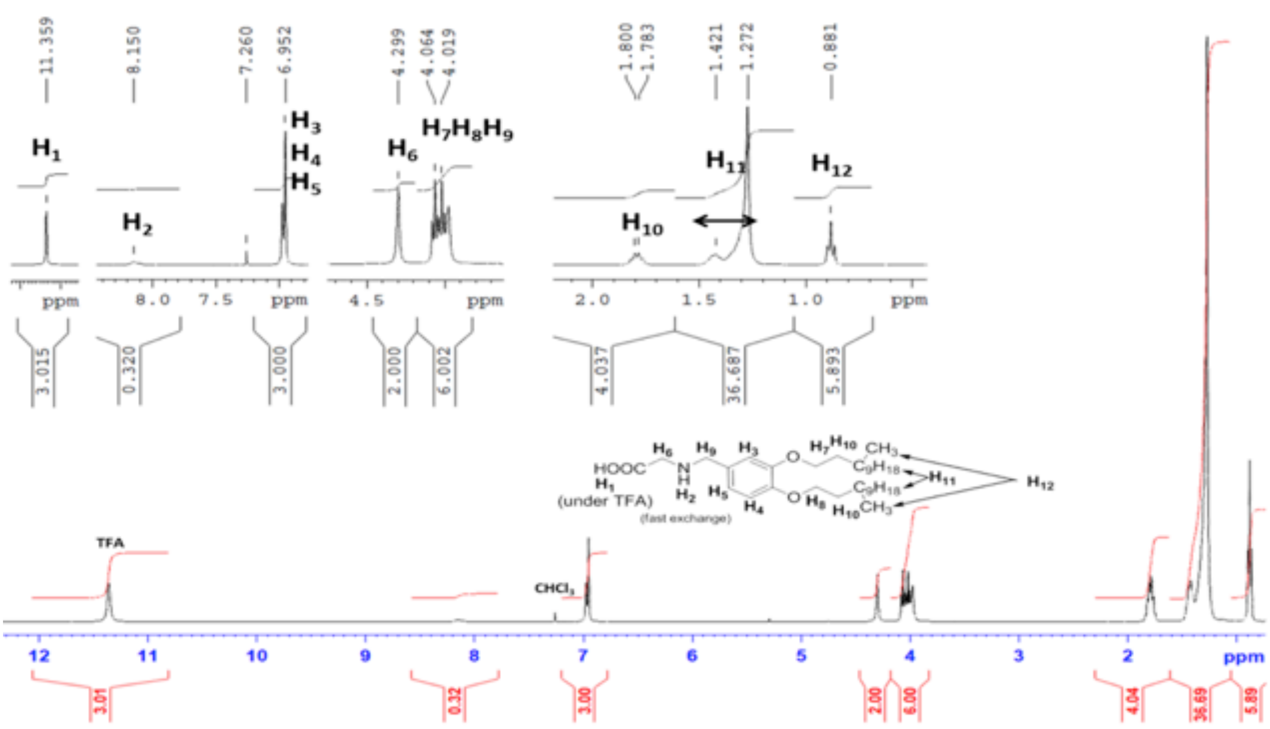

^{13}C NMR ; 100MHz ; $CDCl_3$+ 1% Pyridine-d_5

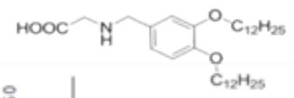

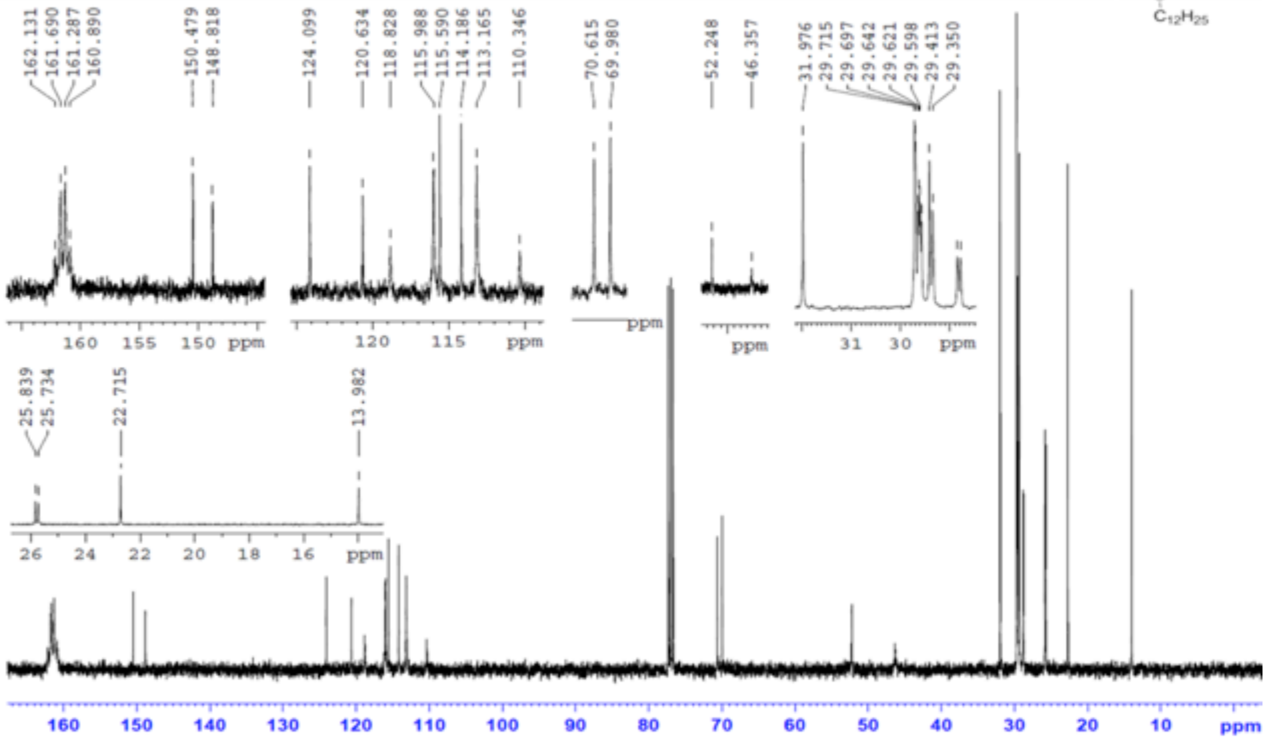

Infra-red vibrational spectroscopy (neat)

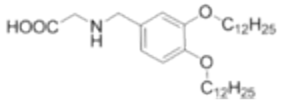

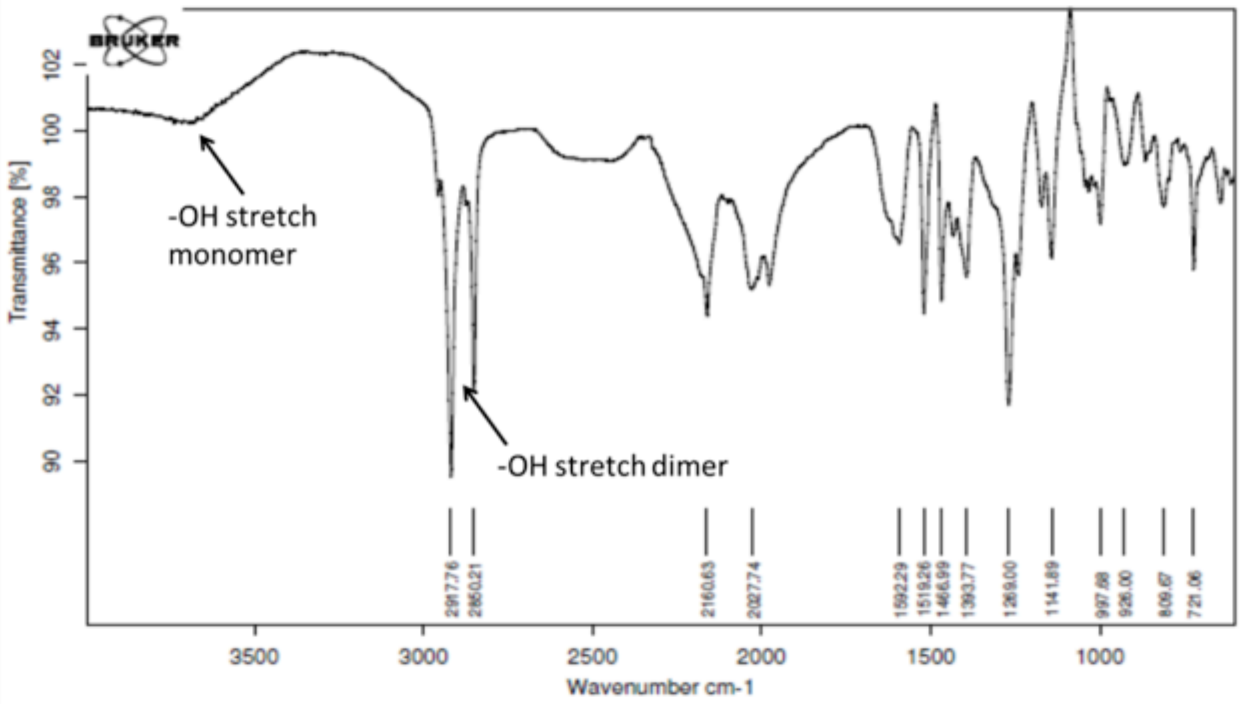

c. [5,15-Bis-(3,5-bis-*tert*-butylphenyl)-10(4-(diethylamino)phenyl)-*4H*-isoindolin-2-yl)porphinato]zinc(II) (SI4) and [5,15-Bis-(3,5-bis-*tert*-butylphenyl)-10-(5,12-[1,2]benzenotetracen-2-yl-6,11(5H,12H)-dione)porphinato]zinc(II) (S5)

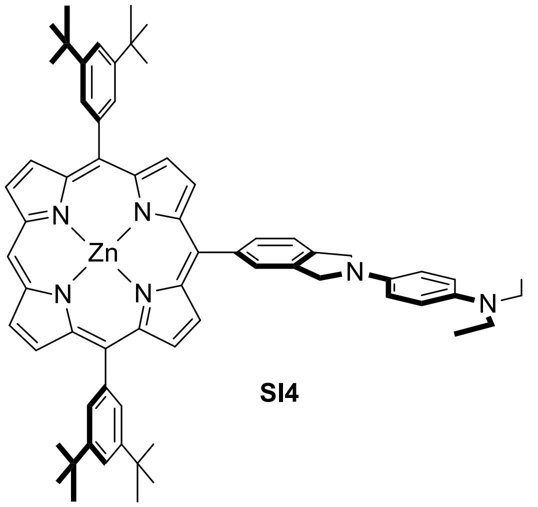

SI4

A dry two-neck flask equipped with a condenser was charged with **S1** (100 mg; 114 µmol; 1 eq.), **7** (47 mg; 136 µmol; 1.2 eq.), freshly recrystallized tetrakistriphenylphosphine palladium(0) (13 mg; 11.4 µmol; 0.1 eq.) and cesium carbonate (111 mg; 342 µmol; 3 eq.). The mixture was purged 3 times with argon and a mixture of toluene (10 mL) and pyridine (150 µL) was added. The solution was purged 5 more times with argon and the reaction mixture was refluxed for 7 h. The solvent was evaporated and the product was purified by chromatography (SiO_2) using dichloromethane 98 : 2 methanol as eluent and further purified by size-exclusion column (eluent chloroform +1% pyridine) to yield after drying under vacuum **S4** as purple powder (95 mg; 93 µmol; 82%).

^1H NMR (400 MHz, $CDCl_3$ + 1 % Pyridine-d_5): δ = 10.16 (s, 1H), 9.36 (d, 4.5 Hz, 2H), 9.11 (d, 4.5 Hz, 2H), 9.02 (d, 4.5 Hz, 2H), 8.96 (d, 4.5 Hz, 2H), 8.17 (m, 2H), 8.12 (d, 1.8 Hz, 4H), 7.82 (t, 1.8 Hz, 2H), 7.65 (d, 8.3 Hz, 1H), 6.92 (m, 2H), 8.77 (m, 2H), 4.93 (s, 2H), 4.89 (s, 2H), 1.58 (s, 36H) ppm.

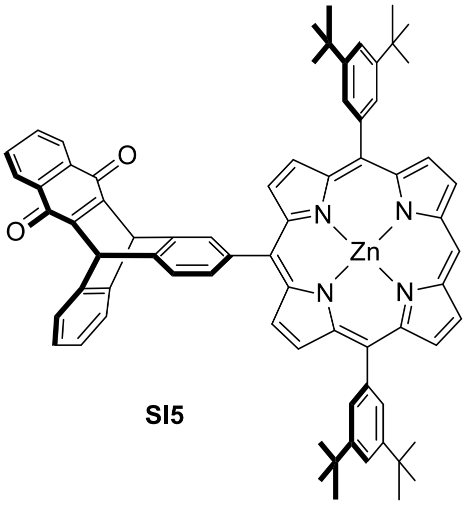

SI5

A dry two-neck flask equipped with a condenser was charged with **S1** (10.0 mg; 11.4 µmol; 1 eq.), 7-bromo-triptycenenaphtoquinone (intermediate in the synthesis of **10**, mixture with triptycenenaphthoquinone, 6.1 mg; 13 µmol; 1.2 eq.), freshly recrystallized tetrakistriphenylphosphine palladium(0) (1.2 mg; 1.1 µmol; 0.1

eq.) and cesium carbonate (10.8 mg; 33.1 µmol; 3.0 eq.). The mixture was purged 3 times with argon and a mixture of toluene (2 mL) and pyridine (30 µL) was added. The solution was purged 5 more times with argon and the reaction mixture was refluxed for 2 days. The solvent was evaporated and the product was purified by chromatography (SiO_2) using dichloromethane 3 : 1 petrol ether 40–60°C as eluent to yield after drying under vacuum **SI5** as purple powder (7.1 mg; 6.6 µmol; 60%).

^1H NMR (400 MHz, $CDCl_3$ + 1 % Pyridine-d_5): δ = 10.11 (s, 1H), 9.31 (d, 4.6 Hz, 1H), 9.30 (d, 4.6 Hz, 1H), 9.04 (d, 4.6 Hz, 1H), 8.86 (d, 4.6 Hz, 1H), 8.85 (d, 4.6 Hz, 1H), 8.76 (d, 4.6 Hz, 1H), 8.73 (d, 4.6 Hz, 1H), 8.28 (d, 1.6 Hz, 1H), 8.18 (m, 1H), 8.11 (m, 1H), 8.04 (m, 2H), 8.02 (m, 2H), 7.88 (dd, 7.2 Hz, 1.6 Hz, 1H), 7.77 (d, 7.2 Hz, 1H), 7.73 (m, 4H), 7.66 (m, 1H), 7.55 (dd, 7.2Hz, 1.2 Hz, 1H), 7.16 (m, 2H), 6.31 (s, 1H), 6.15 (s, 1H), 1.52 (s, 9H) , 1.51 (s, 9H) , 1.50 (s, 9H) , 1.49 (s, 9H) ppm.

4) Assignment of the TAPD-ZnP$_{Ar}$-TNQ triad ^{1}H NMR spectrum using reference compounds

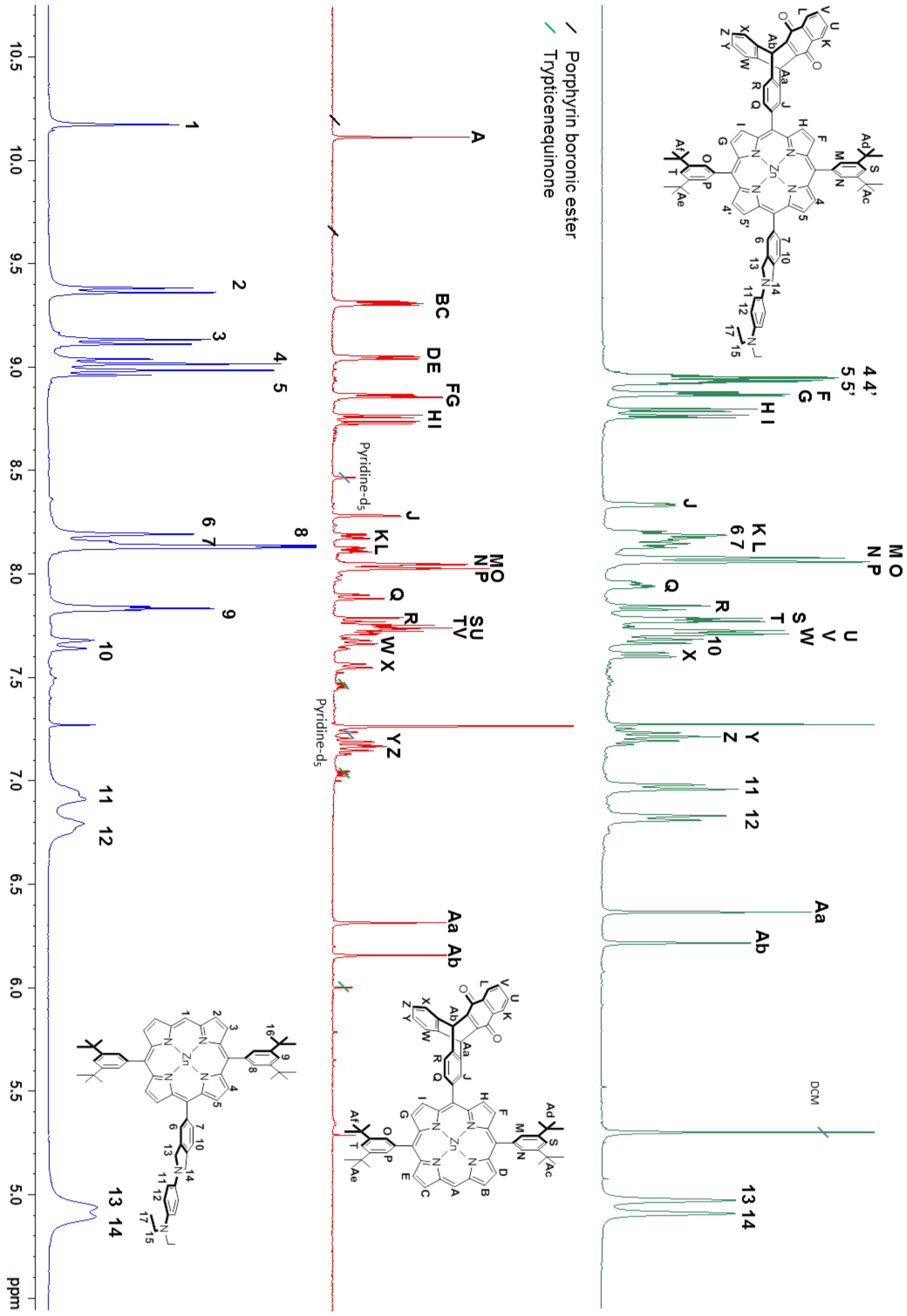

5) Frontier orbital energies

B3LYP/6-31G*

	TNQ-ZnP-TAPD	TNQ-ZnP$_{Ar}$-TAPD	C_{60}-ZnP$_{Ar}$-TAPD
HOMO–2	–4.90	–5.08	–5.12
HOMO–1	–4.79	–4.92	–4.95
HOMO	–4.35	–4.25	–4.27
LUMO	–3.02	–3.07	–3.10; –2.99; –2.75*
LUMO+1	–1.82	–2.08	–2.10
LUMO+2	–1.80	–2.05	–2.09

* The LUMO of C_{60} is triply degenerate, but the saturation at the pyrrolidine linking breaks the symmetry.

M062X /6-31G*

	TNQ-ZnP-TAPD	TNQ-ZnP$_{Ar}$-TAPD	C_{60}-ZnP$_{Ar}$-TAPD
HOMO–2	–5.91	–6.04	–6.08
HOMO–1	–5.71	–5.86	–5.89
HOMO	–5.66	–5.62	–5.85
LUMO	–2.12	–2.09	–2.61 / –2.47 / –2.21
LUMO+1	–1.34	–1.59	–1.64
LUMO+2	–1.33	–1.57	–1.62

CAM–B3LYP /6-31G*

	TNQ-ZnP-TAPD	TNQ-ZnP$_{Ar}$-TAPD	C_{60}-ZnP$_{Ar}$-TAPD
HOMO–2	–5.90	–5.98	–6.02
HOMO–1	–5.66	–5.89	–5.93
HOMO	–5.61	–5.59	–5.78
LUMO	–1.84	–1.81	–2.14 / –1.99 / –1.73
LUMO+1	–0.96	–1.20	–1.24
LUMO+2	–0.96	–1.18	–1.22

ω-B97xD/6-31G*

	TNQ-ZnP-TAPD	TNQ-ZnP$_{Ar}$-TAPD	C_{60}-ZnP$_{Ar}$-TAPD
HOMO–2	–4.90	–6.51	–6.55
HOMO–1	–4.79	–6.38	–6.42
HOMO	–4.35	–6.17	–6.41
LUMO	–3.02	–1.27	–1.71 / –1.55 / –1.29
LUMO+1	–1.82	–0.74	–0.78
LUMO+2	–1.80	–0.71	–0.75

HF/6-31G*

	TNQ-ZnP-TAPD	TNQ-ZnP$_{Ar}$-TAPD	C_{60}-ZnP$_{Ar}$-TAPD
HOMO–2	–6.43	–6.48	–6.51
HOMO–1	–5.64	–5.93	–5.95
HOMO	–6.69	–7.32	–7.29
LUMO	0.75	0.83	–0.18 / –0.05 / 0.33
LUMO+1	1.04	0.76	0.74
LUMO+2	1.10	0.77	0.80

6) Frontier orbitals distribution

a. Triad TNQ-ZnP-TAPD

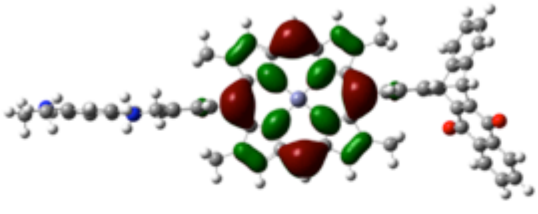

HOMO-2 ; -5.99 eV

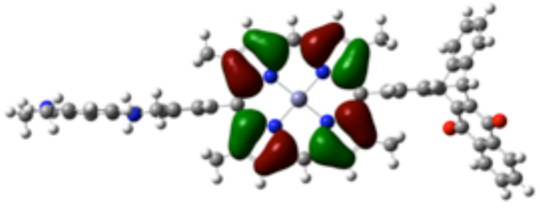

HOMO-1 ; -5.82 eV

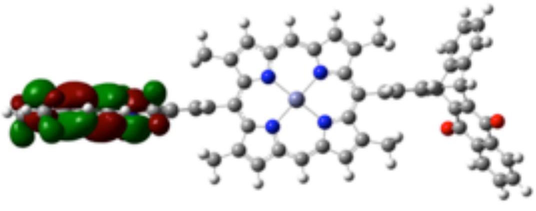

HOMO ; -5.63 eV

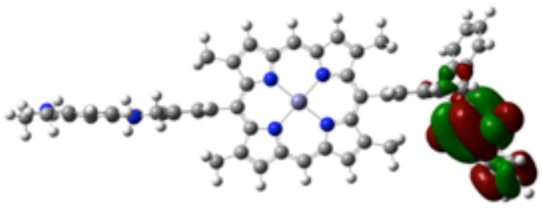

LUMO ; -1.85 eV

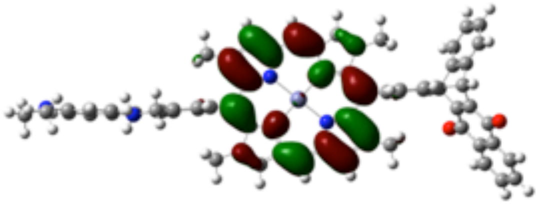

LUMO+1 ; -1.06eV

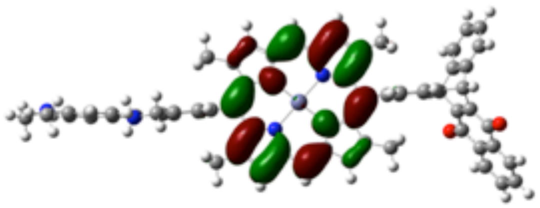

LUMO+2 ; -1.17 eV

b. Triad TNQ-ZnP$_{Ar}$-TAPD

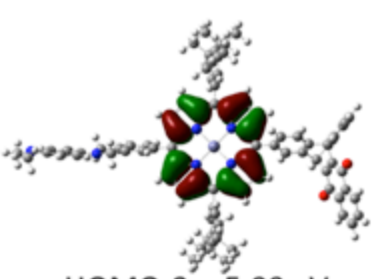

HOMO-2 ; -5.99 eV

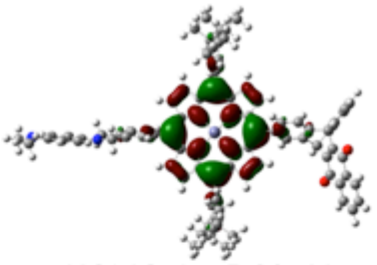

HOMO-1 ; -5.88 eV

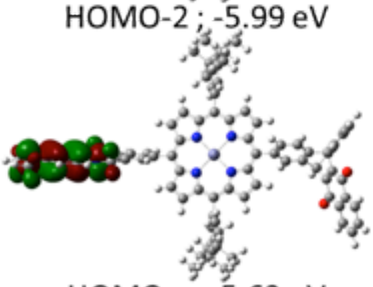

HOMO ; – 5.63 eV

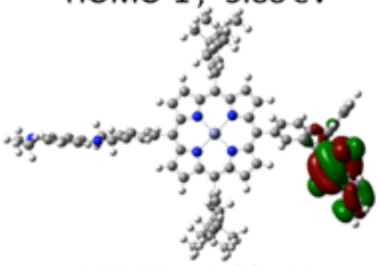

LUMO ; -1.80 eV

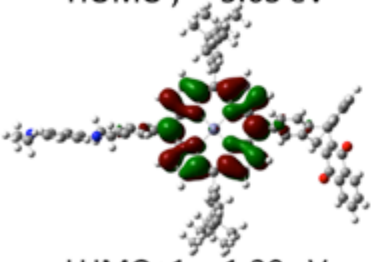

LUMO+1 ; -1.20 eV

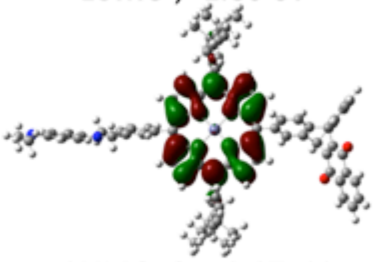

LUMO+2 ; -1.17 eV

c. Triad C_{60}-ZnP_{Ar}-TAPD

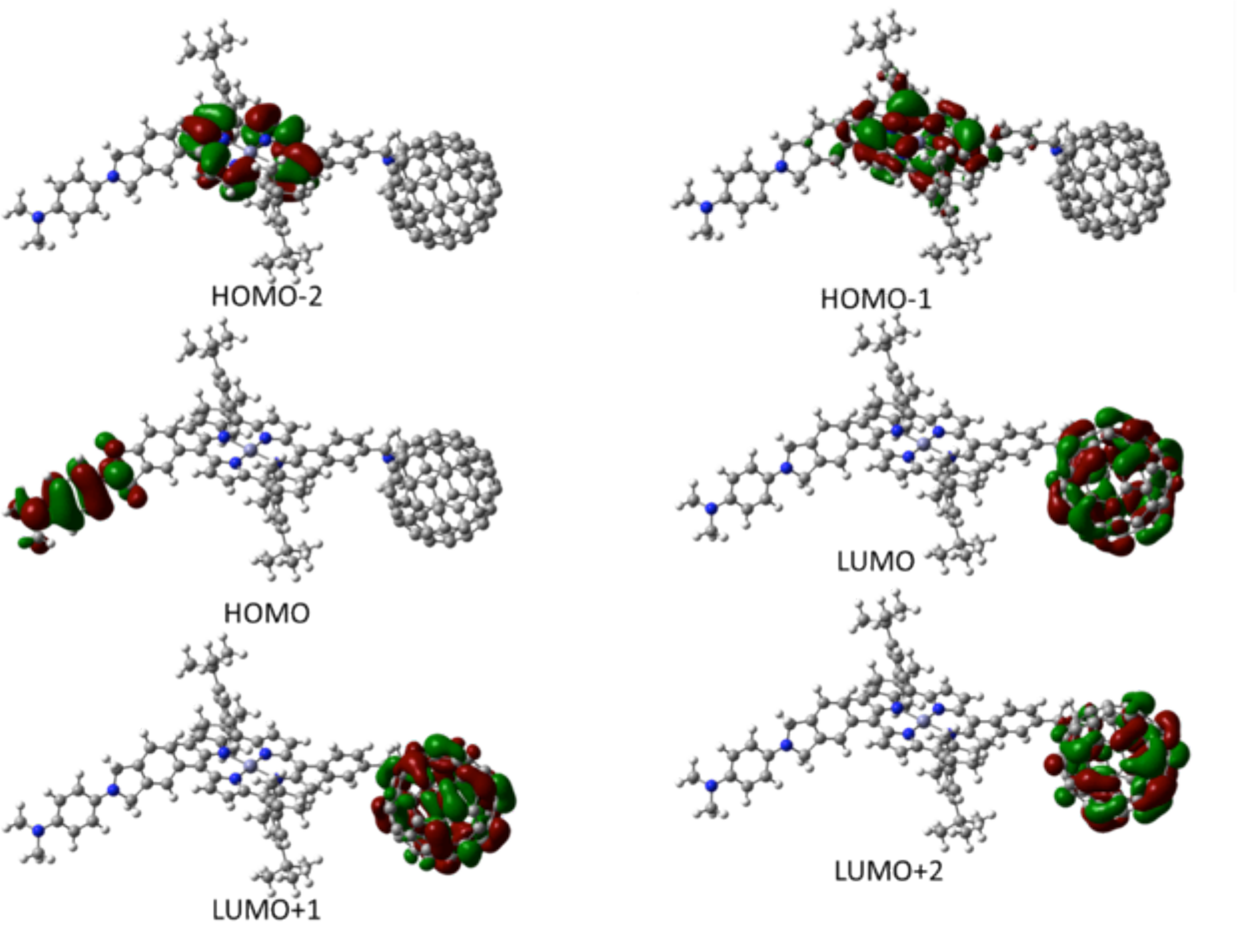

7) Ionization energy and electron affinity of moieties

Ionization potential (IP) and electron affinity (EA) (eV)

6-31G*, PCM in butyronitrile	CAM-B3LYP		M062X		B3LYP	
	IP	EA	IP	EA	IP	EA
TADP	4.24		4.42		4.15	
ZnP_{Ar}	4.98	–2.48	5.16	–2.43	4.95	–2.75
ZnP	4.72	–2.31	5.03	–2.22	4.81	–2.47
TNQ		–3.37		–3.48		–3.40
C_{60}		–3.03		–3.31		–3.20

6-31G*, PCM in acetonitrile	CAM-B3LYP		M062X		B3LYP	
	IP	EA	IP	EA	IP	EA
TADP	4.22		4.40		4.13	
ZnP_{Ar}	4.96		5.21		4.94	–2.67
ZnP	4.71		5.01		4.79	–2.45
TNQ		–3.40		–3.51		–3.42
C_{60}		–3.05		–3.33		–3.22

6-31G*, PCM in tetrahydrofurane	CAM-B3LYP		M062X		B3LYP	
	IP	EA	IP	EA	IP	EA
TADP	4.37		4.55		4.28	
ZnP_{Ar}	5.09		5.33		5.06	
TNQ		–3.17		–3.28		–3.20
C_{60}		–2.91		–3.19		–3.08

6-31G*, Gas Phase	CAM-B3LYP		M062X		B3LYP	
	IP	EA	IP	EA	IP	EA
TADP	5.68		5.83		5.58	
ZnP_{Ar}	6.15		6.32		6.03	
ZnP			6.47			
TNQ		–1.60		–1.68		–1.65
C_{60}		–1.81		–2.09		–1.99

6-311G**, PCM in butyronitrile	CAM-B3LYP		M062X		B3LYP	
	IP	EA	IP	EA	IP	EA
TADP	4.49		4.63		4.41	
TNQ		–3.84		–3.88		–3.86

6-311G**, PCM in acetonitrile	CAM-B3LYP		M062X		B3LYP	
	IP	EA	IP	EA	IP	EA
TADP	4.48		4.61		4.39	
TNQ		–3.87		–3.91		–3.89

8) Inner-sphere reorganization energies

We report inner-sphere reorganization energies at several levels of theory and solvent combinations for the moieties. These were computed as $\lambda_S = E^R{}_S - E^P{}_S$, where R and P represent reaction and product, and S represents the potential energy surface: either the charged (–1*e* or +1*e*, for anion and cation respectively), or the neutral surfaces (*N*).

Reorganization Energy (eV)									
		CAM-B3LYP			B3LYP			M062X	
6-31G* PCM in Gas Phase	–1 e	N	+1 e	–1 e	N	+1 e	–1 e	N	+1 e
TAPD	0.35	0.23		0.27	0.18		0.42	0.24	
TNQ		0.26	0.25		0.21	0.20		0.26	0.26
		CAM-B3LYP			B3LYP			M062X	
6-31G* PCM in THF	–1 e	N	+1 e	–1 e	N	+1 e	–1 e	N	+1 e
TADP	0.37	0.22		0.29	0.17		0.44	0.24	
ZnP_{Ar}	0.05	0.05		0.04	0.04		0.07	0.13	
TNQ		0.26			0.21	0.21		0.27	0.26
C_{60}					0.07	0.07		0.09	0.09
		CAM-B3LYP			B3LYP			M062X	
6-31G* PCM in butyronitrile	–1 e	N	+1 e	–1 e	N	+1 e	–1 e	N	+1 e
ZnP_{Ar}	0.06	0.05							
C_{60}		0.09	0.09		0.07	0.07		0.09	0.09
		CAM-B3LYP			B3LYP			M062X	
6-31G*, PCM in acetonitrile	–1 e	N	+1 e	–1 e	N	+1 e	–1 e	N	+1 e
TADP	0.37	0.22		0.29	0.17		0.44	0.23	
ZnP_{Ar}	0.05	0.05		0.04	0.05		0.07	0.13	
ZnP	0.06	0.06		0.19	0.05		0.08	0.13	
TNQ		0.26	0.25		0.21	0.21		0.27	0.26
C_{60}					0.07	0.07		0.09	0.09

		CAM-B3LYP			B3LYP			M062X	
6-311G** PCM in THF	–1 e	N	+1 e	–1 e	N	+1 e	–1 e	N	+1 e
TADP	0.39	0.23		0.31	0.18		0.46	0.24	
TNQ		0.28	0.28		0.23	0.23		0.29	0.28
C_{60}						0.07			
		CAM-B3LYP			B3LYP			M062X	
6-311G** PCM in acetonitrile	–1 e	N	+1 e	–1 e	N	+1 e	–1 e	N	+1 e
TADP	0.40	0.27		0.31	0.18		0.47	0.24	
TNQ		0.28	0.28		0.23	0.23		0.29	0.28
C_{60}					0.07	0.07		0.08	0.09

9) Predicted Recombination Rates

We report here the recombination rates from the long-lived radical pair state to the ground state, and the parameters used for that prediction:

- *ΔG* corresponds to the electronic energy difference between the ground state and the charge transfer state in solution (butyronitrile) at their respective equilibrium geometries. It corresponds to the energy difference between the bottoms of the parabola in the Marcus approximation.
- λ_{out} corresponds to the outer-sphere reorganization energy in upon electron transfer in solution. In frozen solvent, it is added to Δ*G*, to obtain the effective *ΔG* in frozen solvent, to capture the fact that only the electronic degrees of freedom of the solvent can respond to the charge transfer.
- λ_{in}^{vib} corresponds to the inner-sphere reorganization calculated as ($\lambda_j = \sum_i S_{ij}\omega_{ij}$). We report different values depending of the energy surface on which the vibrational modes were calculated (the neutral or the charge-transfer species) and also depending on whether whole triads were used, or only the displacements and vibrational motions of isolated donor and acceptor units.
- Exclusively for reference, since it does not play any role in the calculation of the lifetimes, we report λ_{in}^{diff}***.*** This magnitude corresponds to the energy differences between potential energy surfaces at either the reactant or product equilibrium geometry. They are reported above and included here for comparison with the values calculated from vibronic displacements.

Predicted recombination lifetimes for the TNQ-ZnP$_{Ar}$-TAPD triad

			**Δ*E* (eV)**	**λ_{out} (eV)**	**λ_{in}^{vib} (eV)**	**λ_{in}^{diff} (eV)**	**Lifetime (ms)**
**CAMB3LYP**	**Neutral**	**Parts**	0.79	1.38	0.52	0.48	0.4
		**Whole**	0.79	1.38	0.52	0.48	0.4
	**CT**	**Parts**	0.79	1.38	0.62	0.62	0.1
		**Whole**	0.79	1.38	0.57		0.3
**M062X**	**Neutral**	**Parts**	0.85	1.32	0.59	0.50	0.2
		**Whole**	0.85	1.32	0.69	0.49	0.05
	**CT**	**Parts**	0.85	1.32	0.71	0.70	0.04
		**Whole**	0.85	1.32	0.59		0.6
**B3LYP**	**Neutral**	**Parts**	0.72	1.27	0.43	0.38	1.3
		**Whole**	0.72	1.27	0.44	0.38	1.3
	**CT**	**Parts**	0.72	1.27	0.51	0.50	0.4
		**Whole**	0.72	1.27	0.48	0.50	0.9

Predicted recombination lifetimes for the TNQ-ZnP$_{Ar}$-TAPD triad

			**Δ*E* (eV)**	**λ_{out} (eV)**	**λ_{in}^{vib} (eV)**	**λ_{in}^{diff} (eV)**	**Lifetime (ms)**
**CAMB3LYP**	**Neutral**	**Parts**	1.01	1.25	0.35	0.31	1475
		**Whole**	1.01	1.25	0.38	0.31	443
	**CT**	**Parts**	1.01	1.25	0.44	0.46	85
		**Whole**	1.01	1.25	0.38		875
**M062X**	**Neutral**	**Parts**	0.92	1.21	0.40	0.32	278
		**Whole**	0.92	1.21	0.43	0.31	114
	**CT**	**Parts**	0.92	1.21	0.53	0.53	5.5
		**Whole**	0.92	1.21	0.49		23.3
**B3LYP**	**Neutral**	**Parts**	0.75	1.21	0.27	0.24	2700
		**Whole**	0.75	1.21	0.28	0.24	1200
	**CT**	**Parts**	0.75	1.21	0.34	0.36	255
		**Whole**	0.75	1.21	0.31		741

10) Analysis of the Influence of Conformational Factors on Electronic Coupling

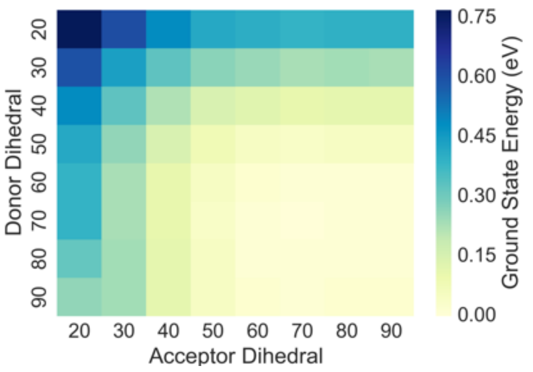

Figure S1. Ground state DFT/B3LYP/6-31G* energy of **TNQ-ZnP$_{Ar}$-TAPD** as a function of dihedral angle. The more co-planar conformations only occur at a high-energy cost. There is a single overall minimum described with dihedral angles at around 70 degrees.

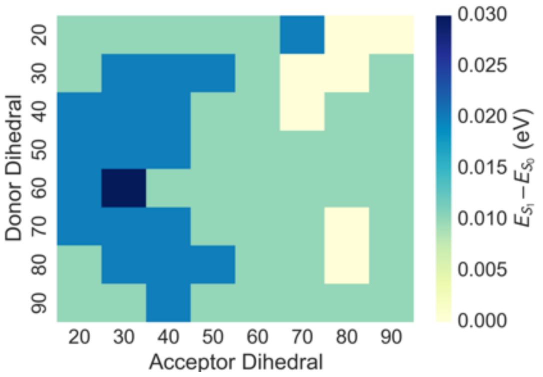

Figure S2. Excitation energy to the CSS$_2$ charge-transfer state TD-DFT/B3LYP/6-31G* energy of **TNQ-ZnP$_{Ar}$-TAPD** as a function of dihedral angle, in fluid solution. The energy of the excited state (essentially Coulombic in nature) is hardly affected by the dihedral angle, especially since the π systems remained isolated even in the more planar conformations

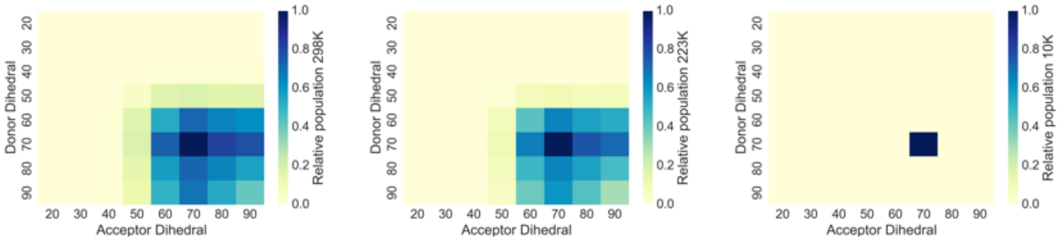

Figure S3. Relative population of **TNQ-ZnP$_{Ar}$-TAPD** , form the Boltzmann distribution, a) at 298 K and b) at 223 K (melting point of xylene) and c) at 10 K. For slower cooling rates that thermal distribution at which the conformations are effectively frozen is expected to correspond to different temperatures. In all cases the only minimum has a dominating contribution.

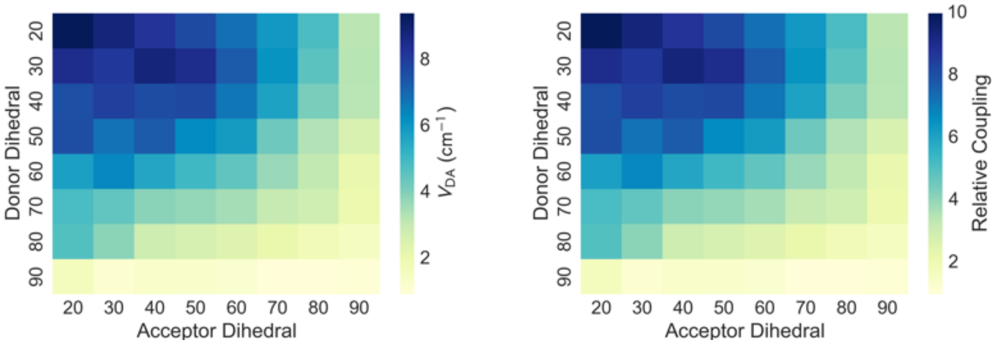

Figure S4. Relative GMH electronic coupling V_{DA} in **TNQ-ZnP$_{Ar}$-TAPD** as a function of the dihedral angles. As explained by the lack of extended conjugation, the dihedral angle has a modest effect on electronic coupling. Bis-*tert*-butylphenyl groups were removed to reduce the cost of simulation. 10-degree steps were used, for a total of 64 data points. Electronic coupling was calculated as described in the manuscript. The shape of the potential energy surface, in combination with the reduced effect of geometry on the electronic coupling, results in the Boltzmann-weighted electronic coupling being within 5% of the electronic coupling for the minimum-energy conformation.

11) Cambridge database analysis of the dihedral angles between meso-phenyls and porphyrin planes.

The following up-to-date Cambridge database search results are consistent with earlier studies.[S4]

In β-alkyl substituted porphyrins (bin size of 2°, total number of structures: 343)

Figure S5. Frequency of dihedral angle between porphyrin and phenyl planes in β-alkyl meso-(*ortho*-unsubstitutedphenyl)porphyrins

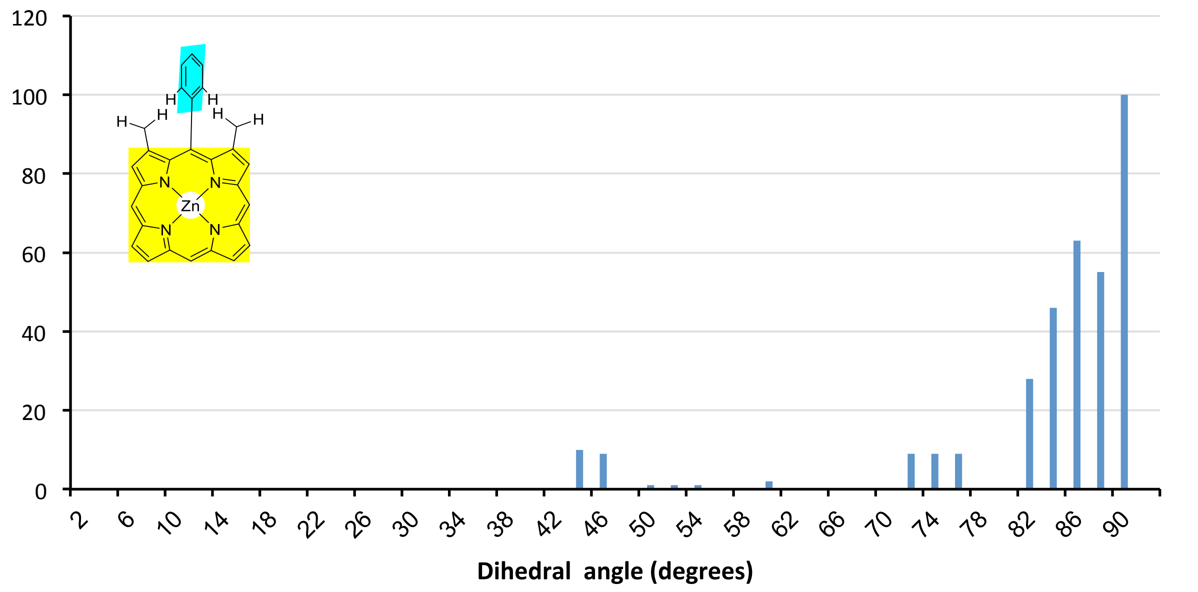

In β-unsubstituted porphyrins (bin size of 2°, total number of structures: 1032).

Figure S6. Frequency of dihedral angle between porphyrin and phenyl planes in β-H meso-(*ortho*-unsubstitutedphenyl)porphyrins

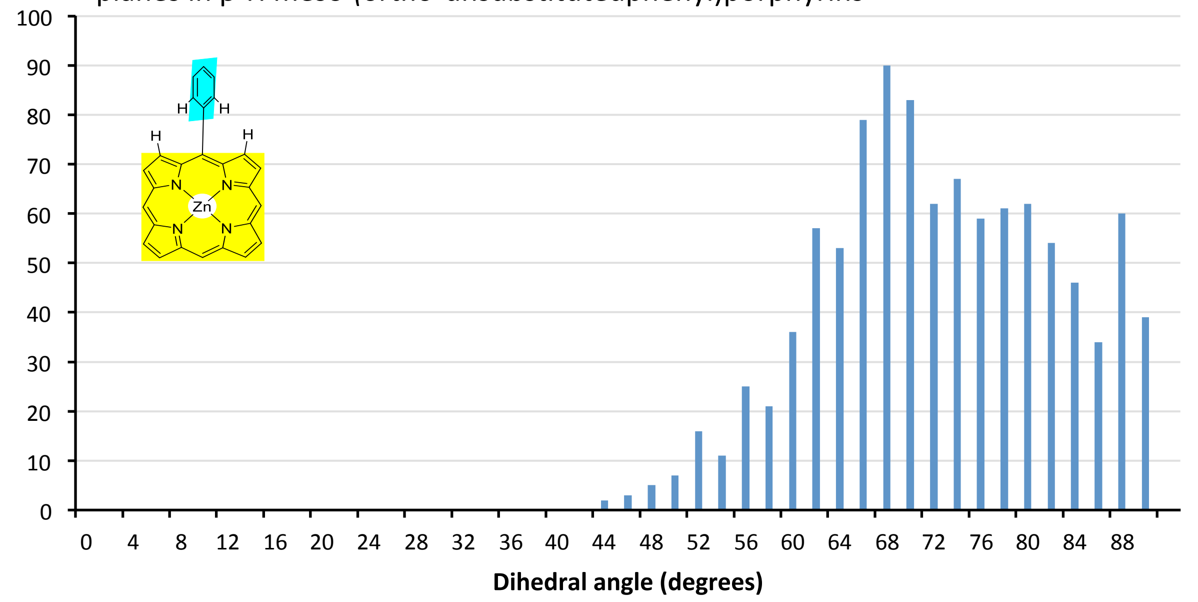

12) Methods and kinetic model fit of the EPR experiments

a. EPR methods:

EPR samples were prepared in 3.8 mm quartz EPR tubes, samples were degassed using several freeze pump thaw cycles and sealed under vacuum. The samples were stored at 77 K in the dark.

EPR experiment were carried out at 10 K on a Bruker ELEXSYS E580 spectrometer operating at X-band equipped with an MD5 dielectric resonator, Oxford Instruments continuous flow cryostat and ITC503 temperature controller. Laser excitation of the samples was provided by a Surelite OPO system, pumped by a Surelite I-20 Q-switched Nd:YAG laser, with a variable repetition rate. The photo-excitation wavelength was around 590 nm with a pulse energy of around 10 mJ per pulse and pulse width of 7 ns. Experiments on **TNQ-ZnP$_{Ar}$-TAPD**, **ZnTPP**, **C$_{60}$-ZnP$_{Ar}$-H** were carried out with the laser repetition rate at 20 Hz, while experiments on **C$_{60}$-ZnP$_{Ar}$-TAPD** was carried out with a repetition rate of 1 Hz, due to the long lifetime of the charge separation state.

b. Simulations:

Time resolved EPR spectra were simulated using the EasySpin toolbox[S5] in MATLAB, simulated spectra were used to determine the zero field splitting parameters (ZFS) and the polarization of the triplet states seen in **TNQ-ZnP$_{Ar}$-TAPD**, **ZnTPP** and **C$_{60}$-ZnP$_{Ar}$-TAPD**.

The time resolved EPR spectrum for both the **TNQ-ZnP$_{Ar}$-TAPD** triad and the reference sample **ZnTPP**, taken 1 µs after the laser flash, only showed a porphyrin triplet state. The porphyrin triplet for both EPR spectra was simulated using the same g tensors and relative sublevel populations, ZFS parameters used and relative populations of sublevels are shown in Table 1.

The time resolved EPR spectrum for the **C$_{60}$-ZnP$_{Ar}$-TAPD** triad showed a C$_{60}$ triplet and a spin polarized radical pair signal corresponding to the C_{60}^{-} anion. The C$_{60}$ triplet signal seen in the spectrum, taken 1.2 µs after the laser flash, was simulated with the parameters in Table 1. The ZFS parameters and the relative triplet polarization is consistent with literature values for a C$_{60}$-pyrrolidine mono-adduct, while the negative sign for *D* is consistent with that of C$_{60}$, determined by ENDOR.[S11]

**Table 1**	*D* / MHz	*E* / MHz	$p_x : p_y : p_z$	$g_x : g_y : g_z$	*Ref.*
**TNQ-^{3}ZnP$_{Ar}$-TAPD**	910[a]	287[b]	0.18 : 0.0 : 0.82	2.005 : 2.000 : 2.000	This work
**^{3}ZnTPP**	928[a]	295[b]	0.18 : 0.0 : 0.82	2.005 : 2.000 : 2.000	This work
**TNQ -^{3}ZnP- TAPD**	1079	327	0.35 : 0.00 : 0.65		S6
**^{3}ZnTPP**	893	294	0.0 : 0.0 : 1.0		S7
**^{3}ZnTPP**	888				S8
**^{3}C$_{60}$-ZnP$_{Ar}$-TAPD**	–272[c]	39	0.28 : 0.72 : 0.00	2.001 : 2.001 : 2.003	This work
^{3}C$_{60}$-pyrrolidine	–234	30	0.23 : 0.87 : 0.00		S9
^{3}C$_{60}$-pyrrolidine	270	–42			S10

[a] Gaussian distribution of *D* with a FWHM of 70 MHz
[b] Gaussian distribution of *E* with a FWHM of 20 MHz
[c] Gaussian distribution of *D* with a FWHM of 60 MHz.

c. Flash-delay experiment – kinetic model fit for CSS decay:

Change in population of sub-levels in 4-level system of Radical Pair:

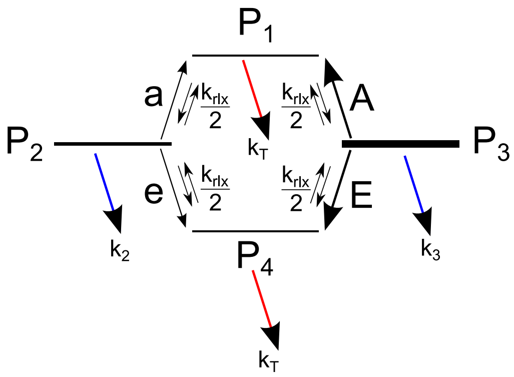

The time evolution of the radical pair peaks can be calculated using simple rate equations, for each sublevel in the 4-level system, as previously shown by Miura and Wasielewski.[S12]

$$\frac{dP_1}{dt} = (P_2 + P_3)\frac{k_{rlx}}{2} - P_1(k_{rlx} + k_T)$$

$$\frac{dP_2}{dt} = (P_1 + P_4)\frac{k_{rlx}}{2} - P_2(k_{rlx} + k_2)$$

$$\frac{dP_3}{dt} = (P_1 + P_4)\frac{k_{rlx}}{2} - P_3(k_{rlx} + k_3)$$

$$\frac{dP_4}{dt} = (P_2 + P_3)\frac{k_{rlx}}{2} - P_4(k_{rlx} + k_T)$$

Where

$$k_2 = k_s \cos^2\theta + k_T \sin^2\theta$$

$$k_3 = k_S \sin^2\theta + k_T \cos^2\theta$$

$$k_{rlx} = \frac{1}{T_1}$$

and k_s and k_T are the singlet and triplet recombination rates respectively.

We assume no recombination from the pure triplet levels (P_1 and P_4) and therefore the fast and slow decay seen experimentally must be due to k_S and k_{rlx}, therefore $k_S \gg k_{rlx}$. Since triplet recombination is not allowed we can say $k_2 \approx k_3 \approx k_S$.

The emission peak intensity is given by $I_{34}(t) = C_{34}\big(P_4(t) - P_3(t)\big)$ and where C_{34} is the transition probability.

Substituting for $P_4(t)$ and $P_3(t)$ gives:

$$I_{34}(t) = C_{34}\left(P_4 e^{-(k_{rlx})t} - P_3 e^{-(k_S)t}\right) \qquad \text{(S1)}$$

We solve equation (S1) analytically, with the addition of a Gaussian distribution in the rate constants k_{rlx} and k_S, and fit this model to the experimental trace. The fit result gives:

$k_S = k_{fast} = 1.8 \times 10^5\ \mathrm{s}^{-1}$ with one standard deviation of $0.7 \times 10^5\ \mathrm{s}^{-1}$.

$k_{rlx} = k_{slow} = 64\ \mathrm{s}^{-1}$ with one standard deviation of $32\ \mathrm{s}^{-1}$.

d. Spin polarized radical pair signal:

The central emission/absorption peak centered at *g* = 1.9999 can be attributed to the C_{60}^{-} anion, since the *g* factor matches those previously reported in literature and also as we do not expect to see the $TAPD^{+}$ cation (*g* = 2.0023–2.0030), due to hyperfine broadening.[S6,S13–16]

Magnetic field calibration using ^{31}P in ^{28}Si.

e. Estimation of the Quantum Yield for Formation of CSS_2 in C_{60}-ZnP_{Ar}-TAPD:

The quantum yield was estimated from the area of the signal from the charge-separated state (CSS), as a percentage of the total area of the time-resolved EPR spectrum (trEPR).

The central part of the trEPR spectrum was fitted to a 3rd order polynomial and two Gaussians (Gaussian 1, blue: emissive, and Gaussian 2, cyan: absorptive; Figure S7).

The quantum yield was estimated as the sum of the areas of the two Gaussians (Gaussian 1 + Gaussian 2) divided by the total area, giving a value of about 0.1.

This estimate assumes that formation of CSS_2 and the C_{60} triplet are the dominant decay channels, and that these two species have similar polarizations.

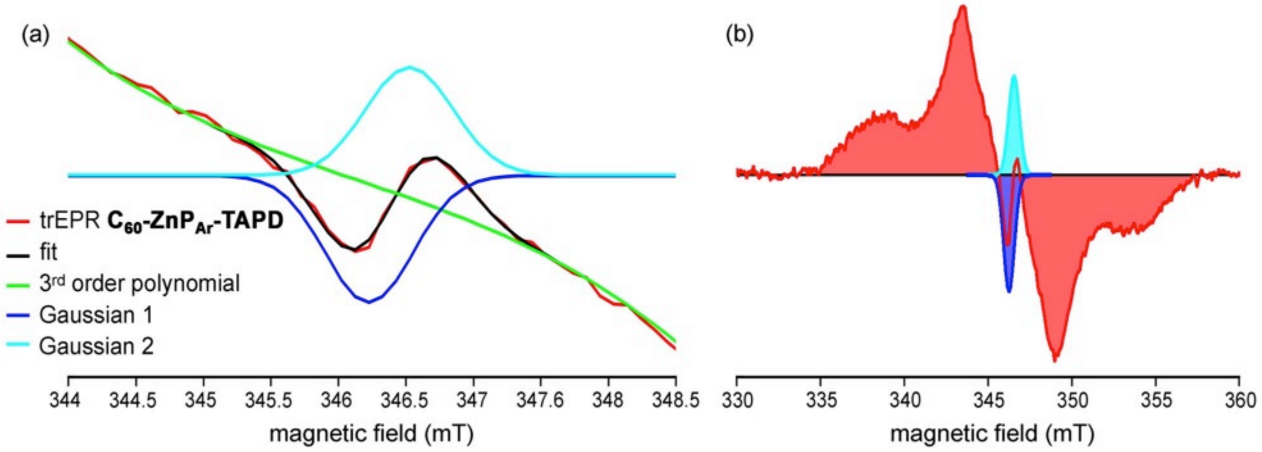

Figure S7. Time-resolved EPR spectrum **C_{60}-ZnP_{Ar}-TAPD**, taken 1.2 μs after laser flash. (a) Central feature, showing experimental data (red), fitting with 3rd order polynomial and two Gaussians (black), 3rd order polynomial (green), Gaussian 1 – emissive peak (blue) and Gaussian 2 – absorptive peak (cyan).
(b) Whole spectrum showing Gaussian 1 – emissive peak (blue line), Gaussian 2 – absorptive peak (cyan line). Absolute area taken of shaded regions.

13) Supporting References

S1 R. S. Coleman and M. A. Mortensen, *Tetrahedron Letters*, 2003, **44**, 1215.

S2 J. Plater, S. Aikena and G. Bourhill, *Tetrahedron*, 2002, **58**, 2405.

S3 N. K. S. Davis, A. L. Thompson and H. L. Anderson, *Org. Lett.*, 2010, **12**, 2124.

S4 H. L. Anderson, *Chem. Commun.*, 1999, **23**, 2323; H. N. Fonda, J. V. Gilbert, R. A. Cormier, J. R. Sprague, K. Kamioka and J. S. Connolly, *J. Phys. Chem.*, 1993, **97**, 7024.

S5 S. Stoll and A. J. Schweiger, *Magn. Reson.*, 2006, **178**, 42.

S6 A. Van Der Est, G. Fuechsle, D. Stehlik and M. R. Wasielewski, *Appl. Magn. Reson.*, 1997, **13,** 317.

S7 O. Gonen and H. Levanon, *J. Chem. Phys.,* 1986, **84**, 4132.

S8 B. Kirste, P. Tian, W. Kalisch and H. Kurreck, *J. Chem. Soc. Perkin Trans. 2*, 1995, 2147.

S9 S. Ceola, L. Franco, M. Maggini and C. Corvaja, *Photochem. Photobiol. Sci.*, 2006, **5**, 1177.

S10 G. Agostini, C. Corvaja and L. Pasimeni, *Chem. Phys.*, 1996, **202**, 349.

S11 G. J. B. van den Berg, D. J. van den Heuvel, O. G. Poluektov, I. Holleman, G. Meijer and E. J. J. Groenen, *J. Magn. Reson.*, 1998, **131,** 39.

S12 T. Miura and M. R. Wasielewski, *J. Am. Chem. Soc.*, 2011, **133**, 2844.

S13 M. R. Wasielewski, G. L. Gaines, M. P. O'Neil, W. A. Svec and M. P. Niemczyk, *Mol. Cryst. Liq. Cryst.,* 1991, **194**, 201.

S14 S.-H. Lee, A. G. Larsen, K. Ohkubo, Z.-L. Cai, J. R. Reimers, S. Fukuzumi and M. J. Crossley, *Chem. Sci.*, 2012, **3**, 257.

S15 M. Di Valentin, A. Bisol, G. Agostini and D. Carbonera, *J. Chem. Inf. Model.*, 2005, **45**, 1580.

S16 M. Di Valentin, A. Bisol, G. Agostini, P. A. Liddell, G. Kodis, A. L. Moore, T. A. Moore, D. Gust and D. Carbonera, *J. Phys. Chem. B,* 2005, **109**, 14401.
